# A Combination of Library Screening and Rational Mutagenesis Expands the Available Color Palette of the Smallest Fluorogen-Activating Protein Tag nanoFAST

**DOI:** 10.3390/ijms25053054

**Published:** 2024-03-06

**Authors:** Nadezhda S. Baleeva, Yulia A. Bogdanova, Marina V. Goncharuk, Anatolii I. Sokolov, Ivan N. Myasnyanko, Vadim S. Kublitski, Alexander Yu. Smirnov, Aidar R. Gilvanov, Sergey A. Goncharuk, Konstantin S. Mineev, Mikhail S. Baranov

**Affiliations:** 1 Institute of Bioorganic Chemistry, Russian Academy of Sciences, Miklukho-Maklaya 16/10, 117997 Moscow, Russia; 2 Laboratory of Medicinal Substances Chemistry, Institute of Translational Medicine, Pirogov Russian National Research Medical University, Ostrovitianov 1, 117997 Moscow, Russia

**Keywords:** fluorogen-activating proteins, FAST, fluorogen, fluorescence, nanoFAST

## Abstract

NanoFAST is the smallest fluorogen-activating protein, consisting of only 98 amino acids, used as a genetically encoded fluorescent tag. Previously, only a single fluorogen with an orange color was revealed for this protein. In the present paper, using rational mutagenesis and in vitro screening of fluorogens libraries, we expanded the color palette of this tag. We discovered that E46Q is one of the key substitutions enabling the range of possible fluorogens to be expanded. The introduction of this and several other substitutions has made it possible to use not only orange but also red and green fluorogens with the modified protein.

## 1. Introduction

The expansion of the phenomenological palette of light-emitting molecules throughout history has formed the basis of the biological cognition as we observe it now. The first fluorescent microscopes for biologists were created by Heinrich Lehmann of the Carl Zeiss factory and independently by Otto Heimstädt from the Carl Reichert firm at the beginning of 20th century and were initially used for autofluorescent specimens [1]. Staining methods with various synthetic fluorophores were developed later, making fluorescent microscopy popular. In the early 1940s, Coons and colleagues were the first to conjugate antibodies to a fluorescein fluorophore, developing an immunofluorescence method to visualize proteins–antigens in tissues [2], which gave synthetic fluorophores a way to stain with extreme specificity. Although, they mostly worked with fixed, permeabilized objects. Fifty years later, Martin Chalfie and colleagues created genetically encoded fluorescent labeling with *Aequorea victoria* Green Fluorescent Protein [3], and over the years, variations in GFP and other fluorescent proteins have became the most established and are a powerful way to visualize living and fixed samples [4,5]. Currently available in multiple colors, fluorescent proteins (FPs) enable the visualization and tracking of proteins of interest in various cell types and organisms. FP-based biosensors shed light on cellular physiology, and the development of sophisticated microscopy techniques lets scientists look at cellular structures beyond the diffraction limit using super-resolution microscopy. However, FPs have a number of drawbacks: FPs need oxygen to form a chromophore and cannot be used under anaerobic conditions [6]; this process of oxygen-dependent chromophore maturation is quite long (up to an hour) and is not compatible with the observation of fast processes in real time [7]. Most importantly, fusion with FPs significantly increases the total molecular weight of the observed protein. The median length of a human protein is 375 amino acid residues [8], while the eGFP label has a length of 238 residues. The addition of a 27 kDa FP tag has a well-documented list of physiological and biochemical impairments caused by this [9,10,11,12]. Significant reduction in tag size seems impossible in the case of classical fluorescent proteins due to their conserved structure.

The smallest fluorescent genetically encoded tags have been developed based on alternative proteins that do not form their own chromophore and act as fluorogen-activating proteins (FAPs). Fluorogens are non-fluorescent molecules in the free state which become highly fluorescent only upon entering a protein pocket. Some FAPs bind endogenous fluorogens such as flavin, biliverdin, and bilirubin [13,14,15,16], but the others need an externally added synthetic fluorogen [17,18,19]. All of them enable visualization of processes under anaerobic conditions [20,21,22]. The main disadvantages of endogenously present fluorogens are the color palette limitation and the risk of disturbing cellular homeostasis by seizing the physiologically important molecules. Proven to be non-toxic, externally added fluorogens, on the other hand, offer a great opportunity to develop a vast color pallet employing synthetic chemistry.

The fluorescence-activating and absorption-shifting FAST tag (14 kDa, 126 amino acid residues) has been engineered by *Halorhodospira halophila* photoactive yellow protein (PYP) mutagenesis to act like a non-covalently binding FAP [18]. Non-covalent binding allows unique rapid on–off switching by addition and removal of fluorogen, making the system very flexible and providing opportunities for multiplexing. FAST-tag labeling is a great example of a method in which biology and chemistry combine their strengths. Synthetic chemistry has provided a range of fluorogens with a wide range of absorption/emission properties [23,24,25,26,27]. The molecular biology toolbox has provided several FAST mutants that have been optimized to bind other ligands [28,29] and a FAST-based split fluorescent reporter system [30].

In previous work on the fluorogen-activating protein FAST, we [24,25] and others [27,28] have shown several effective approaches to create new protein–fluorogen pairs. First, using large libraries of compounds, we identified new pairs with different colors based on the same protein but different fluorogens [24,25]. Second, using both random and site-directed mutagenesis, new pairs with improved properties were created with different proteins for individual specific fluorogens [27,28].

**Scheme 1:**
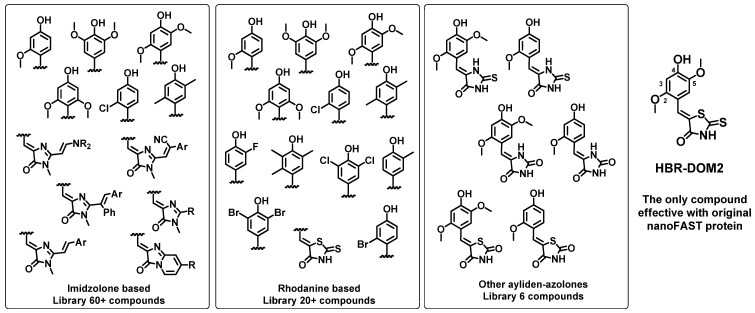
Fluorogens library tested in the present study (See Supplementary Materials for more details Table S2.1 and Part S7). The numbers on the structure of the fluorogen indicate the numbering of substituents in the arylidene fragment, discussed in the text.

Recently, based on the NMR structure of FAST, we created a smaller version of the parent FAST protein, nanoFAST, which is only 98 amino acid residues long [19]. The only drawback of the shortened version is that it binds a single fluorogen, **HBR-DOM2** (Scheme 1) [31]. In the present work, we used the advantage of the FAP hybrid nature and developed a range of new fluorogens for nanoFAST-based color multiplexing.

## 2. Results and Discussion

In this study, we decided to combine the two abovementioned approaches to search for new protein–fluorogen pairs based on the nanoFAST protein.

First, we created a library of fluorogenic compounds that are promising ligands for nanoFAST protein variants. This library includes many of our previously synthesized arylidene-imidazolones [24,25] as well as arylidene-rhodanines [28]. We also synthesized a number of novel compounds, including various azolone derivatives—hydantoins, thiohydantoins, and others (Scheme 1). All created fluorogens contain a hydroxy group in the para position of the arylidene fragment, since it interacts with the nanoFAST residues E46 and Y42, which provide binding and fluorescence enhancement [18,19]. It is important to point out that hereinafter we use the amino acid numbering adopted for the original FAST protein (as well as the parent PYP) for correct comparison with previously published data. It is noteworthy that in the case of nanoFAST family proteins, such numbering does not take into account the absence of the first 27 amino acids. We also introduced methoxy groups into the second, second and fifth, or third and fifth positions of the arylidene moiety because these substitutions were found to be effective for the full-length FAST protein. Halogen atoms or methyl groups were also introduced into all these positions. Particular attention was paid to the moieties at the second and fifth positions, since the similar compounds interact best with the full-length protein. Furthermore, 2,5-dimethoxy-4-hydroxy-arylidene-rhodanine (compound **HBR-DOM2**, Scheme 1) was the only compound that worked well as a fluorogen for the original nanoFAST. Thus, halogen atoms or methyl groups were introduced into all these positions.

**Figure 1:**
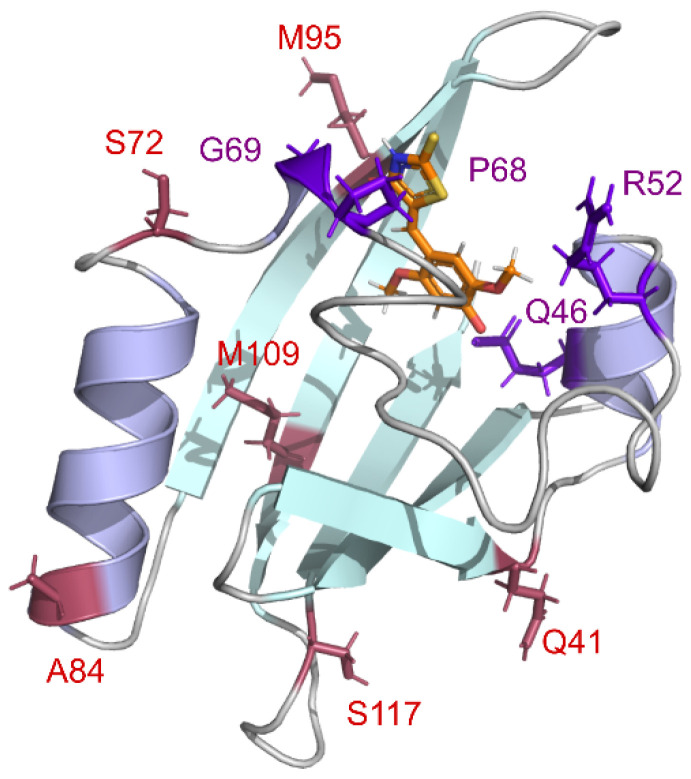
Positions for the rational single-point mutations (purple) and the mutations adapted from the tFAST protein (red) [26] are shown on the spatial structure of nanoFAST in complex with HBR-DOM2 (PDB code A8O0) [19].

Second, we created a series of mutant variants of the nanoFAST protein. For this purpose, we used data on improved versions of the full-length FAST protein obtained through directed evolution: iFAST (V107I) [32], greenFAST (P68T, G77R) [28], redFAST (F28L E46Q) [28], frFAST (F62L, D71V, P73S, E74G, V107I) [27], pFAST (A30V, Q41L, S72T, V83A, M95T, M109L, S117R) [26], tFAST (Q41K, S72T, A84S, M95A, M109L, S117R) [26]. Additionally, we previously used a rational approach to reveal the important role of the residue at position 52 in the case of full-sized FAST paired with imidazolone-based dyes [19]. Various polar groups at this position can interact with the azolone moiety of the fluorogen and improve binding or brightness. Therefore, we decided to introduce eleven differently functionalized amino acids at the homologous position of nanoFAST protein. Based on our structural data [19], residues 68 and 69, located at the entrance to the binding pocket, may also be responsible for fluorogen binding. Thus, we have introduced several substitutions in these positions. In sum, we selected more than two dozen proteins whose amino acid sequences are presented in Supplementary Materials, Table S1.1. Key substitutions are presented in Figure 1.

All the proteins were produced in *E. coli* and purified according to the protocols previously suggested for the nanoFAST protein [31] (Supplementary Materials Part S1). In most of the cases, the yields and lifespans of the proteins were not different from the original ones. However, it is interesting to note that the introduction of substitutions that was previously extremely effective against a wide range of ligand variants of the full-length FAST protein (pFAST) in the nanoFAST context led to significant destabilization of the protein and a dramatic decrease in its bacterial production (Table S1.1), which forced us to exclude this variant from further analysis.

**Figure 2:**
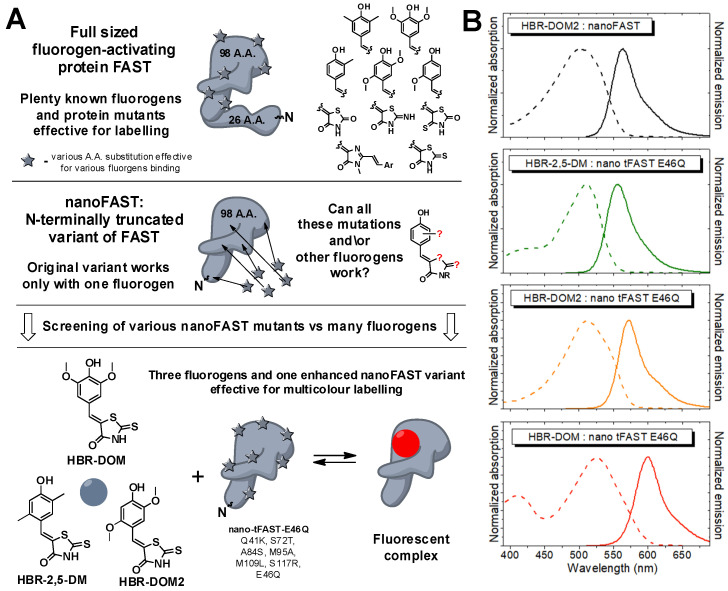
(**A**). Design of the presented study. (**B**). The normalized absorption (dashed lines) and emission (solid lines) spectra of various fluorogens in complexes with nanoFAST and nano-tFAST-E46Q proteins. Sign "?" means various substituents in the molecule of the possible fluorogen arylidene-azolone.

**Scheme 2:**
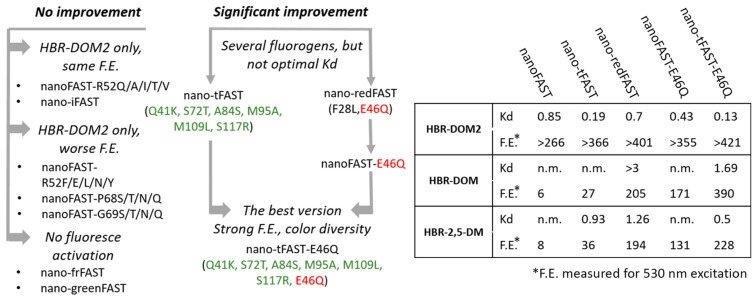
Results of nanoFAST variants screening and design (F.E.—fluorescent enhancement revealed by plate reader screening; n.m.—not measured). Amino acid substitutions taken from the tFAST and redFAST proteins, are marked in green and red respectively.

Next, we screened these fluorogen library and nanoFAST variants in an all-versus-all format using a plate reader (Figure 2, Scheme 2, Supplementary Materials Part S2, Tables S2.2–S2.28). We found that most of the proposed nanoFAST variants and fluorogens do not form effective pairs. Moreover, most of the proposed single substitutions in positions 52, 68, and 69 led to a significant decrease in fluorescence enhancement with the previously proposed fluorogen of the nanoFAST protein—**HBR-DOM2**. At the same time, various variants found earlier by directed evolution retained binding to this ligand (Scheme 2, Supplementary Materials Part S3, Table S3.1). Such a diversity of proteins binding to a single fluorogen could potentially be used for Fluorescence Lifetime Imaging Microscopy (FLIM) multiplexing [28]. However, we found that complexes of **HBR-DOM2** fluorogen with various proteins are characterized by relatively close fluorescence lifetimes of about 3.6–4.1 ns (Supplementary Materials Part S4).

Nevertheless, screening of two proteins (nano-redFAST and nano-tFAST) revealed a number of promising interactions with arylidene-rhodanine fluorogens (Supplementary Materials Parts S2 and S3, Tables S2.6, S2.8, S3.2–S3.6). The nano-redFAST protein contains the E46Q substitution located in the active center and is directly involved in the interaction with the phenolic group of the ligands. This protein was effective against a number of fluorogens from the arylidene-rhodanine family, which may indicate the high importance of this substitution (Supplementary Materials, Part S3, Table S3.6). We tested its direct impact using the nanoFAST-E46Q variant with this single substitution (Supplementary Materials Table S2.27) and found that E46Q is responsible for the binding improvement. However, for the fluorogen with the longest emission wavelength (**HBR-DOM**), the binding was quite poor (Kd > 3 μM, see Table S3.6), so further protein optimization was required (Scheme 2).

Based on the data obtained, we decided to combine these substitutions and created a protein nano-tFAST-E46Q (Q41K, S72T, A84S, M95A, M109L, S117R, E46Q). A study of this protein showed that it is able to bind and enhance the fluorescence of several fluorogens from the arylidene-rhodanine group (Supplementary Materials, Parts S2 and S3, Tables S2.28 and S3.7). Among these compounds, we selected three fluorogens with the most distant colors—orange **HBR-DOM2**, green **HBR-2,5-DOM**, and red **HBR-DOM**. A stepwise fluorescence enhancement increase under screening conditions for these compounds with various proteins is presented in Figure S2.2. A remarkable fluorescence enhancement was also observed for various halogenated derivatives (see Table S3.6 for their structure); however, all of them revealed a detectable fluorescence in the free form (Figure S2.1) and their color properties did not differ substantially compared to the three listed compounds. The parent blueshifted fluorogen **HMBR** also seemed promising, but its binding to all proteins was quite weak (Figure S2.2, Table S3.3). Among the newly created substances, the derivatives of other azolones were initially considered as most prospective since their analogs were previously successfully utilized for p- and tFAST [26]. However, taken with a truncated protein, all of them appeared inefficient. Additionally, all the imidazolone derivatives also remained dim in complex with nanoFAST, which supports our previous hypothesis that the NH moiety of heterocycle is important for binding and its capability to serve as an acceptor of the non-canonical hydrogen bond with adjacent G69 and P97 is inherent only for rhodanines [31].

Next, we studied **HBR-DOM2**, **HBR-2,5-DOM**, and **HBR-DOM** compounds in pairs with the nano-tFAST-E46Q protein in more detail—Table 1, Supplementary Materials Parts S3–S5.

All resulting pairs were characterized by fairly close dissociation constants, typical for fluorogen–FAST complexes. Such constants enable reversible binding that significantly increases the photostability of the fluorescent signal due to the possible exchange of the dye in the case of its photobleaching. High values of fluorescence quantum yield (FQY) and molar absorption coefficients provide sufficiently good brightness suitable for fluorescent labeling in living systems for all the pairs. Similar values were previously obtained for many other fluorogen–protein pairs successfully used in practice [23,24,25,26,27,28,29,30,31]. Thus, we significantly expand the color palette of fluorogens applicable for the truncated nanoFAST protein. The pairs we created based on the fluorogens **HBR-2,5-DM** and **HBR-DOM** can be used not only in the orange region but also in the green and red channels, respectively. We also found that fluorescent lifetimes of these complexes of nano-tFAST-E46Q protein differ significantly (Table 1). Thus, we expanded not only the color but also the lifetime palette of this tag.

**Figure 3:**
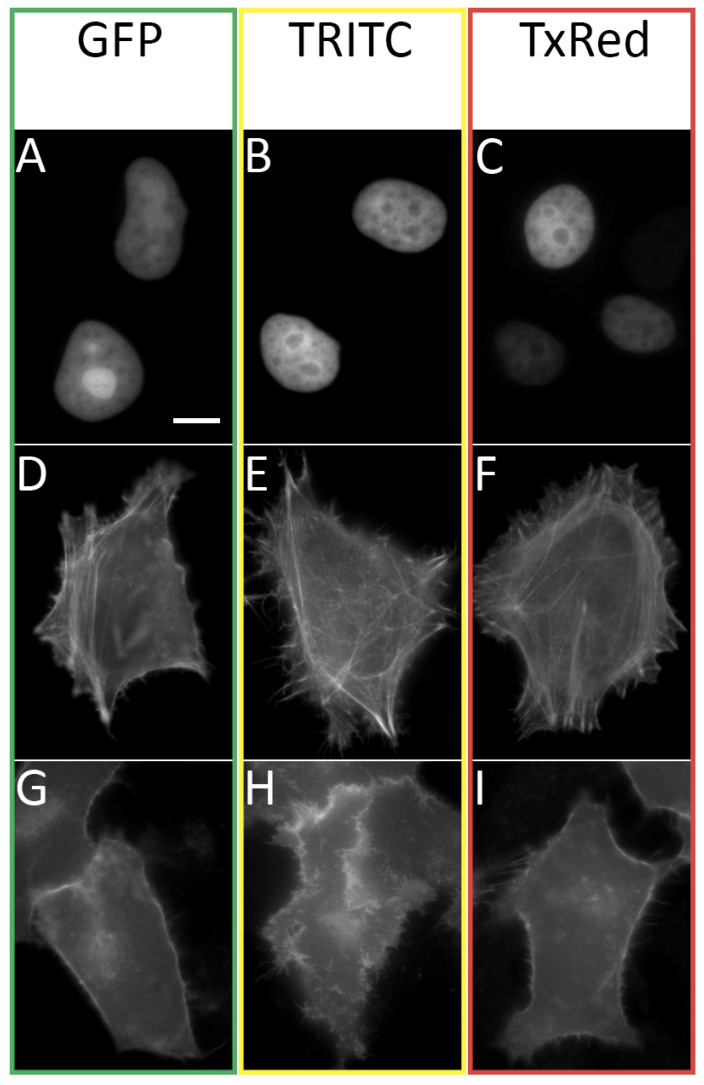
Live-cell imaging with a set of fluorogens in different filter sets. HeLa Kyoto cells transiently transfected with nano-tFast-E46Q-H2B (**A**–**C**), nano-tFast-E46Q-LifeAct (**D**–**F**), and nano-tFast-E46Q-GAP43 (**G**–**I**). Cells were imaged using GFP filter in the presence of 5 μM **HBR-2,5-DM** (**A**,**D**,**G**), TRITC filter in the presence of 5 μM **HBR-DOM2** (**B**,**E**,**H**), TxRed filter in the presence of 5 μM **HBR-DOM** (**C**,**F**,**I**). Scale bar is 10 μm.

Thus, using fluorescence microscopy of living cells transiently transfected with various FAST fusions (Figure 3), we showed that the three substances **HBR-2,5-DM**, **HBR-DOM2**, and **HBR-DOM** can effectively be used in the GFP, TRITC, and TxRed channels. Nevertheless, like all other previously created fluorogens of the FAST protein, the fluorogens proposed in this work had wide emission and absorption spectra. This property can lead to the fluorescent signal entering adjacent channels, which requires the use of narrower filters and careful selection of multicolor labeling conditions. However, it can be reliably stated that the fluorogen **HBR-2,5-DM** does not give a signal in the TxRed channel, and the fluorogen **HBR-DOM** does not give a signal in the GFP channel (Supplementary Materials Part S6, Figure S6.1).

The proposed dyes easily penetrated the cell membrane and nuclear envelope and labeled nano-tFast-E46Q fused to H2B-histones. We also showed the possibility of tagging more complex structures—actin with nano-tFast-E46Q-LifeAct and plasmatic membrane with nano-tFast-E46Q-GAP43. Cellular morphology was not affected in any way when labeled with all of these fuses, and the dyes themselves did not exhibit significant toxicity in the course of the imaging experiment. The dyes used also did not exhibit off-target labeling in the solution or in membranes, which correlates well with the data obtained previously for other rhodanine fluorogens.

## 3. Materials and Methods

### 3.1. General

Reagents available from commercial sources were used without additional purification. Flash column chromatography was performed with Silica gel 60 (Merck, Darmstadt, Germany). Thin layer chromatography (TLC) was performed on silica gel 60 F_254_ alu foil (Merck). Visualization of TLC was performed using UV light and potassium permanganate staining. NMR study was performed with 700 MHz Bruker Avance III NMR at 303 K and Bruker Fourier 300 (Billerica, MA, USA). Chemical shifts are reported relative to residue peaks of DMSO-d_6_ (2.51 ppm for ^1^H and 39.5 ppm for ^13^C). Descriptions of substances and spectra are given in Supplementary Materials Parts S7 and S9. SMP 30 apparatus was used for melting points measurements. For high-resolution mass spectra (HRMS) study, a Triple TOF 5600+ by AB Sciex instrument (Framingham, MA, USA) using electrospray ionization (ESI) with nanoSpray III Ion Source (Framingham, MA, USA) was used. The measurements were performed in a positive ion mode with interface capillary voltage of 2.3 kV and declustering potential of 50 V. Ion source gas flow was set at 15 arb; curtain gas flow was set at 25 arb. A syringe injection was used for solutions in a mixture of acetonitrile and aqueous formic acid (0.1% vol%) at a flow rate of 1 µL/min. Nitrogen was applied as a dry gas; interface temperature was set at 150 °C. All spectral data are presented in Supplementary Materials Parts S7 and S9. All compounds used in the study were prepared by procedures presented below or were taken from stock of our laboratory. All solid fluorogens were dissolved in DMSO (Sigma Aldrich, St. Louis, MO, USA, “for molecular biology” grade) in 5 mM concentration and stored in a dark place at −20 °C for no more than 3 months. UV-VIS spectra were recorded on a Varian Cary 100 spectrophotometer (Santa Clara, CA, USA). Fluorescence excitation and emission spectra were recorded on an Agilent Cary Eclipse fluorescence spectrophotometer (Santa Clara, CA, USA). Some spectra were recorded on the Tecan Infinite 200 Pro M nano dual mode plate reader (Männedorf, Switzerland). This reader was also used for screening and dissociation constant measurements.

### 3.2. Synthesis

#### 3.2.1. Preparation of (Z)-5-(4-Hydroxybenzylidene)-2-Thioxothiazolidin-4-One [19]

2-thioxothiazolidin-4-one (2.4 mmol) was mixed with corresponding aromatic aldehyde (2 mmol) and anhydrous sodium acetate (6 mmol) in 5 mL of glacial acetic acid. The mixture was heated in an oil bath at 110 °C for 2–5 h. The progress of the reaction was monitored with TLC (CHCl_3_/EtOH, 100/5). After reaction completion, the mixture was cooled to room temperature, poured into 100 mL of water, and acidified to pH = 2 with 5% hydrochloric acid solution. A suspension was filtered off and washed twice with 30 mL of water. The solid was dissolved in a mixture of CH_2_Cl_2_/MeOH (10/1) and n-hexane was added. The resulting mixture was stored at 5 °C for 2 h. The precipitate was filtered and washed with hexane (3 × 10 mL). The powder was dried under reduced pressure to afford pure target compounds.

#### 3.2.2. Preparation of 5-(4-Hydroxybenzylidene)-2-Thioxoimidazolidin-4-Ones [26]

Corresponding aromatic aldehyde (3 mmol), 2-thiohydantoin (3.6 mmol), and anhydrous sodium acetate (12 mmol) were dissolved in glacial acetic acid (10 mL). The reaction mixture was heated to 115 °C in the oil bath and left to stir at the indicated temperature for 5–7 h. After completion, the reaction mixture was poured into 250 mL of water. The precipitate was filtered and washed with water (3 × 10 mL). The powder was dried under reduced pressure to afford pure target compounds.

#### 3.2.3. Preparation of 5-(4-Hydroxybenzylidene)-Imidazolidine-2,4-Diones [26]

Hydantoin (2 mmol) was dissolved in hot water (70 °C, 2 mL). A saturated sodium bicarbonate solution was added dropwise to the solution of hydantoin until its pH turned to 7. The reaction flask was submerged into a preheated oil bath (90 °C). A suspension of a corresponding aromatic aldehyde (2 mmol) in EtOH (2 mL) was added. The reaction mixture was stirred for 15–20 h at 90 °C. The reaction mixture was cooled to room temperature and diluted with water (10 mL). The precipitate was filtered and washed with water (2 × 10 mL) and aqueous ethanol (40% vol., 1 × 7 mL). The powder was dried under reduced pressure to afford pure target compounds.

#### 3.2.4. Preparation of 5-(4-Hydroxybenzylidene)-Thiazolidine-2,4-Diones [26]

A corresponding aromatic aldehyde (2 mmol) and thiazolidine-2,4-dione (2 mmol) were dissolved in hot toluene (4 mL). Benzoic acid (0.26 mmol) and piperidine (0.26 mmol) were added to the hot solution. The reaction mixture was allowed to stir at 109 °C for 3–4 hours. After completion, the reaction mixture was cooled to room temperature and diluted with diethyl ether (10 mL). The precipitate was filtered off, washed with diethyl ether (3 × 10 mL). The powder was dried under reduced pressure to afford pure target compounds.

#### 3.2.5. Preparation of 5-(Z)-Benzylidene-2-Methyl-3-Alkyl/Aryl-3,5-Dihydro-4H-Imidazol-4-Ones [38]

The corresponding aromatic aldehyde (10 mmol) was dissolved in CHCl_3_ (50 mL) and mixed with methylamine solution (40% aqueous, 2.5 mL), pyrrolidine (7 mg. 0.1 mmol), and anhydrous Na_2_SO_4_ (10 g). The mixture was stirred for 48 h at room temperature, filtered, and dried over the additional Na_2_SO_4_. The solvent was evaporated and corresponding ethyl((methoxy)amino)acetate (20 mmol) was added to the residue (5–10 mL of methanol was also added if complete dissolution was not observed). The mixture was stirred for 24 h at room temperature, solvents were evaporated, and the product was purified by column chromatography (CHCl_3_-EtOH).

#### 3.2.6. Preparation of (Z)-2-(4-Hydroxy-2-Methoxybenzylidene)Imidazo[1,2-a]Pyridin-3(2H)-Ones [25]

Corresponding glycine hydrochloride (6 mmol) was dissolved in PCl_3_ (7 mL) and the mixture was refluxed for 5 h. The solvent was evaporated, and a solution of 4-hydroxy-2-methoxybenzaldehyde (5.4 mmol) and triethylamine (7.2 mmol) in pyridine (6 mL) was added. The obtained mixture was heated to 70 °C for 12 h. Then, the solvent was evaporated and 100 mL of DCM was added. The resulting solution was washed by PBS (2 × 30 mL, 0.5 M, pH = 7) and brine (30 mL) and dried over Na_2_SO_4_. The solvent was evaporated and the crude product was purified by column chromatography (CHCl_3_-EtOH).

#### 3.2.7. Preparation of 5-(Z)-Benzylidene-2-(E)-Arylvinyl-3-Methyl-3,5-Dihydro-4H-Imidazol-4-Ones [19,25]

To the solution of 5-(Z)-benzylidene-2,3-dimethyl-3,5-dihydro-4H-imidazol-4-one (1 equiv.), dioxane anhydrous zinc chloride (0.2 equiv.) and corresponding aldehyde (1.2 equiv.) were added. The mixture was refluxed for 1–6 h. The progress of the reaction was monitored with TLC (CHCl_3_/EtOH, 100:5). After completion of the reaction, the solvent had evaporated. The mixture was dissolved in EtOAc and washed by EDTA solution (0.5%), water, and brine. The mixture was dried over Na_2_SO_4_. The solvent was evaporated and the product was purified by column chromatography (CHCl_3_-EtOH, typically 100:5).

### 3.3. Proteins Production

All genes were obtained from the “Cloning Facility” (Moscow, Russia) as pEXPR_002 expression vector. At the 3′ end of the target genes, the DNA sequence encoding six histidines and three glycines was placed. The DNA sequence was confirmed with the use of automatic sequencing. Proteins purification was made as described earlier [19]. Briefly, the minimal salts medium M9 was used for production of recombinant proteins employing *E. coli* BL21(DE3) strain. Cells were grown at 37 °C, 250 rpm, until OD600 reached 0.6. The target protein synthesis was induced with 0.3 mM IPTG and cultivation continued for 4–5 h. Cells were harvested and kept at −20 °C. Cells were lysed by ultrasonication on ice, and target proteins were purified employing Ni^2+^ chelate affinity chromatography followed by dialysis through membrane with a molecular weight cutoff of 3.5 kDa against PBS buffer at room temperature and stored at +4 °C (Figures S1.1 and S1.2).

### 3.4. Screening In Vitro

Screening results are presented in Supplementary Materials, Part S2. Optical properties for Table S2.1 were investigated for 10 µM solutions of fluorogens in water (pH~5) using Varian Cary 100 spectrophotometer and Agilent Cary Eclipse fluorescence spectrophotometer; for most of the listed compounds, they were measured in our previous works [19,24,25]. Fluorogen–protein binding efficiency (fluorescence enhancement, Tables S2.2–S2.28) was tested using solutions containing 10 µM of corresponding fluorogens, 1 µM of nanoFAST variants in the PBS buffer (pH 7.4, Amresco, Solon, OH, USA) and 10 µM blank solution of pure corresponding fluorogens in the PBS buffer (pH 7.4, Amresco). Fluorescence intensity enhancement was defined as the ratio of fluorescence intensity of fluorogen + protein solution to the fluorescence intensity of free fluorogen solution recorded on Tecan Infinite 200 Pro M nano dual-mode plate reader. Both solutions were excited at similar wavelengths listed in the tables presented in Supplementary Materials—430, 480, 530, and 580 nm. This intensity was obtained as the total integral (sum of values) of fluorescence spectra. Due to the concentrations used, there can be non-saturated conditions. Correction to the absorbance intensity was not made. In several cases, when the fluorescence of the complexes was too high, the device went off scale. To indicate such cases, the symbol “>” is added to the enhancement value. It is important to note that such a symbol does not always mean a good fluorescence enhancement upon binding, since high emission of the complex may be accompanied by high fluorescence of the free fluorogen. For example, many values like “>65” obtained for halogenated rhodanines (see Table S3.6) mean exactly this situation. In general, based on our previous experience [19,24,25], we can say that the really successful pairs (high quantum yield and fairly low Kd) are characterized by more than 100 fold enhancement for any individual excitation wavelengths in the presented experimental protocol.

### 3.5. Spectra of Complexes

Optical properties of complexes for Figure 2 and Supplementary Materials Part S5 (Figure S5.1) were investigated using 5 µM solutions for absorption spectra and 0.5 µM for emission spectra in the PBS buffer (pH 7.4, Amresco). Proteins were added in such amount that indicated a fraction of the protein/fluorogen complex of α ≥ 95%. The total protein concentration for each complex was calculated using the equation:

(1) $$\left[{Pr}_{0}]=\frac{K_{D}\times \left(\alpha \times \left[{Chr}_{0}\right]\right)}{\left[{Chr}_{0}\right]-\left(\alpha \times \left[{Chr}_{0}\right]\right)}+(\alpha \times \left[{Chr}_{0}\right]\right)$$

where *K_D_*—dissociation constant, [*Chr*_0_]—total fluorogen concentration, and *α*—fraction of the protein/fluorogen complex.

All spectra are presented in Figure 1 and Supplementary Materials Part S5.

### 3.6. Dissociation Constants Measurements

Dissociation constants were determined by spectrofluorometric titration of proteins by fluorogen solutions with various concentrations on the Tecan Infinite 200 Pro M plate reader. The protein concentration was 0.10 µM. Data represent mean ± sem (*n* = 3–5). The titration experiments were performed at 25 °C in pH 7.4 PBS (pH 7.4, Amresco). The fitting was performed using OriginPro 8.6 software. All titration curves and other data are presented in Supplementary Materials Part S3, Figures S3.1–S3.6.

### 3.7. Fluorescence Quantum Yields Determination

Fluorescence quantum yields were measured according to the well-known previously described procedure [39] with the use of Rhodamine 6G (for complexes of **HBR-DOM2** and **HBR-2,5-DM**) and Rhodamine 101 (for complexes of **HBR-DOM**) as standards. The fluorogens concentration was 5 µM for absorption and 1.67, 0.5, and 0.167 µM for emission, and proteins were added in such a concentration that indicated α > 95% (see Equation (1)).

### 3.8. Fluorescence Lifetime Measurements

Fluorogen **HBR-DOM2** was mixed (from stock solutions in DMSO 10 mM) with proteins in the PBS buffer at room temperature. Final concentrations were 10 μM and 0.2 μM for proteins and fluorogens, respectively. Measurements were made using a time-resolved miniTau fluorescence spectrometer (Edinburgh Instruments, Livingston, UK) in a 50 ns window divided into 1024 time channels. The fluorescent was excited using EPL-450 picosecond laser (Edinburgh Instruments, Livingston, UK) with a central emission wavelength of 445.6 nm and repetition rate of 20 MHz. The photons were counted in the spectral range of 575–625 nm. The data processing, visualization, and determination of χ2 (Pearson’s test) were carried out using Fluoracle 2.5.1 software (Edinburgh Instruments, Livingston, UK). The fitting of fluorescent decay curves was carried out by deconvolution with the instrument response function (IRF-based fit). All decay curves are given in Supplementary Materials Part S4, Figures S4.1–S4.7.

### 3.9. DNA Cloning

Coding sequence of nano-tFast-E46Q in Level 0 plasmid was obtained from the “Cloning Facility” (Russia). Nano-tFast-E46Q fused with H2B histone protein sequence, LifeAct actin-binding peptide sequence, and Gap43 membrane anchored short peptide sequence were cloned by Golden Gate assembly, following the MoClo syntax. The construct was put under control of a CMV promoter and a SV40 poly(A) sequence. BpiI (BbsI) and Eco31I (BsaI) restriction endonucleases (Thermo Scientific, Waltham, MA, USA) and T4 DNA ligase (Evrogen LK001, Moscow, Russia) were used for the cloning procedure.

### 3.10. Cell Culture and Transfection

HeLa Kyoto cells (ATCCs) were grown in Dulbecco’s modified medium (DMEM; PanEco, Moscow, Russia) with 10% (*v*/*v*) fetal bovine serum (PanEco) containing 50 U/mL penicillin and 50 μg/mL streptomycin (PanEco) at 37 °C and 5% CO_2_ and seeded onto a 35 mm glass-bottomed culture dish (SPL Life Sciences, Pocheon-si, Gyeonggi-do, Republic of Korea) 24 h before transfection. Polyethylenimine (PEI, #23966-1, Polysciences, Warrington, PA, USA) was used as a transfection reagent. For transfection, DMEM was changed for Opti-MEM one hour before the procedure. In total, 3 μL of PEI was mixed with 250 μL of Opti-MEM per dish, and in a separate tube 1 μg of plasmid DNA was mixed with 250 μL of Oti-MEM. Mixtures were incubated for 5 min, and then PEI- and DNA-containing media were mixed and the resulting solution was incubated for 20 min. These procedures were carried out at room temperature. PEI-DNA mixture was added to cells dropwise, cells were incubated for 3 h at 37 °C and 5% CO_2_, and transfection media were replaced with DMEM complete.

### 3.11. Live Cell Imaging

The imaging experiments were carried out 24 h after transfection using a BZ-9000 inverted fluorescence microscope (Keyence, Osaka, Japan) in 2 mL of Hanks’ Balanced Salt Solution (PanEco) with 10 mM HEPES (Sigma, Darmstadt, Germany), pH 7.3, and 5 μM fluorophore (from 10 mM DMSO stock solution) at room temperature. The imaging was performed with a 60 × PlanApo 1.40 NA oil objective (Nikon, Melville, NY, USA); GFP-B filter (Keyence, Ex. 470/40 nm, DM 495 nm, BA 535/50 nm), TRITC filter (Keyence, Ex. 540/25 nm, DM 565 nm, BA 605/55 nm), and TxRed filter (Keyence, Ex. 560/40 nm, DM 595 nm, BA 630/60 nm) were used. The resulting images were processed using Fiji software (version 1.53 t).

## 4. Conclusions

In this work, using rational mutagenesis and in vitro screening of fluorogens libraries, we expand the color palette of the previously proposed fluorogen-activating protein nanoFAST. This protein is the smallest fluorogen-activating protein, consisting of only 98 amino acids, and until now its crucial drawback was the ability to bind only one fluorogen. Here, we discovered that E46Q was one of the key substitutions enabling the expansion of the range of possible fluorogens. This amino acid is located directly in the active center of the protein and is responsible for the formation of a hydrogen bond with the phenolic group of the fluorogen. The introduction of the substitution, as well as a number of substitutions previously proposed for the full-length fluorogen-activating protein pFAST, made it possible to use not only orange fluorogen with the modified protein but also red and green fluorogens. The identified pairs were successfully used in widefield fluorescence microscopy for labeling living cells in the GFP, TRITC, and TxRed channels.

## Figures and Tables

**Table 1:** Optical properties of **HBR-DOM2** in complex with nanoFAST and **HBR-DOM2**, **HBR-DOM,** and **HBR-2,5-DM** in complexes with nano-tFAST-E46Q.

Protein	Fluorogen	K_D_, µM ^a^	ε, M^−1^·cm^−1 b^	FQY, % ^c^	Brightness	Abs, nm ^d^	Em, nm ^e^	Lifetime, ns
nanoFAST [31]	**HBR-DOM2**	0.85 ± 0.05	25,500	56 ± 3	14,300	502	563	3.7 ^f^
nano-tFAST-E46Q	**HBR-2,5-DM**	0.50 ± 0.16	39,000 ± 590	35 ± 1.1	13,650 ± 630	511	556	2.2 ^f^
	**HBR-DOM2**	0.13 ± 0.01	47,500 ± 710	44 ± 1.8	20,850 ± 1190	512	572	3.9 ^f^
	**HBR-DOM**	1.69 ± 0.32	32,000 ± 480	42 ± 2.9	13,450 ± 1120	525	600	3.2 ^f^
EGFP [33]	-	-	55,000	60	33,000	488	509	2.6
mNeonGreen [34]	-	-	116,000	80	92,800	506	517	3.1
TagGFP [33]	-	-	58,000	59	34,220	482	505	2.6
BrUSLEE [35]	-	-	86,000	30	25,800	487	509	0.82
mOrange2 [4,36]	-	-	58,000	60	34,800	549	565	2.7
mVenus [37]	-	-	105,000	64	67,200	515	527	2.7

^a^—represented as mean ± SD (*n* = 3). ^b^—represented as result of single measurement ± the precision of the measuring instruments (weighing and pipetting errors). ^c^—fluorescence quantum yield, represented as mean ± SD (*n* = 9). ^d^—Absorbance maxima. ^e^—Emission maxima. ^f^—Mean fluorescence lifetime from biexponential fit—see Table S4.1 for more details.

## Data Availability

Data are contained within the article or Supplementary Materials.

## Supplementary Materials

The following supporting information can be downloaded at: https://www.mdpi.com/article/10.3390/ijms25053054/s1. References [40,41,42,43,44] are cited in the Supplementary Materials Part S8.
