# Supplementary material for: A Combination of Library Screening and Rational Mutagenesis Expands the Available Color Palette of the Smallest Fluorogen-Activating Protein Tag nanoFAST
Source: Int J Mol Sci. 2024 Mar 6;25(5):3054. doi: 10.3390/ijms25053054 (PMC10931682; doi:10.3390/ijms25053054)
Supplement: Supplementary file 1 [file ijms-25-03054-s001.zip › ijms-2895154-supplementary.pdf]

Contents

1. Proteins purification and sequences S2

2. Screening in vitro S3

3. Determination of affinity constants S66

4. Fluorescence Lifetime Measurements S76

5. Spectra of complexes S81

6. Fluorescent microscopy S82

7. Compounds description S83

8. References S108

9. Copies of ^{1}H and ^{13}C NMR spectra S109

1. Proteins purification and sequences

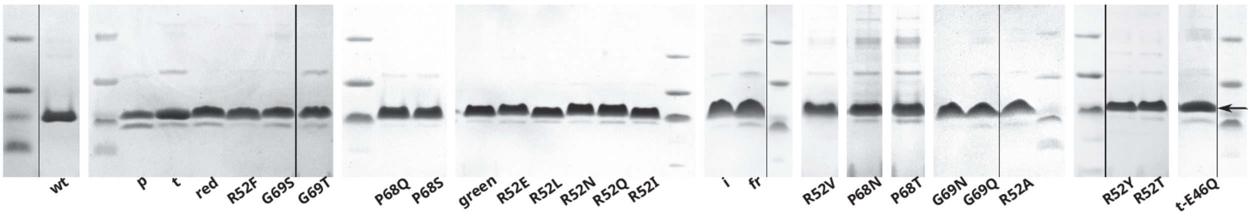

Figure S1.1. Purified nanoFAST and its mutant variants. Protein designations used are listed at **Table S1.1**. The bands corresponding to target proteins are indicated with an arrow. Black vertical separators are introduced between the lines that are not adjacent within the same gel. The full-sized raw Tris-Tricine-SDS-PAGEs for each protein line are shown at Figure S1.2. Protein molecular weight markers: 26.6 kDa, 17 kDa, 14.2 kDa, 6.5 kDa, 3.5 kDa. Molecular weight of the wild type nanoFAST (wt) is 11.9 kDa.

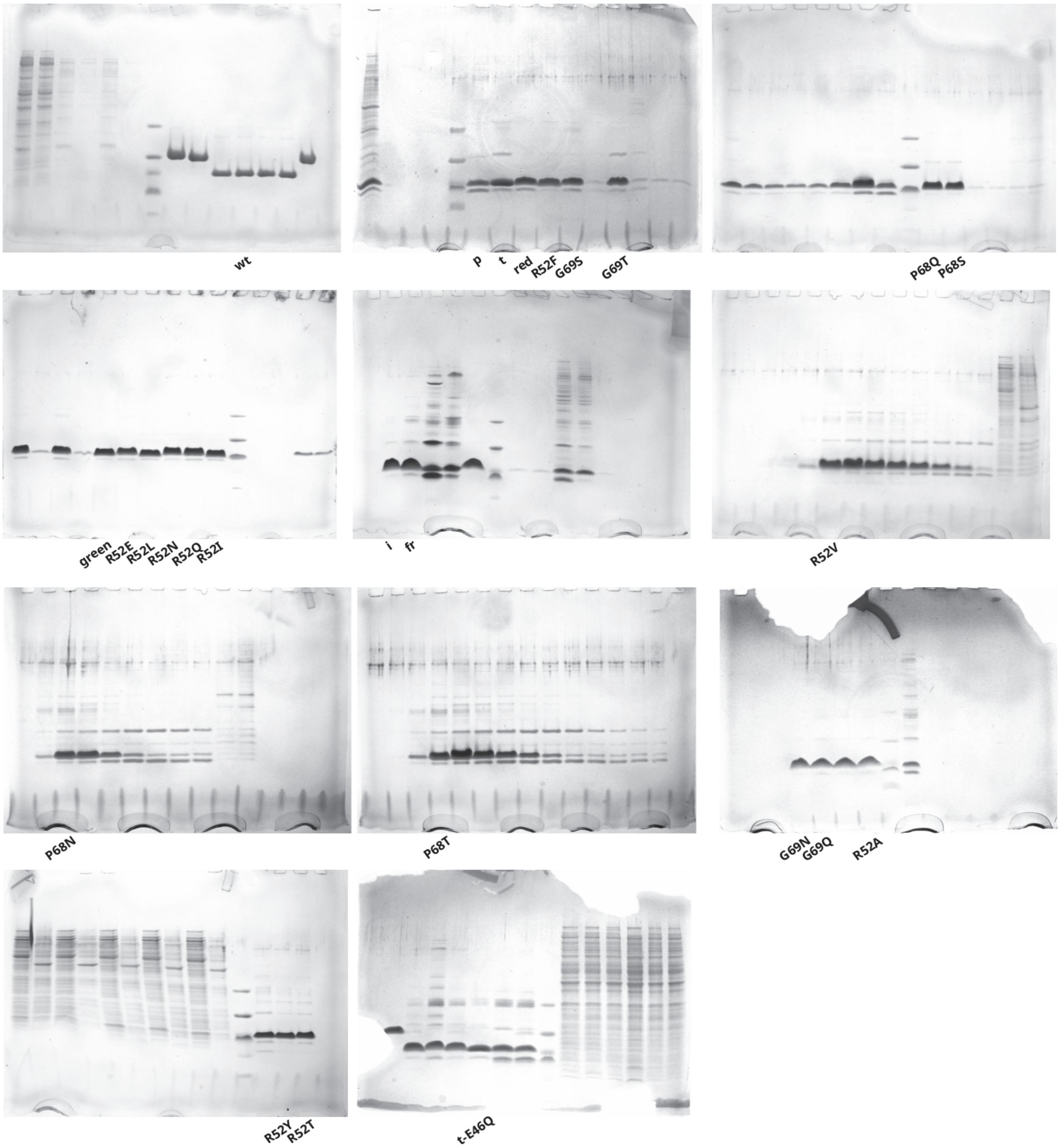

Figure S1.2. The full-sized raw Tris-Tricine-SDS-PAGEs for purified nanoFAST and its mutant variants. The protein samples shown at Fig S1.1 are signed identically (for designations and sequences see **Table S1.1)**. Protein molecular weight markers: 26.6 kDa, 17 kDa, 14.2 kDa, 6.5 kDa, 3.5 kDa. Molecular weight of the wild type nanoFAST (wt) is 11.9 kDa. The raw gels were combined with inscriptions using Inkscape 0.92 (https://www.inkscape.org).

Table S1.1 Aminoacid sequences of the synthesized mutants and yields (in mg of purified protein per liter of minimal salts medium, M9). Mutated residues are shown in bold. Here and after aminoacids numbering correspond to the parent FAST (as well as PYP) protein.

mutant	Designation (Figures S1)	Yield, mg/l M9	sequence
**nanoFAST**	**wt**	**~ 1.9**	(M)FGAIQLDGDGNILQYNAAEGDITGRDPKQVIGKNFFKD<br>VAPGTDSPEFYGKFKEGVASGNLNTMFEWMIPTSRGPTKV<br>KVHMKKALSGDSYWVFVKRV
**nano-iFAST**<br>(V107I)	**i**	**~ 2.1**	(M)FGAIQLDGDGNILQYNAAEGDITGRDPKQVIGKNFFKD<br>VAPGTDSPEFYGKFKEGVASGNLNTMFEWMIPTSRGPTKV<br>K**I**HMKKALSGDSYWVFVKRV
**nano-greenFAST**<br>(P68T, G77R)	**green**	**~ 1.6**	(M)FGAIQLDGDGNILQYNAAEGDITGRDPKQVIGKNFFKD<br>VA**T**GTDSPEFY**R**KFKEGVASGNLNTMFEWMIPTSRGPTKV<br>KVHMKKALSGDSYWVFVKRV
**nano-redFAST**<br>(F28L E46Q)	**red**	**~ 1.5**	(M)**L**GAIQLDGDGNILQYNAA**Q**GDITGADPKQVIGKNFFKD<br>VAPGTDSPEFYGKFKVGVASGNLNTMFEWMIPTNRGPTKV<br>KVHMKKALSGDSYWVFVKRV
**nano-frFAST**<br>(F62L, D71V, P73S, E74G, V107I)	**fr**	**~ 0.7**	(M)FGAIQLDGDGNILQYNAAEGDITGRDPKQVIGKN**L**FKD<br>VAPGT**V**S**SG**FYGKFKEGVASGNLNTMFEWMIPTSRGPTKV<br>K**I**HMKKALSGDSYWVFVKRV
**nano-pFAST**<br>(A30V, Q41L, S72T, V83A, M95T, M109L, S117R)	**p**	**~ 0.2**	(M)FG**V**IQLDGDGNIL**L**YNAAEGDITGRDPKQVIGKNFFKD<br>VAPGTD**T**PEFYGKFKEG**A**ASGNLNTMFEW**T**IPTSRGPTKV<br>KVH**L**KKALSGD**R**YWVFVKRV
**nano-tFAST**<br>(Q41K, S72T, A84S, M95A, M109L, S117R)	**t**	**~ 1.5**	(M)FGAIQLDGDGNIL**K**YNAAEGDITGRDPKQVIGKNFFKD<br>VAPGTD**T**PEFYGKFKEGV**S**SGNLNTMFEW**A**IPTSRGPTKV<br>KVH**L**KKALSGD**R**YWVFVKRV
**nanoFAST-R52X**<br>(X = A, E, L, V, I, Q, N, T, Y, F)	**R52X**<br>X = A<br>E<br>L<br>V<br>I<br>Q<br>N<br>T<br>Y<br>F	 **~ 1.4**<br>**~ 2.5**<br>**~ 1.8**<br>**~ 2.0**<br>**~ 1.8**<br>**~ 1.7**<br>**~ 2.5**<br>**~ 2.6**<br>**~ 2.7**<br>**~ 2.4**	(M)FGAIQLDGDGNILQYNAAEGDITG**X**DPKQVIGKNFFKD<br>VAPGTDSPEFYGKFKEGVASGNLNTMFEWMIPTSRGPTKV<br>KVHMKKALSGDSYWVFVKRV
**nanoFAST-G69X**<br>(X = S, T, N, Q)	**G69X**<br>X = S<br>T<br>N<br>Q	 **~ 3.2**<br>**~ 1.9**<br>**~ 1.4**<br>**~ 1.9**	(M)FGAIQLDGDGNILQYNAAEGDITGRDPKQVIGKNFFKD<br>VAP**X**TDSPEFYGKFKEGVASGNLNTMFEWMIPTSRGPTKV<br>KVHMKKALSGDSYWVFVKRV
**nanoFAST-P68X**<br>(X = S, T, N, Q)	**P68X**<br>X = S<br>T<br>N<br>Q	 **~ 2.1**<br>**~ 2.1**<br>**~ 1.7**<br>**~ 1.8**	(M)FGAIQLDGDGNILQYNAAEGDITGRDPKQVIGKNFFKD<br>VA**X**GTDSPEFYGKFKEGVASGNLNTMFEWMIPTSRGPTKV<br>KVHMKKALSGDSYWVFVKRV
**nano-tFAST-E46Q**<br>(Q41K, S72T, A84S, M95A, M109L, S117R, E46Q)	**t-E46Q**	**~ 1.4**	(M)FGAIQLDGDGNIL**K**YNAA**Q**GDITGRDPKQVIGKNFFKD<br>VAPGTD**T**PEFYGKFKEGV**S**SGNLNTMFEW**A**IPTSRGPTKV<br>KVH**L**KKALSGD**R**YWVFVKRV

2. Screening in vitro

Table S2.1. Structure of chromophores and their optical properties

Cmpd	Structure	Abs[a]	Em[a]	Cmpd	Structure	Abs[a]	Em[a]
**HBR-DOM2**	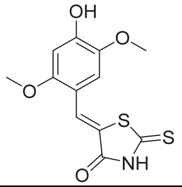	~420	~500	**M 2766**	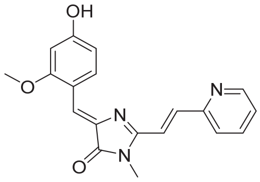	451	552
**HBR-2,5-DM**	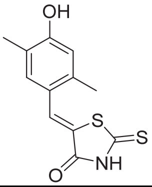	397[b]	~475[b]	**ZS 362**	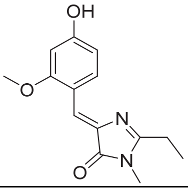	389	456
**HMBR**	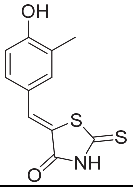	400	493	**N 1179**	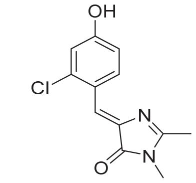	368	~400
**HBR-DOM**	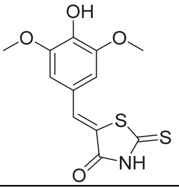	406	530	**N 1180**	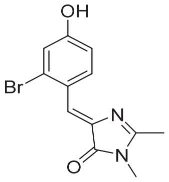	367	-[c]
**N 871b**	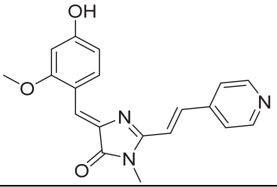	455	570	**N 1204**	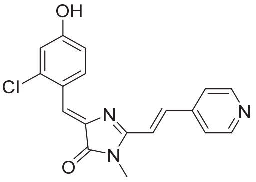	438	550
**MID 145**	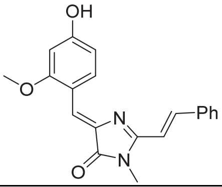	449	531	**N 1206**	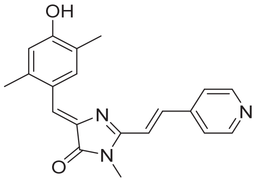	470	-[c]
**N 1052**	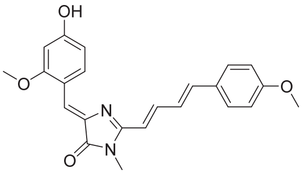	478	575	**ZS 309**	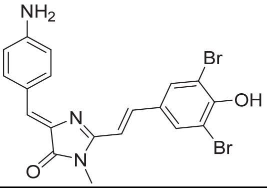	453	583
**N 1184**	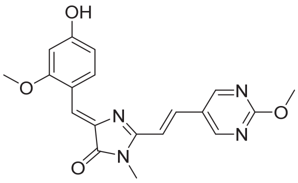	444	545	**ZS 316**	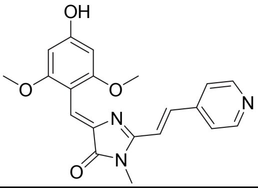	446	550
**N 1122**	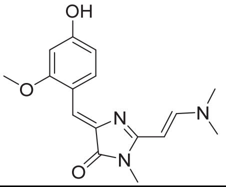	436	507	**ZS 319**	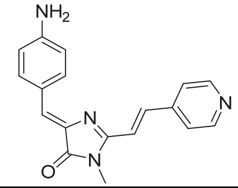	413	615

**N 1049**	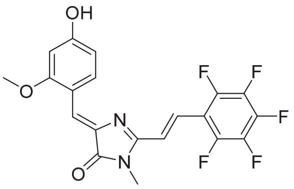	453	570	**ZS 325**	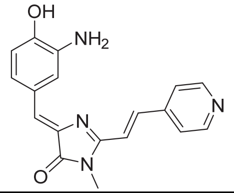	393	-[c]
**N 1123**	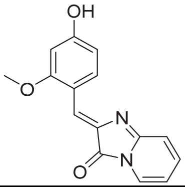	478	545	**MID 213**	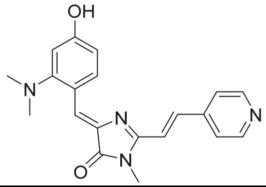	460	640
**N 1139**	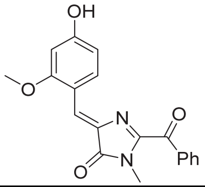	469	597	**M 2767**	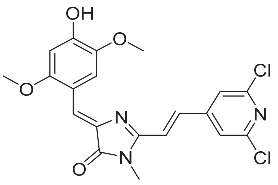	488	630
**N 1118**	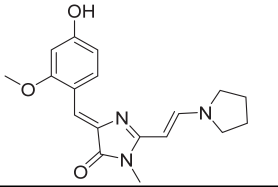	440	509	**M 2876**	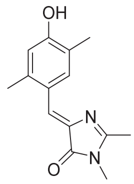	380	~400
**SAI 112**	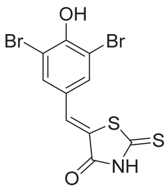	384 [b]	445 [b]	**SH 16**	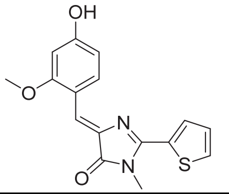	432	~510
**N 1135**	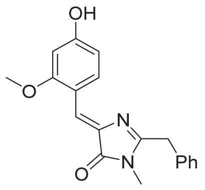	394	460	**SAI 472**	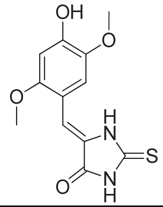	412	~550
**SAI 118**	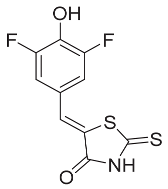	382 [b]	445 [b]	**SAI 499**	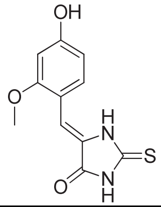	399	~500
**N 1124**	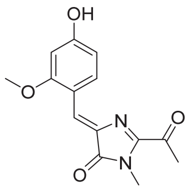	447	557	**SAI 503**	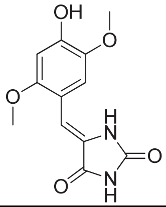	366	~500
**SAI 122**	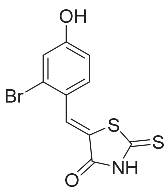	386 [b]	446 [b]	**SAI 474**	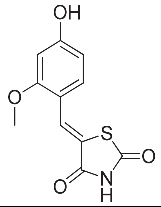	364 [b]	-[c]

**N 1142**	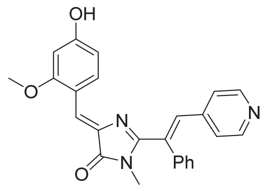	429	-[c]	**SAI 477**	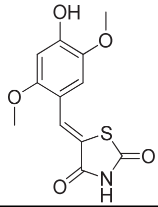	384 [b]	~480 [b]
**SAI 199**	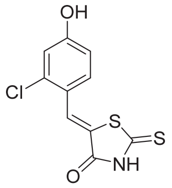	387 [b]	448 [b]	**N 1196**	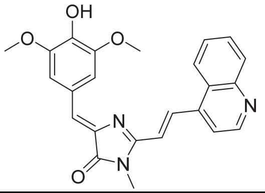	450 [b]	604 [b]
**N 967**	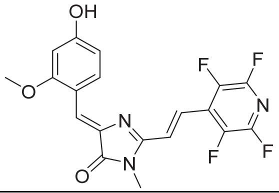	443	600	**N 1197**	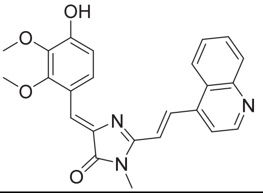	454 [b]	590 [b]
**N 1039**	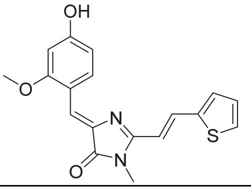	453	545	**SAI 127**	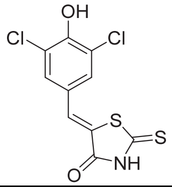	383 [b]	448 [b]
**N 1202**	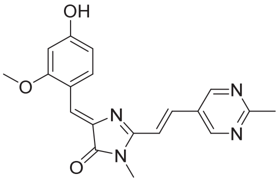	422	-[c]	**N 1056**	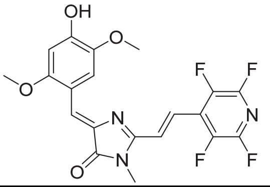	478	590
**N 960b**	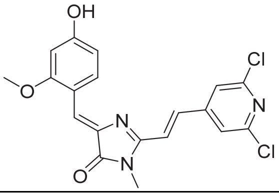	449	596	**N 1068**	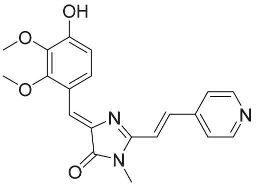	453	539
**N 1036**	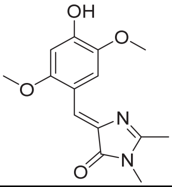	410	520	**N 1069**	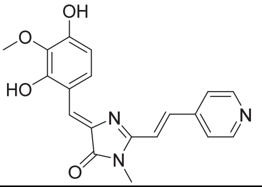	469	571
**SAI 117**	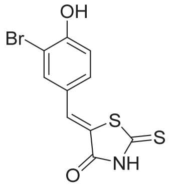	387 [b]	445 [b]	**A9**	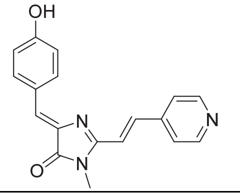	430	545
**N 1042**	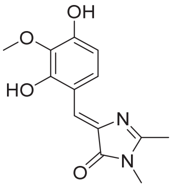	381	440	**N 1198**	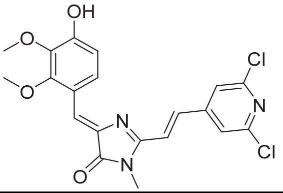	463 [b]	~580 [b]
**SAI 121**	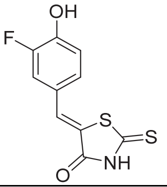	386 [b]	443 [b]	**N 1193**	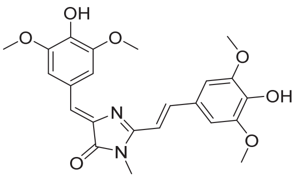	448	565

**MID 147**	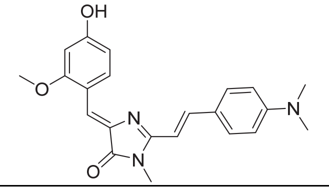	493	635	**N 1199**	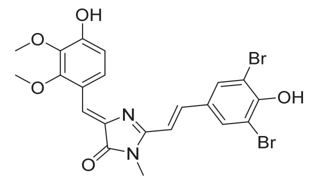	458 [b]	~560 [b]
**SAI 125**	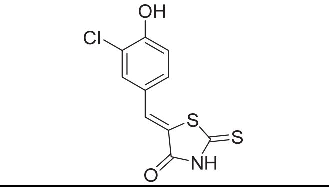	386 [b]	445 [b]	**SAI 363**	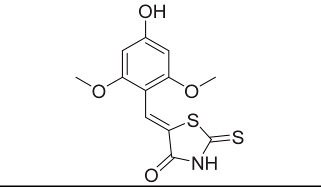	410 [b]	~470 [b]
**MID 151**	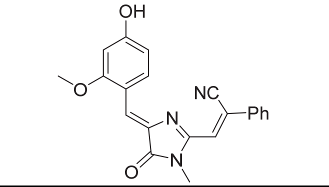	488	- [c]	**SAI 366**	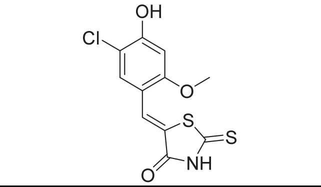	403 [b]	~480 [b]
**N 865**	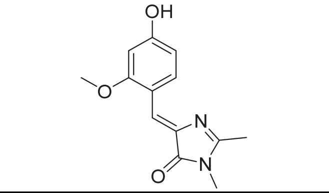	389	466	**SAI 362**	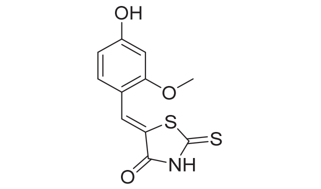	460 [b]	~475 [b]
**MID 153**	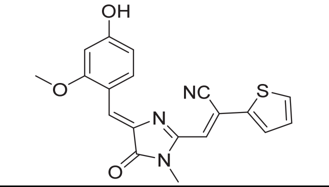	515	- [c]	**SAI 365**	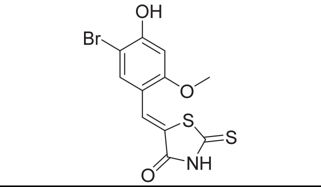	403 [b]	~480 [b]
**MID 343**	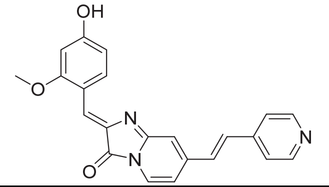	531 [d]	595 [d]	**SAI 120**	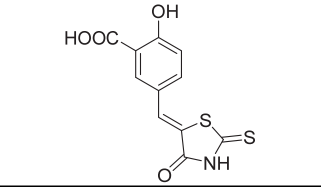	390 [b]	447 [b]
**M 3007a**	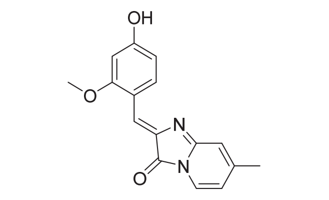	490 [d]	~550 [d]	**SAI 458**	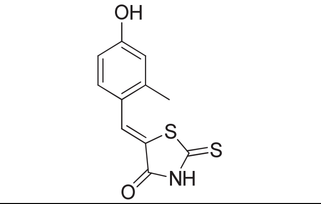	393 [b]	- [c]
**MID 367**	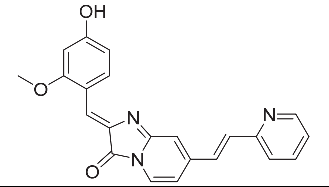	529 [d]	585 [d]	**SAI 459**	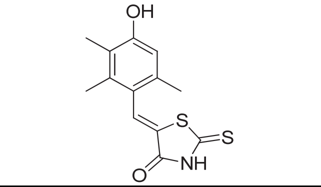	359 [b]	- [c]
**N 1048**	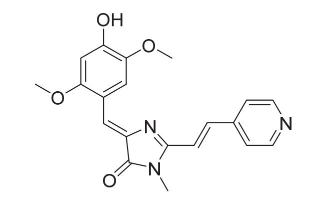	485	615	**SAI 487**	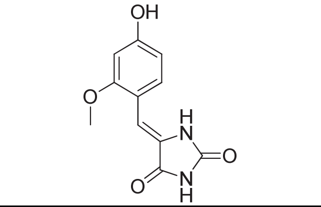	352	~450
**N 979**	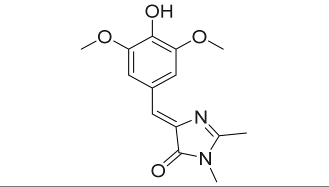	383	485	**N 1131**	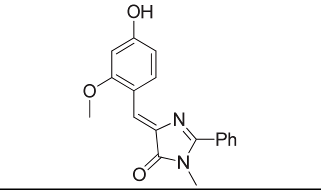	411	492

**N 971**	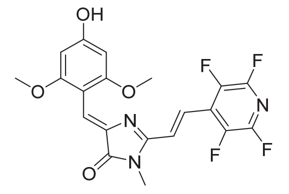	453	567	**MID 323**	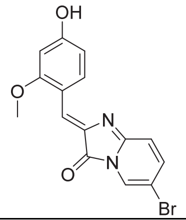	504	555
**N 973**	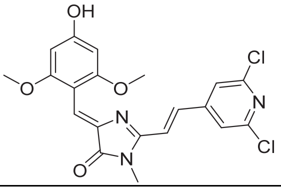	453	576	**SAI 379**	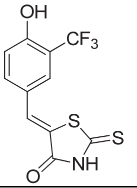	388	~500
**N 976**	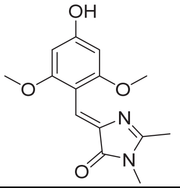	393	485	**SAI 367**	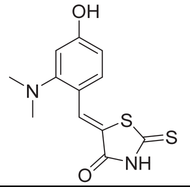	402 [b]	~570 [b]
**N 980**	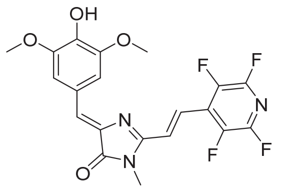	468	- [c]	**M 2738d**	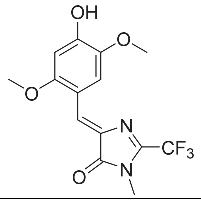	441	~550
**N 1205**	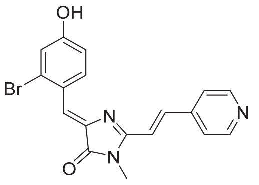	438	555	**ZS 260**	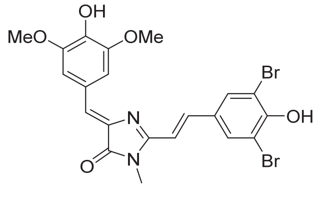	430	606
**N 960a**	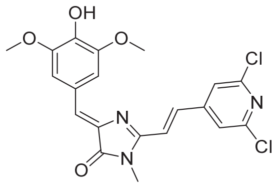	437	- [c]	**N 871a**	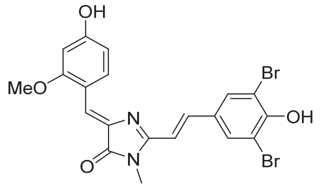	483	555
**ZS 331**	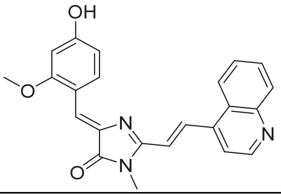	473	600	**N 901**	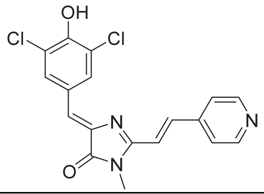	430	542
**N 1027**	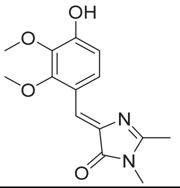	382	450				

a – maxima position in nm;
b – in acetonitrile (non soluble enough in water);
c – non fluorescent;
d – in methanol (non soluble enough in water).

Table S2.2. Results of interaction with nanoFAST

Cmpd	Enhancement			
	430	480	530	580
**HBR-DOM2**	20.5	>164.6	>265.6	3.3
**HBR-2,5-DM**	7.4	37.2	8.3	1.2
**HMBR**	2.5	5.7	1.2	1.5
**HBR-DOM**	1.3	2.9	6.4	2.3
**N 871b**	1.1	1.4	2.3	2.9
**MID 145**	1.2	1.4	1.9	0.9
**N 1052**	1.2	1.3	1.5	1.8
**N 1184**	1.2	1.6	2.4	1.8
**N 1122**	1.2	1.2	1.1	1.4
**N 1049**	1.1	1.2	1.4	1.8
**N 1123**	1.1	1.1	1.2	1.3
**N 1139**	1.1	1.2	2.6	5.2
**N 1118**	1.2	1.3	1.2	1.2
**SAI 112**	3.0	7.4	2.2	1.0
**N 1135**	1.1	1.0	1.1	1.3
**SAI 118**	1.7	2.0	1.2	1.0
**N 1124**	1.1	1.1	1.2	1.0
**SAI 122**	6.4	7.1	1.4	1.2
**N 1142**	1.1	1.1	1.2	1.1
**SAI 199**	7.3	7.5	1.1	0.9
**N 967**	1.1	1.1	1.2	1.5
**N 1039**	1.2	1.7	2.8	2.3
**N 1202**	1.2	1.5	2.5	2.5
**N 960b**	1.1	1.2	1.7	2.9
**N 1036**	1.1	1.1	1.1	1.0
**SAI 117**	2.2	2.3	1.2	1.0
**N 1042**	1.1	1.0	1.0	1.0
**SAI 121**	2.2	2.8	1.2	0.9
**MID 147**	1.5	2.2	3.4	3.2
**SAI 125**	3.6	6.1	1.2	0.9
**MID 151**	1.1	1.1	1.2	1.5
**N 865**	1.1	1.0	1.1	1.1
**MID 153**	1.1	1.1	1.2	1.4
**MID 343**	1.1	1.2	1.8	3.5
**M 3007a**	1.1	1.1	1.3	1.2
**MID 367**	1.1	1.1	1.2	1.6
**N 1048**	1.2	1.4	2.8	4.7
**N 979**	1.1	1.0	1.0	0.9
**N 971**	1.1	1.0	1.0	1.0
**N 973**	1.1	1.1	1.1	1.2
**N 976**	1.1	1.1	1.0	1.0
**N 980**	1.1	1.0	1.1	1.1
**N 1205**	1.6	2.4	3.9	3.4
**N 960a**	1.1	1.1	1.2	1.1

**ZS 331**	1.2	1.3	1.6	2.1
**N 1027**	1.1	1.0	1.0	1.0
**M 2766**	1.1	1.5	2.4	2.1
**ZS 362**	1.1	1.0	1.0	1.3
**N 1179**	1.0	1.1	1.0	1.0
**N 1180**	1.0	1.1	1.1	1.2
**N 1204**	1.3	1.9	2.7	1.8
**N 1206**	1.2	1.6	2.3	1.8
**ZS 309**	1.2	1.5	2.2	1.3
**ZS 316**	1.1	1.1	1.0	0.9
**ZS 319**	1.0	1.0	1.1	1.1
**ZS 325**	1.1	1.0	1.0	1.4
**MID 213**	1.1	1.1	1.1	1.1
**M 2767**	1.2	1.7	2.7	3.7
**M 2876**	1.1	1.0	1.0	1.3
**SH 16**	1.1	1.2	1.2	0.9
**SAI 472**	1.4	1.9	1.5	1.1
**SAI 499**	1.1	1.1	1.0	1.1
**SAI 503**	1.0	1.0	1.0	1.2
**SAI 474**	1.4	1.0	1.0	1.1
**SAI 477**	10.7	4.2	1.0	0.9
**N 1196**	1.0	1.0	1.0	1.0
**N 1197**	1.0	1.0	1.1	1.2
**SAI 127**	3.9	8.8	1.7	0.9
**N 1056**	1.0	1.0	1.2	1.5
**N 1068**	1.1	1.0	1.1	1.0
**N 1069**	1.0	1.1	1.2	1.3
**A9**	1.1	1.0	1.0	1.1
**N 1198**	1.0	1.0	1.0	0.9
**N 1193**	1.9	2.6	2.4	2.6
**N 1199**	1.1	1.3	1.7	1.2
**SAI 363**	5.0	11.2	1.2	1.0
**SAI 366**	19.8	>60.7	3.6	1.1
**SAI 362**	14.6	48.6	2.4	0.9
**SAI 365**	16.8	50.0	4.1	0.9
**SAI 120**	1.1	1.0	1.0	1.0
**SAI 458**	3.0	6.7	1.1	1.0
**SAI 459**	1.0	1.0	1.0	0.9
**SAI 487**	1.0	1.0	1.0	1.0
**N 1131**	1.1	1.1	1.0	1.1
**MID 323**	1.1	1.1	1.3	1.4
**SAI 379**	1.7	1.8	1.1	1.0
**SAI 367**	1.1	1.1	1.0	0.9
**M 2738d**	1.1	1.8	2.3	0.8
**ZS 260**	1.6	2.5	2.4	1.3
**N 871a**	1.4	2.2	3.0	2.3
**N 901**	1.0	1.1	1.3	1.4

Table S2.3. Results of interaction with nano-iFAST

Cmpd	Enhancement			
	430	480	530	580
HBR-DOM2	19.9	141.1	213.5	1.8
HBR-2,5-DM	15.6	83.0	20.0	0.9
HMBR	4.6	12.7	1.6	1.0
HBR-DOM	1.5	4.1	9.6	1.7
N 871b	1.1	1.3	1.7	1.7
MID 145	1.2	1.4	1.8	1.1
N 1052	1.2	1.4	1.7	1.6
N 1184	1.1	1.4	1.8	1.1
N 1122	1.2	1.3	1.1	1.2
N 1049	1.1	1.2	1.4	1.4
N 1123	1.1	1.1	1.1	1.1
N 1139	1.1	1.3	3.0	4.1
N 1118	1.2	1.3	1.2	1.1
SAI 112	4.7	13.2	2.3	0.9
N 1135	1.1	1.0	1.0	1.0
SAI 118	2.5	3.1	1.4	1.0
N 1124	1.1	1.1	1.2	1.1
SAI 122	7.1	7.9	1.4	1.2
N 1142	1.1	1.1	1.3	1.0
SAI 199	8.6	9.1	1.1	1.0
N 967	1.1	1.1	1.1	1.2
N 1039	1.2	1.5	2.3	1.6
N 1202	1.1	1.3	1.8	1.6
N 960b	1.1	1.2	1.7	2.3
N 1036	1.1	1.1	1.0	1.0
SAI 117	4.4	7.7	1.3	1.0
N 1042	1.1	1.0	1.0	1.0
SAI 121	3.4	4.3	1.2	1.1
MID 147	1.5	2.1	3.0	2.1
SAI 125	6.3	10.8	1.2	1.1
MID 151	1.1	1.1	1.2	1.2
N 865	1.1	1.0	1.1	1.1
MID 153	1.1	1.1	1.3	1.3
MID 343	1.1	1.2	2.4	3.9
M 3007a	1.1	1.1	1.3	1.1
MID 367	1.1	1.1	1.2	1.3
N 1048	1.1	1.2	1.7	1.9
N 979	1.1	1.0	0.9	1.0
N 971	1.1	1.0	1.1	1.1
N 973	1.0	1.1	1.1	1.2
N 976	1.1	1.1	1.0	1.0
N 980	1.1	1.0	1.1	1.1
N 1205	1.7	3.6	7.8	3.1
N 960a	1.1	1.1	1.1	1.0

**ZS 331**	1.1	1.2	1.5	1.5
**N 1027**	1.1	1.0	1.0	1.0
**M 2766**	1.1	1.3	1.9	1.6
**ZS 362**	1.1	1.0	1.0	0.9
**N 1179**	1.1	1.1	1.1	1.0
**N 1180**	1.1	1.0	1.1	0.9
**N 1204**	1.5	3.0	6.1	2.8
**N 1206**	1.0	1.1	1.3	1.3
**ZS 309**	1.2	1.8	3.1	2.3
**ZS 316**	1.1	1.0	1.0	1.0
**ZS 319**	1.0	1.0	1.0	1.1
**ZS 325**	1.1	1.0	1.1	1.0
**MID 213**	1.1	1.1	1.1	1.1
**M 2767**	1.1	1.2	1.4	1.7
**M 2876**	1.1	1.1	1.0	1.0
**SH 16**	1.1	1.1	1.2	1.0
**SAI 472**	1.2	1.0	1.0	1.0
**SAI 499**	1.2	1.4	1.2	1.2
**SAI 503**	1.0	1.0	1.0	1.1
**SAI 474**	1.1	1.0	1.0	0.9
**SAI 477**	1.3	1.0	1.0	1.0
**N 1196**	5.2	2.5	1.0	1.1
**N 1197**	1.0	1.0	1.1	1.1
**SAI 127**	1.1	1.0	1.1	1.1
**N 1056**	1.0	1.0	1.1	1.2
**N 1068**	1.0	1.0	1.1	1.1
**N 1069**	1.0	1.2	1.3	1.1
**A9**	1.0	1.0	1.0	1.0
**N 1198**	1.0	1.0	1.1	1.1
**N 1193**	1.7	2.4	2.4	2.6
**N 1199**	1.1	1.4	1.9	1.3
**SAI 363**	4.2	10.4	1.2	1.1
**SAI 366**	25.6	>79.6	4.4	1.0
**SAI 362**	11.5	41.3	2.4	1.0
**SAI 365**	25.0	>75.1	5.6	1.0
**SAI 120**	1.1	1.1	1.1	1.0
**SAI 458**	5.0	14.6	1.3	1.0
**SAI 459**	1.0	1.0	1.0	1.1
**SAI 487**	1.0	1.0	1.0	0.9
**N 1131**	1.1	1.1	1.1	1.0
**MID 323**	1.1	1.1	1.4	1.8
**SAI 379**	2.3	2.3	1.1	1.1
**SAI 367**	1.5	1.3	1.1	1.1
**M 2738d**	1.1	1.5	2.0	1.2
**ZS 260**	2.0	3.7	4.3	1.8
**N 871a**	1.7	3.1	4.9	2.7
**N 901**	1.1	1.2	1.5	1.9

Table S2.4. Results of interaction with nano-frFAST

Cmpd	Enhancement			
	430	480	530	580
**HBR-DOM2**	2.9	15.9	22.9	1.6
**HBR-2,5-DM**	3.0	12.8	5.4	1.2
**HMBR**	2.6	5.8	1.8	1.3
**HBR-DOM**	4.3	28.2	63.4	15.1
**N 871b**	1.2	1.5	2.0	2.6
**MID 145**	1.3	2.1	4.5	2.0
**N 1052**	1.7	3.4	8.3	14.1
**N 1184**	1.2	1.4	1.7	2.6
**N 1122**	1.2	1.5	1.5	1.3
**N 1049**	1.3	2.0	5.7	13.0
**N 1123**	1.2	1.2	1.4	1.6
**N 1139**	1.5	7.4	39.7	51.1
**N 1118**	1.3	1.6	2.5	2.4
**SAI 112**	2.7	7.0	3.5	1.6
**N 1135**	1.2	1.2	1.8	3.0
**SAI 118**	1.5	1.8	1.5	1.6
**N 1124**	1.4	3.7	7.2	4.6
**SAI 122**	2.4	2.8	1.5	1.5
**N 1142**	2.3	9.9	28.3	19.5
**SAI 199**	2.8	3.0	1.2	1.2
**N 967**	1.3	2.2	8.0	26.5
**N 1039**	1.6	3.7	8.9	7.4
**N 1202**	1.2	1.3	1.6	2.3
**N 960b**	1.2	1.9	5.9	15.9
**N 1036**	1.1	1.2	1.2	1.1
**SAI 117**	2.0	3.3	1.4	1.0
**N 1042**	1.2	1.1	1.2	1.3
**SAI 121**	1.9	2.4	1.5	1.3
**MID 147**	2.6	6.2	12.4	17.4
**SAI 125**	2.0	3.1	1.3	1.5
**MID 151**	1.3	1.9	5.9	12.3
**N 865**	1.2	1.1	1.2	1.3
**MID 153**	1.3	1.5	4.9	15.1
**MID 343**	1.2	1.5	3.5	12.1
**M 3007a**	1.2	1.3	1.6	2.3
**MID 367**	1.2	1.2	1.7	4.6
**N 1048**	1.5	2.7	10.2	18.8
**N 979**	1.2	1.2	1.2	1.6
**N 971**	1.2	1.3	2.6	6.7
**N 973**	1.3	1.4	2.1	2.9
**N 976**	1.2	1.1	1.2	1.3
**N 980**	1.1	1.1	1.4	4.6
**N 1205**	1.8	3.4	5.4	5.5
**N 960a**	1.2	1.2	2.8	18.9

**ZS 331**	1.4	2.5	6.5	12.3
**N 1027**	1.2	1.1	1.1	1.1
**M 2766**	1.3	1.8	2.9	4.2
**ZS 362**	1.2	1.1	1.2	1.2
**N 1179**	1.2	1.2	1.3	1.1
**N 1180**	1.2	1.2	1.3	1.2
**N 1204**	1.6	2.7	3.9	2.4
**N 1206**	1.2	1.5	2.2	2.2
**ZS 309**	2.5	6.3	9.8	2.6
**ZS 316**	1.2	1.2	1.2	1.3
**ZS 319**	1.2	1.1	1.2	1.0
**ZS 325**	1.2	1.2	1.5	1.5
**MID 213**	1.4	2.4	5.0	4.2
**M 2767**	1.6	2.2	12.5	42.6
**M 2876**	1.2	1.1	1.2	1.0
**SH 16**	2.0	7.2	7.1	1.2
**SAI 472**	1.5	2.5	1.8	0.9
**SAI 499**	1.2	1.1	1.1	1.1
**SAI 503**	1.3	1.3	1.1	1.1
**SAI 474**	1.3	1.1	1.1	1.0
**SAI 477**	3.4	1.8	1.2	1.1
**N 1196**	1.3	1.4	2.6	5.4
**N 1197**	1.2	1.2	1.2	1.2
**SAI 127**	2.4	5.0	2.0	1.1
**N 1056**	1.2	1.2	1.8	3.8
**N 1068**	1.2	1.1	1.2	1.1
**N 1069**	1.2	1.3	1.4	1.3
**A9**	1.2	1.2	1.2	1.1
**N 1198**	1.2	1.1	1.3	1.2
**N 1193**	2.9	5.5	21.5	31.6
**N 1199**	2.2	6.0	10.6	2.1
**SAI 363**	2.0	4.6	1.4	0.9
**SAI 366**	4.2	9.8	2.2	1.2
**SAI 362**	2.4	5.2	1.5	1.3
**SAI 365**	3.9	8.7	2.7	1.1
**SAI 120**	1.3	1.3	1.4	1.2
**SAI 458**	1.8	2.9	1.3	1.1
**SAI 459**	1.2	1.2	1.2	1.0
**SAI 487**	1.2	1.1	1.2	1.2
**N 1131**	1.9	4.4	1.7	1.1
**MID 323**	1.2	1.3	2.6	4.2
**SAI 379**	2.1	2.8	1.4	1.1
**SAI 367**	2.9	5.1	2.6	1.3
**M 2738d**	1.6	7.4	13.1	1.1
**ZS 260**	3.0	7.3	10.3	6.4
**N 871a**	3.0	7.0	8.6	2.9
**N 901**	1.7	2.9	4.3	4.7

Table S2.5. Results of interaction with nano-pFAST

Cmpd	Enhancement			
	430	480	530	580
**HBR-DOM2**	28.0	>206.2	>386.1	3.2
**N 1036**	1.5	3.7	1.5	1.1
**N 1048**	1.7	4.6	24.4	43.0
**SAI 472**	5.8	16.5	9.5	1.0
**SAI 503**	1.3	1.1	1.0	1.0
**SAI 477**	39.4	16.6	1.1	1.0
**N 1056**	1.1	1.1	1.7	2.7
**M 2738d**	1.9	18.4	37.2	1.1

Table S2.6. Results of interaction with nano-tFAST

Cmpd	Enhancement			
	430	480	530	580
**HBR-DOM2**	30.2	>235.0	>365.8	2.5
**HBR-2,5-DM**	27.0	>166.8	35.7	1.1
**HMBR**	8.4	24.4	2.1	0.9
**HBR-DOM**	2.1	12.9	26.6	4.0
**N 871b**	1.5	4.8	9.3	9.0
**MID 145**	1.6	4.2	6.9	1.3
**N 1052**	1.3	2.5	4.6	3.4
**N 1184**	1.4	4.5	8.8	3.0
**N 1122**	1.3	1.5	1.3	1.2
**N 1049**	1.2	1.9	3.1	1.9
**N 1123**	1.1	1.2	1.7	1.8
**N 1139**	1.1	2.0	6.2	10.6
**N 1118**	1.4	1.9	1.7	1.2
**SAI 112**	6.8	17.0	3.3	0.9
**N 1135**	1.1	1.1	1.2	1.3
**SAI 118**	4.9	5.3	1.5	0.9
**N 1124**	1.1	1.2	1.4	1.6
**SAI 122**	8.7	6.6	1.5	1.2
**N 1142**	1.2	1.6	2.1	1.3
**SAI 199**	10.8	8.0	1.1	1.1
**N 967**	1.0	1.3	2.1	3.0
**N 1039**	2.0	6.1	12.5	5.6
**N 1202**	1.5	4.8	9.9	7.9
**N 960b**	1.2	2.0	4.2	7.2
**N 1036**	1.1	1.3	1.1	1.1
**SAI 117**	7.8	10.6	1.4	0.9
**N 1042**	1.0	1.0	1.1	1.0
**SAI 121**	6.6	6.1	1.3	1.0
**MID 147**	2.7	8.1	13.5	8.8
**SAI 125**	10.4	13.0	1.3	1.1
**MID 151**	1.1	1.1	1.4	1.9
**N 865**	1.0	1.0	1.0	1.1
**MID 153**	1.1	1.1	1.3	1.6
**MID 343**	1.1	1.4	3.5	8.2
**M 3007a**	1.1	1.3	2.3	2.4
**MID 367**	1.1	1.1	1.6	2.4
**N 1048**	1.3	2.8	8.6	14.6
**N 979**	1.0	1.0	1.1	1.1
**N 971**	1.0	1.0	1.0	1.1
**N 973**	1.1	1.1	1.3	1.5
**N 976**	1.1	1.0	1.0	1.2
**N 980**	1.1	1.1	1.1	1.3
**N 1205**	2.6	7.4	13.4	6.7
**N 960a**	1.1	1.3	1.4	1.2

**ZS 331**	1.5	2.8	4.6	4.7
**N 1027**	1.0	1.0	1.1	0.9
**M 2766**	1.6	4.6	8.8	5.6
**ZS 362**	1.0	1.0	1.0	1.1
**N 1179**	1.1	1.1	1.1	1.2
**N 1180**	1.1	1.1	1.1	1.2
**N 1204**	2.0	5.0	9.0	4.1
**N 1206**	1.1	1.1	1.6	2.3
**ZS 309**	1.7	3.0	5.2	4.2
**ZS 316**	1.1	1.0	1.0	1.0
**ZS 319**	1.0	1.0	1.0	1.0
**ZS 325**	1.1	1.0	1.1	1.0
**MID 213**	1.1	1.2	1.5	1.7
**M 2767**	1.7	4.6	12.0	16.0
**M 2876**	1.1	1.0	1.0	1.1
**SH 16**	1.4	2.2	2.5	1.1
**SAI 472**	3.6	7.2	3.2	1.1
**SAI 499**	1.5	1.3	1.0	1.0
**SAI 503**	1.2	1.1	1.0	1.0
**SAI 474**	2.8	1.0	1.1	1.0
**SAI 477**	30.2	10.4	1.0	1.0
**N 1196**	1.1	1.1	1.2	1.1
**N 1197**	1.1	1.1	1.1	1.1
**SAI 127**	7.6	20.2	1.8	1.0
**N 1056**	1.1	1.1	1.6	2.4
**N 1068**	1.1	1.1	1.1	1.2
**N 1069**	1.0	1.3	1.3	1.0
**A9**	1.1	1.1	1.0	0.9
**N 1198**	1.1	1.1	1.1	1.1
**N 1193**	4.1	6.6	4.9	6.2
**N 1199**	1.2	1.7	2.8	2.0
**SAI 363**	14.4	33.7	1.4	1.1
**SAI 366**	23.0	>67.5	3.8	1.0
**SAI 362**	24.7	82.1	3.0	0.9
**SAI 365**	20.1	>60.3	4.6	1.0
**SAI 120**	1.2	1.2	1.3	1.2
**SAI 458**	10.9	31.0	1.7	1.0
**SAI 459**	1.1	1.0	1.0	0.9
**SAI 487**	1.0	1.0	1.0	1.1
**N 1131**	1.2	1.4	1.2	0.8
**MID 323**	1.1	1.2	2.3	4.2
**SAI 379**	3.3	3.1	1.1	0.9
**SAI 367**	2.4	2.2	1.3	1.1
**M 2738d**	1.4	5.6	10.2	0.9
**ZS 260**	2.8	6.0	6.5	2.4
**N 871a**	2.9	7.7	14.8	10.4
**N 901**	1.1	1.3	1.9	2.7

Table S2.7. Results of interaction with nano-greenFAST

Cmpd	Enhancement			
	430	480	530	580
**HBR-DOM2**	2.1	7.2	8.8	1.1
**HBR-2,5-DM**	1.7	2.5	1.4	1.0
**HMBR**	3.6	7.4	1.2	1.0
**HBR-DOM**	1.1	1.2	1.5	1.1
**N 871b**	1.0	1.0	1.0	1.0
**MID 145**	1.2	1.4	1.2	1.1
**N 1052**	1.2	1.8	2.4	1.9
**N 1184**	1.0	1.0	1.1	1.2
**N 1122**	1.0	1.1	1.1	1.0
**N 1049**	1.1	1.3	1.6	1.2
**N 1123**	1.0	1.0	1.1	1.1
**N 1139**	1.0	1.1	1.7	2.1
**N 1118**	1.1	1.1	1.1	1.1
**SAI 112**	2.2	5.2	2.9	1.0
**N 1135**	1.0	1.0	1.1	1.1
**SAI 118**	1.5	1.8	1.0	1.1
**N 1124**	1.0	1.0	1.0	1.1
**SAI 122**	1.3	1.4	1.3	1.1
**N 1142**	1.1	1.3	1.4	1.3
**SAI 199**	1.2	1.1	1.0	1.1
**N 967**	1.0	1.0	1.1	1.2
**N 1039**	1.2	1.4	1.5	1.2
**N 1202**	1.0	0.9	1.0	1.1
**N 960b**	1.0	1.1	1.2	1.3
**N 1036**	1.0	0.9	0.9	1.0
**SAI 117**	3.1	4.5	1.2	1.0
**N 1042**	1.0	1.0	1.0	1.1
**SAI 121**	1.5	1.8	1.2	1.0
**MID 147**	1.9	4.2	6.1	2.2
**SAI 125**	2.9	3.9	1.1	1.1
**MID 151**	1.0	0.9	1.0	1.0
**N 865**	1.0	1.0	1.0	1.2
**MID 153**	1.0	1.0	1.0	0.9
**MID 343**	1.1	1.2	2.2	4.3
**M 3007a**	1.0	1.1	1.2	0.8
**MID 367**	1.0	1.0	1.1	1.1
**N 1048**	1.0	1.0	1.1	1.1
**N 979**	1.0	1.0	1.0	1.2
**N 971**	1.0	1.0	0.9	0.9
**N 973**	1.0	1.0	1.1	1.1
**N 976**	1.0	1.0	1.0	0.8
**N 980**	1.0	1.0	1.1	1.1
**N 1205**	1.2	1.3	1.4	1.5
**N 960a**	1.0	1.0	1.0	1.3

**ZS 331**	1.1	1.3	1.4	1.3
**N 1027**	1.0	1.0	1.0	0.9
**M 2766**	1.0	1.0	1.0	1.1
**ZS 362**	1.0	1.0	1.0	1.0
**N 1179**	1.0	1.0	1.0	1.0
**N 1180**	1.0	1.0	1.0	1.1
**N 1204**	1.1	1.2	1.2	1.6
**N 1206**	1.0	1.1	1.1	1.3
**ZS 309**	1.3	1.8	2.4	2.0
**ZS 316**	1.0	1.0	1.0	0.8
**ZS 319**	1.0	0.9	0.9	0.9
**ZS 325**	1.0	1.0	1.0	1.1
**MID 213**	1.0	1.0	1.2	1.1
**M 2767**	1.1	1.3	1.9	2.3
**M 2876**	1.0	1.0	1.0	1.0
**SH 16**	1.1	1.0	1.1	0.9
**SAI 472**	1.1	1.1	1.0	1.0
**SAI 499**	1.0	1.0	1.0	1.0
**SAI 503**	1.0	1.0	1.0	1.1
**SAI 474**	1.0	1.0	1.0	0.9
**SAI 477**	1.2	1.0	1.0	1.1
**N 1196**	1.0	1.0	1.1	0.9
**N 1197**	1.0	1.0	1.0	1.1
**SAI 127**	1.8	3.9	2.0	0.9
**N 1056**	1.0	1.0	1.1	1.1
**N 1068**	1.0	1.0	1.1	1.1
**N 1069**	1.0	1.2	1.3	1.0
**A9**	1.0	1.0	0.9	1.1
**N 1198**	1.0	1.0	1.0	1.1
**N 1193**	3.6	6.1	2.8	2.6
**N 1199**	1.1	1.3	1.9	1.2
**SAI 363**	1.2	1.7	1.2	1.1
**SAI 366**	3.8	7.6	2.0	0.9
**SAI 362**	1.3	1.8	1.1	1.2
**SAI 365**	3.3	6.8	2.7	1.0
**SAI 120**	1.1	1.1	1.1	1.0
**SAI 458**	1.0	1.1	1.0	1.0
**SAI 459**	1.0	1.0	1.0	1.0
**SAI 487**	1.0	1.0	1.0	1.0
**N 1131**	1.0	1.0	1.0	0.9
**MID 323**	1.0	1.1	1.5	1.5
**SAI 379**	1.4	1.8	1.1	1.1
**SAI 367**	1.5	1.3	1.1	1.1
**M 2738d**	1.0	1.5	2.1	1.0
**ZS 260**	1.7	3.0	2.8	1.4
**N 871a**	1.9	3.4	3.6	1.8
**N 901**	1.0	1.0	1.2	1.5

Table S2.8. Results of interaction with nano-redFAST

Cmpd	Enhancement			
	430	480	530	580
**HBR-DOM2**	14.5	>151.7	>400.5	15.4
**HBR-2,5-DM**	14.0	>153.1	194.4	1.3
**HMBR**	9.1	54.8	18.3	0.9
**HBR-DOM**	4.4	45.0	204.5	69.3
**N 871b**	1.1	1.9	5.1	12.2
**MID 145**	1.2	2.1	4.8	1.7
**N 1052**	1.1	1.5	2.7	2.9
**N 1184**	1.1	1.7	4.7	5.2
**N 1122**	1.1	1.2	1.2	0.9
**N 1049**	1.1	1.5	3.2	3.3
**N 1123**	1.0	1.0	1.3	1.6
**N 1139**	1.0	1.2	2.7	4.5
**N 1118**	1.1	1.4	1.3	1.1
**SAI 112**	11.9	>65.1	5.1	1.0
**N 1135**	1.0	1.0	1.1	1.2
**SAI 118**	13.0	49.5	3.2	1.0
**N 1124**	1.0	1.0	1.2	1.7
**SAI 122**	11.0	36.7	1.9	1.2
**N 1142**	1.1	1.4	2.0	1.5
**SAI 199**	15.3	51.8	1.8	0.9
**N 967**	1.0	1.1	1.5	2.4
**N 1039**	1.3	2.4	7.6	7.5
**N 1202**	1.0	1.8	5.6	12.0
**N 960b**	1.0	1.1	2.0	3.8
**N 1036**	1.0	1.2	1.1	1.1
**SAI 117**	13.5	52.0	3.0	1.0
**N 1042**	1.0	1.0	1.0	1.0
**SAI 121**	9.9	38.8	2.4	0.9
**MID 147**	1.9	3.7	8.3	10.8
**SAI 125**	16.5	66.5	4.0	1.1
**MID 151**	1.0	1.0	1.0	1.2
**N 865**	1.0	1.0	1.0	1.0
**MID 153**	1.0	1.0	1.0	1.2
**MID 343**	1.0	1.0	1.7	4.1
**M 3007a**	1.0	1.0	1.4	1.8
**MID 367**	1.0	1.0	1.2	1.7
**N 1048**	1.1	1.4	4.6	10.7
**N 979**	1.0	0.9	1.0	1.4
**N 971**	1.0	1.0	1.0	1.1
**N 973**	1.0	1.0	1.1	1.2
**N 976**	1.0	1.0	1.0	1.0
**N 980**	1.0	1.0	1.1	1.1
**N 1205**	1.9	7.4	24.7	29.7
**N 960a**	1.0	1.0	1.1	1.2

**ZS 331**	1.1	1.3	2.1	3.7
**N 1027**	1.0	1.0	1.0	1.0
**M 2766**	1.1	2.0	5.5	9.8
**ZS 362**	1.0	1.0	1.0	1.1
**N 1179**	1.1	1.1	1.0	1.1
**N 1180**	1.0	1.0	1.1	1.3
**N 1204**	1.6	5.4	17.4	17.1
**N 1206**	1.0	1.0	1.6	2.8
**ZS 309**	1.3	1.8	3.0	2.7
**ZS 316**	1.0	1.0	1.0	1.0
**ZS 319**	1.0	0.9	1.2	1.0
**ZS 325**	1.0	1.0	1.0	0.9
**MID 213**	1.0	1.1	1.1	1.5
**M 2767**	1.3	2.0	3.1	7.1
**M 2876**	1.0	1.0	1.1	1.1
**SH 16**	1.1	1.9	2.6	1.2
**SAI 472**	1.6	3.5	2.2	0.9
**SAI 499**	1.1	1.2	1.0	1.1
**SAI 503**	1.0	1.0	1.1	0.9
**SAI 474**	2.4	1.0	1.1	1.1
**SAI 477**	16.7	11.5	3.9	3.4
**N 1196**	1.0	1.0	1.0	0.9
**N 1197**	1.0	1.0	1.1	1.0
**SAI 127**	13.6	>69.8	3.9	1.2
**N 1056**	1.0	1.0	1.3	1.9
**N 1068**	1.0	1.0	1.0	0.9
**N 1069**	1.0	1.1	1.0	1.0
**A9**	1.0	1.0	1.0	1.0
**N 1198**	1.0	1.0	1.0	1.0
**N 1193**	1.6	2.2	2.4	2.9
**N 1199**	1.1	1.5	2.7	2.3
**SAI 363**	7.7	35.4	5.3	2.1
**SAI 366**	19.2	>89.0	7.4	3.3
**SAI 362**	15.8	>105.4	6.9	2.1
**SAI 365**	18.3	>86.8	3.7	2.0
**SAI 120**	1.1	1.1	1.2	1.0
**SAI 458**	5.7	28.7	8.1	1.5
**SAI 459**	1.0	1.0	1.1	1.2
**SAI 487**	1.0	1.0	1.1	1.1
**N 1131**	1.0	1.3	1.1	1.0
**MID 323**	1.0	1.1	1.6	2.4
**SAI 379**	7.1	13.1	1.2	1.0
**SAI 367**	1.3	1.4	1.4	0.9
**M 2738d**	1.1	3.2	5.7	1.0
**ZS 260**	1.5	2.4	2.9	2.0
**N 871a**	1.8	3.3	6.9	10.4
**N 901**	2.5	8.4	20.8	26.3

Table S2.9. Results of interaction with nanoFAST-R52F

Cmpd	Enhancement			
	430	480	530	580
**HBR-DOM2**	4.0	27.4	59.3	1.5
**HBR-2,5-DM**	1.1	1.5	1.2	0.9
**HMBR**	1.1	1.3	1.1	1.0
**HBR-DOM**	1.0	1.1	1.3	1.2
**N 871b**	1.0	1.1	1.3	1.2
**MID 145**	1.2	1.3	1.2	1.1
**N 1052**	1.1	1.4	1.7	1.2
**N 1184**	1.0	1.1	1.2	1.1
**N 1122**	1.1	1.2	1.2	1.2
**N 1049**	1.1	1.3	1.4	1.1
**N 1123**	1.0	1.0	1.0	1.1
**N 1139**	1.0	1.1	1.7	2.3
**N 1118**	1.1	1.3	1.1	1.0
**SAI 112**	1.3	1.9	1.5	1.0
**N 1135**	1.0	1.0	1.1	1.2
**SAI 118**	0.9	1.0	1.0	1.1
**N 1124**	1.0	1.0	1.0	1.2
**SAI 122**	3.9	4.3	1.2	1.0
**N 1142**	1.1	1.2	1.3	1.0
**SAI 199**	5.2	5.8	1.0	1.0
**N 967**	1.0	1.0	1.4	1.7
**N 1039**	1.1	1.3	1.4	1.2
**N 1202**	1.0	1.0	1.2	1.1
**N 960b**	1.0	1.1	1.5	1.7
**N 1036**	1.0	1.0	1.1	1.1
**SAI 117**	1.1	1.3	1.1	1.0
**N 1042**	1.0	1.0	1.0	1.0
**SAI 121**	1.0	1.1	1.1	0.9
**MID 147**	1.6	2.8	3.8	1.9
**SAI 125**	1.1	1.2	1.1	0.9
**MID 151**	1.0	0.9	1.0	1.0
**N 865**	1.0	1.0	1.0	1.0
**MID 153**	1.0	0.9	0.9	0.9
**MID 343**	1.0	1.0	1.3	1.4
**M 3007a**	1.0	1.0	1.1	1.0
**MID 367**	1.0	1.0	1.1	1.1
**N 1048**	1.0	1.1	1.0	0.8
**N 979**	1.0	0.9	1.0	1.0
**N 971**	1.0	1.0	0.9	0.9
**N 973**	1.0	1.1	1.1	1.0
**N 976**	1.0	1.0	1.0	1.1
**N 980**	1.0	1.0	1.0	0.9
**N 1205**	1.3	1.5	1.8	1.5
**N 960a**	1.0	1.1	1.2	1.1

**ZS 331**	1.1	1.1	1.2	1.1
**N 1027**	1.0	1.0	1.0	1.0
**M 2766**	1.0	1.1	1.2	1.1
**ZS 362**	1.0	1.0	1.0	1.0
**N 1179**	1.0	1.0	1.1	1.0
**N 1180**	1.0	1.0	1.1	0.9
**N 1204**	1.1	1.3	1.5	1.5
**N 1206**	1.0	1.0	1.0	0.9
**ZS 309**	1.2	1.4	1.9	1.7
**ZS 316**	1.0	1.0	1.0	1.2
**ZS 319**	1.0	1.0	1.0	0.8
**ZS 325**	1.0	1.0	1.0	1.2
**MID 213**	1.0	1.0	1.1	1.0
**M 2767**	1.2	1.8	2.8	3.8
**M 2876**	1.0	1.0	1.0	0.9
**SH 16**	1.0	1.0	1.0	1.0
**SAI 472**	1.1	1.2	1.1	0.9
**SAI 499**	1.0	1.0	1.0	1.2
**SAI 503**	1.0	1.0	1.0	0.8
**SAI 474**	1.0	1.0	1.0	1.2
**SAI 477**	1.3	1.2	1.0	1.1
**N 1196**	1.0	1.0	1.0	1.2
**N 1197**	1.0	1.0	1.0	1.3
**SAI 127**	1.1	1.6	1.2	1.1
**N 1056**	1.0	1.0	1.1	1.2
**N 1068**	1.0	1.0	1.1	1.0
**N 1069**	1.0	1.0	1.1	1.1
**A9**	1.0	1.0	1.0	1.0
**N 1198**	1.0	1.0	1.0	1.0
**N 1193**	1.4	1.8	2.0	2.3
**N 1199**	1.0	1.1	1.4	1.3
**SAI 363**	1.4	2.5	1.1	1.0
**SAI 366**	3.7	10.0	2.4	1.0
**SAI 362**	3.7	11.2	1.3	1.0
**SAI 365**	3.5	9.3	3.1	0.8
**SAI 120**	1.1	1.1	1.2	1.2
**SAI 458**	1.3	2.0	1.1	1.0
**SAI 459**	1.0	1.0	1.0	1.0
**SAI 487**	1.0	1.0	1.0	1.0
**N 1131**	1.0	1.0	1.0	0.9
**MID 323**	1.0	1.0	1.2	1.7
**SAI 379**	0.9	1.1	1.0	1.1
**SAI 367**	1.5	1.2	1.1	1.0
**M 2738d**	1.0	1.2	1.6	0.9
**ZS 260**	1.4	2.1	2.1	1.3
**N 871a**	1.4	2.2	2.5	1.8
**N 901**	1.0	1.0	1.1	1.3

Table S2.10. Results of interaction with nanoFAST-R52E

Cmpd	Enhancement			
	430	480	530	580
**HBR-DOM2**	4.4	34.3	90.2	1.6
**HBR-2,5-DM**	1.2	2.0	1.3	1.0
**HMBR**	1.1	1.1	1.0	1.3
**HBR-DOM**	1.1	1.1	1.3	0.9
**N 871b**	1.0	1.0	1.0	1.1
**MID 145**	1.2	1.3	1.2	1.0
**N 1052**	1.1	1.4	1.6	1.4
**N 1184**	1.0	1.0	1.0	1.0
**N 1122**	1.1	1.2	1.1	1.1
**N 1049**	1.1	1.3	1.4	1.1
**N 1123**	1.0	1.0	1.0	1.0
**N 1139**	1.0	1.1	1.6	1.8
**N 1118**	1.1	1.2	1.2	1.4
**SAI 112**	1.3	1.9	1.3	1.1
**N 1135**	1.0	1.0	1.0	0.9
**SAI 118**	1.0	1.0	1.0	1.0
**N 1124**	1.0	1.0	0.9	1.1
**SAI 122**	1.9	2.1	1.1	1.0
**N 1142**	1.1	1.2	1.3	1.1
**SAI 199**	1.8	1.9	1.0	1.1
**N 967**	1.0	1.0	1.0	1.1
**N 1039**	1.2	1.3	1.3	1.2
**N 1202**	1.0	1.0	1.0	1.0
**N 960b**	1.0	1.1	1.2	1.3
**N 1036**	1.0	1.0	1.0	0.9
**SAI 117**	1.1	1.2	1.1	1.1
**N 1042**	1.0	1.0	1.0	1.0
**SAI 121**	1.0	1.1	1.1	1.1
**MID 147**	1.6	3.1	4.4	2.2
**SAI 125**	1.0	1.1	1.1	1.1
**MID 151**	1.0	1.0	1.0	1.0
**N 865**	1.0	1.0	1.0	0.9
**MID 153**	1.0	1.0	1.0	1.0
**MID 343**	1.0	1.1	1.6	2.3
**M 3007a**	1.0	1.0	1.1	1.0
**MID 367**	1.0	1.0	1.1	1.1
**N 1048**	1.1	1.1	1.3	1.4
**N 979**	1.0	1.0	1.0	1.1
**N 971**	1.0	1.0	1.0	1.0
**N 973**	1.0	1.0	1.1	1.1
**N 976**	1.0	1.0	1.0	0.9
**N 980**	1.0	1.0	1.1	0.9
**N 1205**	1.5	1.5	1.5	1.4
**N 960a**	1.0	1.0	1.1	1.0

**ZS 331**	1.1	1.3	1.4	1.3
**N 1027**	1.0	1.0	1.0	0.9
**M 2766**	1.0	1.1	1.1	1.0
**ZS 362**	1.0	1.0	1.0	0.9
**N 1179**	1.0	1.0	1.0	1.0
**N 1180**	1.0	1.0	1.0	0.8
**N 1204**	1.3	1.4	1.2	1.4
**N 1206**	1.0	1.0	1.0	1.0
**ZS 309**	1.1	1.3	1.6	1.2
**ZS 316**	1.0	1.0	1.0	1.0
**ZS 319**	1.0	1.0	1.0	1.0
**ZS 325**	1.0	1.0	1.0	1.0
**MID 213**	1.0	1.0	1.1	0.9
**M 2767**	1.3	2.6	4.7	4.1
**M 2876**	1.0	1.0	1.0	1.0
**SH 16**	1.0	1.0	1.0	0.9
**SAI 472**	1.1	1.2	1.2	1.1
**SAI 499**	1.0	1.0	1.0	1.1
**SAI 503**	1.0	1.0	1.0	1.1
**SAI 474**	1.0	1.0	1.0	0.9
**SAI 477**	1.7	1.5	1.0	0.9
**N 1196**	1.0	1.0	1.0	1.0
**N 1197**	1.0	1.0	1.0	0.9
**SAI 127**	1.1	1.5	1.1	0.9
**N 1056**	1.0	1.0	1.1	1.2
**N 1068**	1.0	1.0	1.0	1.0
**N 1069**	1.0	1.0	1.1	1.1
**A9**	1.0	1.0	1.0	0.9
**N 1198**	1.0	1.0	1.0	0.9
**N 1193**	1.4	1.9	1.8	2.0
**N 1199**	1.1	1.1	1.3	1.1
**SAI 363**	2.5	5.1	1.1	1.1
**SAI 366**	6.5	15.9	2.3	1.1
**SAI 362**	1.6	2.9	1.1	1.1
**SAI 365**	5.4	13.2	2.6	0.9
**SAI 120**	1.0	1.1	1.1	1.1
**SAI 458**	1.1	1.1	1.0	1.1
**SAI 459**	1.0	1.0	1.0	0.8
**SAI 487**	1.0	1.0	1.0	1.0
**N 1131**	1.0	1.0	1.0	1.4
**MID 323**	1.0	1.1	1.2	1.4
**SAI 379**	1.0	1.1	1.0	1.1
**SAI 367**	1.2	1.1	1.0	0.9
**M 2738d**	1.0	1.2	1.4	1.0
**ZS 260**	1.3	1.9	1.9	1.2
**N 871a**	1.3	2.0	2.1	1.3
**N 901**	1.0	0.9	1.0	1.2

Table S2.11. Results of interaction with nanoFAST-R52L

Cmpd	Enhancement			
	430	480	530	580
**HBR-DOM2**	3.9	26.8	77.6	1.4
**HBR-2,5-DM**	1.2	2.1	1.3	1.4
**HMBR**	1.2	1.5	1.0	0.6
**HBR-DOM**	1.0	1.1	1.4	1.7
**N 871b**	1.0	1.1	1.6	1.9
**MID 145**	1.1	1.3	1.4	1.0
**N 1052**	1.1	1.4	1.7	1.3
**N 1184**	1.0	1.2	1.5	1.4
**N 1122**	1.1	1.2	1.1	1.8
**N 1049**	1.0	1.2	1.6	1.8
**N 1123**	1.0	1.0	1.1	1.3
**N 1139**	1.0	1.1	1.7	2.4
**N 1118**	1.1	1.2	1.2	1.1
**SAI 112**	1.2	1.8	1.4	1.2
**N 1135**	1.0	1.0	1.1	1.2
**SAI 118**	1.0	1.1	1.0	1.0
**N 1124**	1.0	1.0	1.1	1.3
**SAI 122**	4.1	5.0	1.2	1.3
**N 1142**	1.1	1.3	1.5	1.2
**SAI 199**	4.5	5.4	1.0	0.7
**N 967**	1.0	1.0	1.2	1.6
**N 1039**	1.1	1.3	1.7	1.9
**N 1202**	1.0	1.1	1.6	1.8
**N 960b**	1.0	1.1	1.5	1.8
**N 1036**	1.0	1.0	1.0	1.1
**SAI 117**	1.1	1.4	1.1	0.7
**N 1042**	1.0	0.9	1.0	1.0
**SAI 121**	1.1	1.2	1.1	0.8
**MID 147**	1.6	3.1	4.4	2.5
**SAI 125**	1.1	1.4	1.0	0.6
**MID 151**	1.0	1.0	1.0	1.2
**N 865**	1.0	1.0	1.0	1.3
**MID 153**	1.0	0.9	0.9	1.1
**MID 343**	1.0	1.1	1.6	3.7
**M 3007a**	1.0	1.0	1.2	1.1
**MID 367**	1.0	1.0	1.2	1.2
**N 1048**	1.0	1.1	1.3	1.6
**N 979**	1.0	1.0	1.0	0.9
**N 971**	1.0	1.0	1.0	0.9
**N 973**	1.0	1.0	1.1	0.7
**N 976**	1.0	1.0	1.0	1.2
**N 980**	1.0	1.0	1.0	0.8
**N 1205**	1.3	1.6	2.3	1.7
**N 960a**	1.0	1.1	1.1	1.2

**ZS 331**	1.1	1.2	1.4	1.5
**N 1027**	1.0	1.0	1.0	0.8
**M 2766**	1.0	1.2	1.5	1.5
**ZS 362**	1.0	1.0	1.0	0.8
**N 1179**	1.0	1.0	1.0	0.9
**N 1180**	1.0	1.0	1.0	1.1
**N 1204**	1.3	1.6	2.0	1.8
**N 1206**	1.0	1.0	1.0	0.9
**ZS 309**	1.1	1.3	1.7	1.6
**ZS 316**	1.0	1.0	1.0	1.0
**ZS 319**	1.0	1.0	1.0	1.0
**ZS 325**	1.0	1.0	1.0	1.3
**MID 213**	1.0	1.0	1.1	1.1
**M 2767**	1.2	2.0	3.7	3.8
**M 2876**	1.0	1.0	1.0	1.0
**SH 16**	1.0	1.1	1.2	0.9
**SAI 472**	1.1	1.2	1.1	1.0
**SAI 499**	1.2	1.1	1.0	0.8
**SAI 503**	1.0	1.0	1.0	1.0
**SAI 474**	1.1	1.0	1.0	1.0
**SAI 477**	1.8	1.5	1.0	1.3
**N 1196**	1.0	1.0	1.0	0.9
**N 1197**	1.1	1.0	1.0	1.2
**SAI 127**	1.2	1.8	1.3	0.8
**N 1056**	1.0	1.0	1.1	1.0
**N 1068**	1.0	1.0	1.1	1.2
**N 1069**	1.0	1.0	1.1	1.0
**A9**	1.0	1.0	1.0	0.9
**N 1198**	1.0	1.0	1.0	1.1
**N 1193**	1.7	2.3	2.2	2.5
**N 1199**	1.1	1.2	1.4	1.0
**SAI 363**	6.2	16.2	1.4	0.9
**SAI 366**	8.3	22.9	3.3	1.1
**SAI 362**	7.4	22.7	1.8	0.9
**SAI 365**	6.4	17.6	3.4	1.2
**SAI 120**	1.1	1.1	1.2	0.9
**SAI 458**	1.5	2.7	1.0	0.8
**SAI 459**	1.0	1.0	1.0	1.0
**SAI 487**	1.0	1.0	1.0	0.9
**N 1131**	1.0	1.0	1.0	1.0
**MID 323**	1.0	1.1	1.3	1.8
**SAI 379**	0.9	1.0	1.0	0.9
**SAI 367**	1.3	1.2	1.1	0.9
**M 2738d**	1.1	2.2	3.3	1.3
**ZS 260**	1.4	2.1	2.2	1.3
**N 871a**	1.3	1.9	2.1	1.5
**N 901**	1.0	1.0	1.1	1.2

Table S2.12. Results of interaction with nanoFAST-R52N

Cmpd	Enhancement			
	430	480	530	580
**HBR-DOM2**	9.4	60.8	95.9	2.0
**HBR-2,5-DM**	1.5	3.2	1.8	1.2
**HMBR**	1.5	1.6	1.1	0.9
**HBR-DOM**	1.0	1.3	2.0	1.6
**N 871b**	1.2	2.0	3.7	3.8
**MID 145**	1.2	1.8	2.6	1.3
**N 1052**	1.2	1.7	2.5	1.8
**N 1184**	1.2	1.9	3.3	1.6
**N 1122**	1.1	1.2	1.2	1.1
**N 1049**	1.1	1.5	2.2	1.5
**N 1123**	1.0	0.9	0.9	0.7
**N 1139**	1.0	1.2	2.1	3.5
**N 1118**	1.1	1.4	1.4	1.1
**SAI 112**	1.4	2.6	1.4	1.1
**N 1135**	1.0	1.0	1.0	1.1
**SAI 118**	1.2	1.5	1.1	1.3
**N 1124**	1.0	1.0	1.0	1.2
**SAI 122**	5.8	5.3	1.2	1.3
**N 1142**	1.1	1.2	1.3	1.1
**SAI 199**	8.1	7.4	1.0	1.2
**N 967**	1.1	1.2	1.6	2.0
**N 1039**	1.3	2.1	3.8	2.2
**N 1202**	1.2	2.0	3.7	2.9
**N 960b**	1.1	1.3	2.2	3.3
**N 1036**	1.0	1.0	1.0	1.3
**SAI 117**	1.3	1.8	1.1	1.3
**N 1042**	1.0	1.0	1.0	1.1
**SAI 121**	1.4	1.5	1.1	1.1
**MID 147**	1.8	3.8	6.3	4.8
**SAI 125**	1.3	1.8	1.1	1.1
**MID 151**	1.0	1.0	1.1	1.2
**N 865**	1.0	1.0	1.0	1.0
**MID 153**	1.0	1.0	1.0	1.0
**MID 343**	1.0	1.1	1.9	4.0
**M 3007a**	1.0	1.0	1.2	1.1
**MID 367**	1.0	1.0	1.2	1.8
**N 1048**	1.1	1.2	1.6	1.8
**N 979**	1.0	1.0	1.0	1.0
**N 971**	1.0	1.0	1.0	0.9
**N 973**	1.0	1.1	1.1	1.4
**N 976**	1.0	1.0	1.0	0.9
**N 980**	1.0	1.0	1.1	0.9
**N 1205**	1.9	3.9	6.7	3.4
**N 960a**	1.0	1.1	1.2	1.6

**ZS 331**	1.1	1.4	1.9	2.4
**N 1027**	1.0	1.0	1.0	0.7
**M 2766**	1.2	2.2	3.7	2.7
**ZS 362**	1.0	1.0	1.0	1.1
**N 1179**	1.0	1.0	1.0	1.7
**N 1180**	1.0	1.0	1.0	1.1
**N 1204**	1.4	2.4	3.7	2.0
**N 1206**	1.0	1.0	1.0	0.8
**ZS 309**	1.1	1.3	1.7	2.2
**ZS 316**	1.0	0.9	0.9	0.9
**ZS 319**	1.0	1.0	0.9	1.2
**ZS 325**	1.0	1.0	1.0	1.0
**MID 213**	1.0	1.0	1.1	1.1
**M 2767**	1.2	2.1	4.4	5.5
**M 2876**	1.0	1.0	1.0	0.9
**SH 16**	1.1	1.1	1.2	0.6
**SAI 472**	1.1	1.3	1.1	0.8
**SAI 499**	1.0	1.0	1.0	0.6
**SAI 503**	1.0	1.0	1.0	1.0
**SAI 474**	1.1	1.0	1.0	1.0
**SAI 477**	2.1	1.4	1.0	0.9
**N 1196**	1.0	1.0	1.1	1.4
**N 1197**	1.0	1.0	1.0	1.9
**SAI 127**	1.2	2.0	1.2	1.2
**N 1056**	1.0	1.0	1.1	1.8
**N 1068**	1.0	1.0	1.1	1.1
**N 1069**	1.0	1.0	1.1	1.1
**A9**	1.0	1.0	1.0	0.8
**N 1198**	1.0	1.0	1.0	0.9
**N 1193**	1.7	2.2	1.9	2.5
**N 1199**	1.1	1.2	1.5	1.3
**SAI 363**	2.0	3.4	1.1	1.5
**SAI 366**	6.3	15.3	2.4	1.2
**SAI 362**	9.1	23.0	1.6	1.1
**SAI 365**	4.9	11.7	2.7	1.0
**SAI 120**	1.0	1.1	1.1	1.3
**SAI 458**	2.3	3.8	1.1	1.3
**SAI 459**	1.0	1.0	1.0	0.8
**SAI 487**	1.0	1.0	1.0	0.7
**N 1131**	1.0	1.0	1.0	1.2
**MID 323**	1.0	1.1	1.2	2.4
**SAI 379**	1.0	1.2	1.0	0.8
**SAI 367**	1.2	1.1	1.1	1.3
**M 2738d**	1.0	1.4	1.8	1.1
**ZS 260**	1.4	2.1	2.0	1.1
**N 871a**	1.7	3.2	4.4	3.7
**N 901**	0.9	0.8	0.9	1.2

Table S2.13. Results of interaction with nanoFAST-R52Q

Cmpd	Enhancement			
	430	480	530	580
**HBR-DOM2**	10.0	86.3	222.0	2.9
**HBR-2,5-DM**	1.6	3.8	1.7	1.2
**HMBR**	1.2	1.5	1.1	1.2
**HBR-DOM**	1.0	1.2	1.8	1.1
**N 871b**	1.0	1.2	1.6	1.5
**MID 145**	1.0	1.0	1.3	0.9
**N 1052**	1.0	1.1	1.2	1.3
**N 1184**	1.0	1.2	1.5	1.1
**N 1122**	1.0	1.2	1.1	0.8
**N 1049**	1.0	1.1	1.2	1.2
**N 1123**	1.0	1.0	1.1	1.1
**N 1139**	1.0	1.1	1.8	2.5
**N 1118**	1.1	1.2	1.2	0.9
**SAI 112**	1.5	2.4	1.5	1.0
**N 1135**	1.0	1.0	1.1	0.9
**SAI 118**	1.1	1.2	1.1	1.0
**N 1124**	1.0	1.0	1.1	1.2
**SAI 122**	3.4	4.4	1.2	1.3
**N 1142**	1.0	1.1	1.1	1.3
**SAI 199**	4.5	5.3	1.1	0.9
**N 967**	1.0	1.1	1.4	1.8
**N 1039**	1.0	1.2	1.6	1.3
**N 1202**	1.0	1.2	1.6	1.5
**N 960b**	1.0	1.1	1.4	1.9
**N 1036**	1.0	1.0	1.1	1.6
**SAI 117**	1.2	1.5	1.1	1.0
**N 1042**	1.0	1.0	1.0	1.0
**SAI 121**	1.1	1.3	1.1	1.0
**MID 147**	1.2	1.6	2.4	2.4
**SAI 125**	1.3	1.6	1.1	1.0
**MID 151**	1.0	1.1	1.2	1.4
**N 865**	1.0	1.0	1.0	1.0
**MID 153**	1.0	1.0	1.1	1.2
**MID 343**	1.0	1.1	1.6	3.3
**M 3007a**	1.0	1.0	1.1	1.0
**MID 367**	1.0	1.0	1.2	1.4
**N 1048**	1.0	1.3	2.3	3.7
**N 979**	1.0	1.0	1.0	1.0
**N 971**	1.0	1.0	1.0	1.0
**N 973**	1.0	1.1	1.1	1.3
**N 976**	1.0	1.0	1.0	1.4
**N 980**	1.0	1.0	1.0	0.9
**N 1205**	1.4	1.8	2.6	2.1
**N 960a**	1.0	1.1	1.2	1.3

**ZS 331**	1.1	1.2	1.5	1.8
**N 1027**	1.0	1.0	1.0	0.8
**M 2766**	1.0	1.2	1.6	1.6
**ZS 362**	1.0	1.0	1.0	0.8
**N 1179**	1.0	1.0	1.0	1.1
**N 1180**	1.0	1.0	1.1	1.3
**N 1204**	1.2	1.6	1.9	1.6
**N 1206**	1.0	1.0	1.1	1.0
**ZS 309**	1.2	1.5	1.9	1.9
**ZS 316**	1.0	1.0	1.0	1.0
**ZS 319**	1.0	1.0	1.0	1.0
**ZS 325**	1.0	1.0	1.1	1.0
**MID 213**	1.0	1.1	1.2	1.0
**M 2767**	1.3	2.5	4.5	5.4
**M 2876**	1.0	1.0	1.0	1.0
**SH 16**	1.1	1.1	1.1	1.0
**SAI 472**	1.2	1.5	1.3	1.2
**SAI 499**	1.1	1.1	1.0	0.9
**SAI 503**	1.0	1.0	1.0	0.9
**SAI 474**	1.0	1.0	1.0	1.4
**SAI 477**	3.7	2.5	1.1	1.1
**N 1196**	1.0	1.0	1.1	1.0
**N 1197**	1.0	1.0	1.1	1.2
**SAI 127**	1.3	2.0	1.2	0.8
**N 1056**	1.0	1.0	1.1	1.2
**N 1068**	1.0	1.0	1.0	1.0
**N 1069**	1.0	1.1	1.3	0.9
**A9**	1.0	1.0	1.0	1.1
**N 1198**	1.0	1.0	1.1	1.1
**N 1193**	1.6	2.1	2.5	2.7
**N 1199**	1.0	1.1	1.3	1.3
**SAI 363**	6.8	14.8	1.1	1.3
**SAI 366**	11.4	30.1	2.4	1.0
**SAI 362**	3.7	9.9	1.3	0.7
**SAI 365**	9.3	24.1	2.6	1.3
**SAI 120**	1.0	1.1	1.1	0.9
**SAI 458**	1.3	1.9	1.0	0.9
**SAI 459**	1.0	1.0	1.0	1.2
**SAI 487**	1.0	1.0	1.0	0.9
**N 1131**	1.0	1.0	1.0	1.1
**MID 323**	1.0	1.1	1.3	1.8
**SAI 379**	1.0	1.2	1.1	0.9
**SAI 367**	1.2	1.1	1.0	1.0
**M 2738d**	1.0	1.5	2.0	1.3
**ZS 260**	1.5	2.1	2.0	1.3
**N 871a**	1.4	2.2	2.6	1.7
**N 901**	1.0	1.0	1.2	1.5

Table S2.14. Results of interaction with nanoFAST-R52I

Cmpd	Enhancement			
	430	480	530	580
**HBR-DOM2**	7.7	72.0	218.9	4.2
**HBR-2,5-DM**	1.4	2.8	1.6	0.9
**HMBR**	1.2	1.5	1.0	1.2
**HBR-DOM**	1.1	1.2	2.0	1.5
**N 871b**	1.0	1.1	1.4	1.6
**MID 145**	1.1	1.2	1.3	1.3
**N 1052**	1.1	1.4	1.6	1.5
**N 1184**	1.0	1.1	1.4	1.1
**N 1122**	1.1	1.2	1.1	1.0
**N 1049**	1.1	1.2	1.4	1.2
**N 1123**	1.0	1.0	1.0	1.1
**N 1139**	1.0	1.1	1.7	2.8
**N 1118**	1.1	1.2	1.1	1.0
**SAI 112**	1.3	2.2	1.5	1.1
**N 1135**	1.0	1.0	1.1	1.2
**SAI 118**	1.0	1.1	1.0	0.8
**N 1124**	1.0	1.0	1.1	1.4
**SAI 122**	3.0	3.9	1.1	1.4
**N 1142**	1.1	1.2	1.5	1.3
**SAI 199**	3.7	4.8	1.0	0.9
**N 967**	1.0	1.2	1.7	2.6
**N 1039**	1.1	1.3	1.5	1.5
**N 1202**	1.0	1.1	1.5	1.7
**N 960b**	1.0	1.1	1.4	2.1
**N 1036**	1.0	1.0	1.0	1.2
**SAI 117**	1.1	1.4	1.0	1.0
**N 1042**	1.0	1.0	1.0	0.7
**SAI 121**	1.1	1.2	1.2	1.0
**MID 147**	1.4	2.5	3.2	2.3
**SAI 125**	1.1	1.4	1.1	1.0
**MID 151**	1.0	1.0	1.1	1.3
**N 865**	1.0	1.0	1.0	1.0
**MID 153**	1.0	1.0	1.0	1.1
**MID 343**	1.0	1.0	1.3	2.3
**M 3007a**	1.0	1.0	1.1	0.9
**MID 367**	1.0	1.0	1.1	1.6
**N 1048**	1.1	1.2	1.9	3.3
**N 979**	1.0	1.0	1.0	1.3
**N 971**	1.0	1.0	1.0	1.0
**N 973**	1.0	1.1	1.1	1.3
**N 976**	1.0	1.0	1.0	0.9
**N 980**	1.0	1.0	1.0	0.9
**N 1205**	1.2	1.6	2.3	2.2
**N 960a**	1.0	1.0	1.1	0.9

**ZS 331**	1.0	1.1	1.3	1.5
**N 1027**	1.0	1.0	1.0	1.1
**M 2766**	1.0	1.2	1.5	1.5
**ZS 362**	1.0	1.0	1.0	0.8
**N 1179**	1.0	1.0	1.0	1.0
**N 1180**	1.0	1.0	1.0	1.1
**N 1204**	1.1	1.2	1.5	1.2
**N 1206**	1.0	0.9	1.0	1.0
**ZS 309**	1.1	1.2	1.6	1.7
**ZS 316**	1.0	1.0	0.9	0.9
**ZS 319**	1.0	1.0	1.0	1.0
**ZS 325**	1.0	1.0	1.0	1.1
**MID 213**	1.0	1.0	1.0	1.0
**M 2767**	1.3	2.5	4.7	5.7
**M 2876**	1.0	1.0	1.0	1.0
**SH 16**	1.0	1.0	1.1	1.1
**SAI 472**	1.1	1.3	1.2	1.0
**SAI 499**	1.0	1.0	1.0	0.9
**SAI 503**	1.0	1.0	1.0	1.1
**SAI 474**	1.0	1.0	1.0	1.1
**SAI 477**	3.1	2.6	1.0	1.2
**N 1196**	1.0	1.0	1.0	0.9
**N 1197**	1.0	1.0	1.0	1.0
**SAI 127**	1.2	1.9	1.3	1.1
**N 1056**	1.0	1.0	1.1	1.4
**N 1068**	1.0	1.0	1.0	1.0
**N 1069**	1.0	1.0	1.1	0.9
**A9**	1.0	0.9	0.9	1.0
**N 1198**	1.0	1.0	1.0	1.1
**N 1193**	1.2	1.4	1.6	2.0
**N 1199**	1.0	1.1	1.2	1.1
**SAI 363**	4.8	13.5	1.2	1.2
**SAI 366**	9.2	24.8	3.3	0.8
**SAI 362**	3.5	11.4	1.5	0.9
**SAI 365**	8.6	23.8	3.7	1.1
**SAI 120**	1.1	1.1	1.1	1.0
**SAI 458**	1.2	1.8	1.0	1.0
**SAI 459**	1.0	1.0	1.0	1.1
**SAI 487**	1.0	1.0	1.0	1.1
**N 1131**	1.0	1.0	1.0	0.9
**MID 323**	1.0	1.1	1.4	2.0
**SAI 379**	0.9	1.1	1.0	1.1
**SAI 367**	1.3	1.1	1.0	1.0
**M 2738d**	1.1	2.2	3.0	1.2
**ZS 260**	1.2	1.6	1.6	1.2
**N 871a**	1.3	1.9	1.9	1.4
**N 901**	1.0	1.0	1.1	1.4

Table S2.15. Results of interaction with nanoFAST-R52A

Cmpd	Enhancement			
	430	480	530	580
**HBR-DOM2**	11.0	108.9	>333.5	3.0
**HBR-2,5-DM**	1.3	3.1	2.3	1.2
**HMBR**	1.1	1.3	1.0	1.1
**HBR-DOM**	1.0	1.5	3.5	1.4
**N 871b**	1.0	1.1	1.3	1.3
**MID 145**	1.1	1.3	1.4	1.0
**N 1052**	1.1	1.4	1.5	1.1
**N 1184**	1.0	1.1	1.3	1.1
**N 1122**	1.1	1.2	1.2	1.1
**N 1049**	1.0	1.2	1.4	1.2
**N 1123**	1.0	1.0	1.0	1.1
**N 1139**	1.0	1.1	2.0	2.9
**N 1118**	1.1	1.3	1.3	1.3
**SAI 112**	1.5	3.2	1.5	1.0
**N 1135**	1.0	1.0	1.0	1.1
**SAI 118**	1.0	1.5	1.1	0.9
**N 1124**	1.0	1.0	1.0	1.0
**SAI 122**	1.9	2.6	1.1	1.1
**N 1142**	1.0	1.2	1.3	1.2
**SAI 199**	2.0	2.9	1.0	0.9
**N 967**	1.0	1.0	1.2	1.2
**N 1039**	1.1	1.3	1.6	1.2
**N 1202**	1.0	1.0	1.3	1.2
**N 960b**	1.0	1.1	1.3	1.6
**N 1036**	1.0	1.1	1.0	1.0
**SAI 117**	1.3	1.1	0.3	0.6
**N 1042**	1.0	1.0	1.1	1.2
**SAI 121**	1.1	1.3	1.1	1.1
**MID 147**	1.5	2.7	3.8	2.1
**SAI 125**	1.2	1.9	1.1	1.2
**MID 151**	1.0	1.0	1.0	1.1
**N 865**	1.0	1.0	1.0	1.0
**MID 153**	1.0	0.9	1.0	1.0
**MID 343**	1.0	1.1	1.4	2.1
**M 3007a**	1.0	1.0	1.1	1.2
**MID 367**	1.0	1.0	1.1	1.3
**N 1048**	1.1	1.4	3.9	7.5
**N 979**	1.0	1.0	1.0	1.0
**N 971**	1.0	1.0	1.0	1.1
**N 973**	1.0	1.1	1.1	1.1
**N 976**	1.0	1.0	1.0	0.9
**N 980**	1.0	1.0	1.0	0.9
**N 1205**	1.4	1.7	2.1	1.6
**N 960a**	1.0	1.0	1.1	1.1

**ZS 331**	1.0	1.2	1.3	1.2
**N 1027**	1.0	1.0	1.0	1.0
**M 2766**	1.0	1.2	1.3	1.2
**ZS 362**	1.0	1.0	1.0	1.1
**N 1179**	0.9	0.9	1.0	1.1
**N 1180**	0.9	0.9	1.0	1.1
**N 1204**	1.1	1.1	1.3	1.1
**N 1206**	0.9	0.9	1.0	1.1
**ZS 309**	1.0	1.2	1.7	1.2
**ZS 316**	0.9	0.9	1.0	1.0
**ZS 319**	0.9	0.9	0.9	1.2
**ZS 325**	0.9	0.9	1.1	0.8
**MID 213**	0.9	0.9	1.0	0.9
**M 2767**	1.1	1.7	4.7	5.5
**M 2876**	0.9	0.9	1.0	0.8
**SH 16**	1.0	0.9	1.0	0.9
**SAI 472**	1.1	1.3	1.5	1.1
**SAI 499**	0.9	0.9	1.0	1.4
**SAI 503**	0.9	0.9	0.9	0.7
**SAI 474**	0.9	0.9	1.0	0.8
**SAI 477**	4.4	2.6	1.0	0.9
**N 1196**	0.9	0.9	1.0	1.0
**N 1197**	1.0	0.9	1.1	1.2
**SAI 127**	1.2	1.4	1.1	1.0
**N 1056**	0.9	0.9	1.1	0.9
**N 1068**	0.9	0.9	1.0	1.1
**N 1069**	0.9	0.9	1.2	1.1
**A9**	0.9	0.9	0.9	0.8
**N 1198**	0.9	0.9	1.0	1.0
**N 1193**	1.4	1.6	2.2	2.2
**N 1199**	1.0	1.0	1.4	1.0
**SAI 363**	2.1	3.5	1.1	1.1
**SAI 366**	8.8	20.4	2.8	1.0
**SAI 362**	1.4	2.4	1.3	1.1
**SAI 365**	9.8	23.5	3.5	0.8
**SAI 120**	1.0	1.0	1.1	1.2
**SAI 458**	1.0	1.0	1.0	1.0
**SAI 459**	0.9	0.9	1.0	1.1
**SAI 487**	0.9	0.9	1.0	1.0
**N 1131**	1.0	0.9	1.0	0.9
**MID 323**	1.0	1.0	1.2	1.5
**SAI 379**	1.0	1.1	1.0	1.0
**SAI 367**	1.1	1.0	1.0	0.9
**M 2738d**	1.0	1.4	2.3	1.1
**ZS 260**	1.3	1.9	2.1	1.2
**N 871a**	1.3	1.9	2.3	1.4
**N 901**	1.0	1.0	1.1	1.3

Table S2.16. Results of interaction with nanoFAST-R52Y

Cmpd	Enhancement			
	430	480	530	580
**HBR-DOM2**	4.2	33.2	81.5	1.6
**HBR-2,5-DM**	1.1	1.8	1.3	1.0
**HMBR**	1.1	1.2	1.0	0.9
**HBR-DOM**	1.0	1.1	1.3	1.3
**N 871b**	1.1	1.3	1.8	2.2
**MID 145**	1.1	1.4	1.6	1.2
**N 1052**	1.1	1.4	1.7	1.4
**N 1184**	1.0	1.1	1.6	1.2
**N 1122**	1.1	1.2	1.2	1.5
**N 1049**	1.1	1.3	1.6	1.4
**N 1123**	1.0	1.0	1.0	1.0
**N 1139**	1.0	1.1	1.8	3.0
**N 1118**	1.1	1.2	1.2	1.1
**SAI 112**	1.2	1.7	1.4	1.0
**N 1135**	1.0	1.0	1.1	1.1
**SAI 118**	0.9	1.0	1.0	1.0
**N 1124**	1.0	1.0	1.0	1.2
**SAI 122**	2.7	3.2	1.1	1.2
**N 1142**	1.1	1.2	1.3	1.2
**SAI 199**	2.6	3.1	1.0	1.0
**N 967**	1.0	1.1	1.2	1.6
**N 1039**	1.1	1.6	2.3	1.5
**N 1202**	1.0	1.2	2.0	2.0
**N 960b**	1.0	1.2	1.7	2.8
**N 1036**	1.0	1.0	1.0	1.2
**SAI 117**	1.0	1.2	1.0	0.8
**N 1042**	1.0	1.0	1.0	1.0
**SAI 121**	1.0	1.1	1.1	1.1
**MID 147**	1.5	2.7	4.1	2.5
**SAI 125**	1.0	1.1	1.0	1.0
**MID 151**	1.0	1.0	1.0	1.2
**N 865**	1.0	1.0	1.0	1.0
**MID 153**	1.0	1.0	1.1	1.1
**MID 343**	1.0	1.1	1.3	1.8
**M 3007a**	1.0	1.0	1.1	1.1
**MID 367**	1.0	1.0	1.1	1.2
**N 1048**	1.0	1.1	1.2	1.6
**N 979**	1.0	1.0	1.0	1.0
**N 971**	1.0	1.0	1.0	1.1
**N 973**	1.1	1.1	1.1	1.0
**N 976**	1.0	1.0	1.0	1.0
**N 980**	1.0	1.0	1.0	1.1
**N 1205**	1.4	2.0	3.0	1.6
**N 960a**	1.0	1.1	1.1	1.1

**ZS 331**	1.1	1.2	1.4	1.4
**N 1027**	1.0	1.0	1.0	1.0
**M 2766**	1.1	1.2	1.7	1.5
**ZS 362**	1.0	1.0	1.0	1.0
**N 1179**	1.0	1.0	1.0	1.1
**N 1180**	1.0	1.0	1.0	1.1
**N 1204**	1.2	1.6	2.1	1.4
**N 1206**	1.0	0.9	1.0	1.1
**ZS 309**	1.1	1.3	1.7	1.3
**ZS 316**	1.0	1.0	1.0	0.9
**ZS 319**	1.0	0.9	0.9	0.9
**ZS 325**	1.0	1.0	1.0	1.1
**MID 213**	1.0	1.0	1.1	1.2
**M 2767**	1.2	1.7	3.0	3.7
**M 2876**	1.0	1.0	1.0	1.0
**SH 16**	1.1	1.1	1.1	1.0
**SAI 472**	1.1	1.2	1.2	0.9
**SAI 499**	1.0	1.0	1.0	1.0
**SAI 503**	1.0	1.0	1.0	1.0
**SAI 474**	1.0	1.0	1.0	1.0
**SAI 477**	1.6	1.3	1.0	0.9
**N 1196**	1.0	1.0	1.0	0.9
**N 1197**	1.0	1.0	1.1	1.0
**SAI 127**	1.1	1.3	1.2	1.2
**N 1056**	1.0	1.0	1.0	1.0
**N 1068**	1.0	1.0	1.0	1.1
**N 1069**	1.0	1.0	1.2	1.0
**A9**	1.0	1.0	1.0	0.9
**N 1198**	1.0	1.0	1.0	0.9
**N 1193**	1.7	2.5	3.1	3.5
**N 1199**	1.1	1.2	1.4	1.0
**SAI 363**	1.5	2.3	1.1	1.0
**SAI 366**	6.1	15.4	2.5	0.9
**SAI 362**	2.5	6.0	1.2	1.0
**SAI 365**	5.9	15.5	3.0	0.9
**SAI 120**	1.0	1.1	1.1	0.9
**SAI 458**	1.1	1.4	1.0	1.1
**SAI 459**	1.0	1.0	0.9	0.9
**SAI 487**	1.0	1.0	1.0	1.0
**N 1131**	1.0	1.0	1.0	0.9
**MID 323**	1.0	1.0	1.2	1.5
**SAI 379**	1.0	1.1	1.0	1.0
**SAI 367**	1.3	1.1	1.0	1.0
**M 2738d**	1.0	1.1	1.3	1.0
**ZS 260**	1.5	2.4	2.9	1.8
**N 871a**	1.3	2.0	2.5	1.8
**N 901**	1.0	1.0	1.1	1.4

Table S2.17. Results of interaction with nanoFAST-R52T

Cmpd	Enhancement			
	430	480	530	580
**HBR-DOM2**	10.5	96.0	249.1	3.4
**HBR-2,5-DM**	1.3	2.3	1.4	1.0
**HMBR**	1.1	1.2	1.0	1.1
**HBR-DOM**	1.1	1.4	2.3	1.6
**N 871b**	1.0	1.1	1.2	1.4
**MID 145**	1.1	1.3	1.3	1.2
**N 1052**	1.1	1.5	1.9	1.4
**N 1184**	1.0	1.0	1.0	1.0
**N 1122**	1.1	1.2	1.2	1.1
**N 1049**	1.1	1.2	1.4	1.3
**N 1123**	1.0	1.0	1.0	1.0
**N 1139**	1.0	1.2	2.0	3.3
**N 1118**	1.1	1.3	1.3	1.3
**SAI 112**	1.3	2.1	1.4	1.1
**N 1135**	1.0	1.0	1.0	1.2
**SAI 118**	1.0	1.0	1.0	1.1
**N 1124**	1.0	1.0	1.0	1.1
**SAI 122**	2.0	2.5	1.1	0.9
**N 1142**	1.1	1.2	1.3	1.3
**SAI 199**	2.0	2.5	1.0	0.9
**N 967**	1.0	1.1	1.3	1.3
**N 1039**	1.1	1.3	1.6	1.4
**N 1202**	1.0	1.0	1.2	1.2
**N 960b**	1.0	1.1	1.4	1.7
**N 1036**	1.0	1.0	1.0	1.1
**SAI 117**	1.2	1.4	1.1	0.9
**N 1042**	1.0	1.0	1.0	1.0
**SAI 121**	1.0	1.1	1.1	1.0
**MID 147**	1.5	2.8	3.7	2.3
**SAI 125**	1.2	1.3	1.0	0.7
**MID 151**	1.0	1.0	1.1	1.2
**N 865**	1.0	1.0	1.0	1.2
**MID 153**	1.0	1.0	1.0	1.1
**MID 343**	1.0	1.1	1.4	2.2
**M 3007a**	1.0	1.0	1.1	1.1
**MID 367**	1.0	1.1	1.1	1.6
**N 1048**	1.1	1.2	2.2	4.0
**N 979**	1.0	1.0	1.0	0.8
**N 971**	1.0	1.0	1.0	1.0
**N 973**	1.0	1.1	1.1	1.2
**N 976**	1.0	1.0	1.0	1.1
**N 980**	1.0	1.0	1.0	1.2
**N 1205**	1.3	1.8	2.2	1.8
**N 960a**	1.0	1.1	1.1	1.2

**ZS 331**	1.1	1.2	1.3	1.4
**N 1027**	1.0	1.0	1.0	1.0
**M 2766**	1.0	1.1	1.2	1.3
**ZS 362**	1.0	1.0	1.0	1.2
**N 1179**	1.0	1.0	1.0	1.1
**N 1180**	1.0	1.0	1.0	0.9
**N 1204**	1.2	1.5	1.8	1.2
**N 1206**	1.0	0.9	1.0	1.1
**ZS 309**	1.1	1.4	1.8	1.6
**ZS 316**	1.0	1.0	1.0	0.8
**ZS 319**	1.0	0.9	1.0	1.0
**ZS 325**	1.0	1.0	1.0	1.1
**MID 213**	1.0	1.0	1.1	1.0
**M 2767**	1.2	2.1	3.9	4.4
**M 2876**	1.0	1.0	1.0	0.9
**SH 16**	1.1	1.0	1.1	1.2
**SAI 472**	1.2	1.6	1.6	1.0
**SAI 499**	1.0	1.0	1.0	0.9
**SAI 503**	1.0	1.0	1.0	0.9
**SAI 474**	1.0	1.0	1.0	0.9
**SAI 477**	4.3	3.2	1.0	1.0
**N 1196**	1.0	1.0	1.0	1.0
**N 1197**	1.0	1.0	1.0	1.1
**SAI 127**	1.2	1.7	1.2	1.0
**N 1056**	1.0	1.0	1.2	1.2
**N 1068**	1.0	1.0	1.0	1.1
**N 1069**	1.0	1.0	1.2	1.1
**A9**	1.0	0.9	1.0	1.0
**N 1198**	0.9	1.0	1.0	1.0
**N 1193**	1.3	1.9	2.4	2.1
**N 1199**	1.0	1.1	1.3	1.1
**SAI 363**	3.0	7.3	1.2	0.9
**SAI 366**	10.7	29.7	3.1	1.0
**SAI 362**	1.7	3.8	1.3	0.9
**SAI 365**	12.0	34.4	4.3	1.1
**SAI 120**	1.0	1.1	1.1	1.1
**SAI 458**	1.1	1.2	1.0	0.9
**SAI 459**	1.0	1.0	1.0	0.9
**SAI 487**	1.0	1.0	1.0	1.0
**N 1131**	1.0	1.0	1.0	1.0
**MID 323**	1.0	1.0	1.2	1.3
**SAI 379**	1.0	1.2	1.0	0.9
**SAI 367**	1.2	1.1	1.0	1.0
**M 2738d**	1.0	1.4	1.9	1.1
**ZS 260**	1.4	2.2	2.4	1.4
**N 871a**	1.4	2.3	2.7	1.6
**N 901**	1.0	1.0	1.2	1.4

Table S2.18. Results of interaction with nanoFAST-R52V

Cmpd	Enhancement			
	430	480	530	580
**HBR-DOM2**	9.8	88.6	264.3	4.1
**HBR-2,5-DM**	1.4	2.9	1.7	1.1
**HMBR**	1.2	1.3	1.0	0.9
**HBR-DOM**	1.1	1.3	2.5	2.0
**N 871b**	1.0	1.1	1.4	1.5
**MID 145**	1.1	1.3	1.4	1.2
**N 1052**	1.1	1.4	1.7	1.3
**N 1184**	1.0	1.1	1.4	1.7
**N 1122**	1.1	1.2	1.1	0.8
**N 1049**	1.1	1.4	1.7	1.1
**N 1123**	1.0	1.0	1.0	0.7
**N 1139**	1.0	1.1	1.8	2.5
**N 1118**	1.1	1.2	1.1	1.1
**SAI 112**	1.3	2.3	1.4	0.8
**N 1135**	1.0	1.0	1.0	0.8
**SAI 118**	1.0	1.1	1.1	0.6
**N 1124**	1.0	1.0	1.1	1.1
**SAI 122**	2.7	3.5	1.1	0.8
**N 1142**	1.1	1.3	1.5	1.4
**SAI 199**	3.0	3.9	1.1	1.1
**N 967**	1.0	1.1	1.2	1.4
**N 1039**	1.1	1.3	1.6	1.5
**N 1202**	1.0	1.1	1.5	1.5
**N 960b**	1.0	1.2	1.5	1.8
**N 1036**	1.0	1.0	1.0	1.0
**SAI 117**	1.1	1.5	1.0	1.2
**N 1042**	1.0	1.0	1.0	1.0
**SAI 121**	1.0	1.2	1.1	0.9
**MID 147**	1.5	2.8	3.5	2.1
**SAI 125**	1.2	1.4	1.1	0.7
**MID 151**	1.0	1.0	1.0	1.0
**N 865**	1.0	1.0	1.0	1.1
**MID 153**	1.0	1.0	1.0	1.1
**MID 343**	1.0	1.1	1.3	1.8
**M 3007a**	1.0	1.0	1.1	0.9
**MID 367**	1.0	1.0	1.1	1.4
**N 1048**	1.1	1.4	2.9	5.2
**N 979**	1.0	1.0	1.0	1.3
**N 971**	1.0	1.0	1.0	1.2
**N 973**	1.0	1.1	1.1	1.1
**N 976**	1.0	1.0	1.0	1.3
**N 980**	1.0	1.0	1.0	1.0
**N 1205**	1.3	1.6	2.2	1.8
**N 960a**	1.0	1.0	1.1	1.3

**ZS 331**	1.1	1.2	1.3	1.4
**N 1027**	1.0	1.0	1.0	0.9
**M 2766**	1.1	1.2	1.4	1.4
**ZS 362**	1.0	1.0	1.0	0.9
**N 1179**	1.0	1.0	1.0	1.1
**N 1180**	1.0	1.0	1.0	1.1
**N 1204**	1.1	1.3	1.6	1.2
**N 1206**	1.0	0.9	1.0	1.0
**ZS 309**	1.1	1.2	1.4	1.4
**ZS 316**	1.0	1.0	1.0	1.0
**ZS 319**	1.0	0.9	0.9	1.0
**ZS 325**	1.0	1.0	1.0	1.0
**MID 213**	1.0	1.0	1.1	1.0
**M 2767**	1.2	2.0	4.2	4.3
**M 2876**	1.0	0.9	1.0	0.8
**SH 16**	1.0	1.0	1.0	1.0
**SAI 472**	1.2	1.7	1.5	1.1
**SAI 499**	1.0	1.0	1.0	1.1
**SAI 503**	1.0	1.0	1.0	1.2
**SAI 474**	1.0	0.9	1.0	1.0
**SAI 477**	4.9	3.6	1.0	1.1
**N 1196**	1.0	1.0	1.1	1.2
**N 1197**	1.0	1.0	1.0	1.1
**SAI 127**	1.3	2.0	1.2	1.0
**N 1056**	1.0	1.0	1.1	1.1
**N 1068**	1.0	1.0	1.0	1.1
**N 1069**	1.0	1.0	1.1	1.2
**A9**	0.9	0.9	0.9	0.9
**N 1198**	1.0	1.0	1.0	0.9
**N 1193**	1.3	1.6	2.0	1.9
**N 1199**	1.0	1.1	1.3	1.1
**SAI 363**	7.5	18.8	1.3	1.0
**SAI 366**	12.1	31.0	3.5	1.0
**SAI 362**	2.8	7.7	1.4	0.9
**SAI 365**	11.8	30.9	4.1	1.0
**SAI 120**	1.0	1.0	1.1	1.0
**SAI 458**	1.1	1.5	1.1	1.0
**SAI 459**	1.0	1.0	1.0	1.0
**SAI 487**	1.0	1.0	1.0	1.0
**N 1131**	1.0	1.0	1.0	1.0
**MID 323**	1.0	1.0	1.2	1.5
**SAI 379**	1.0	1.2	1.0	0.9
**SAI 367**	1.2	1.1	1.0	1.1
**M 2738d**	1.1	2.5	3.8	1.0
**ZS 260**	1.3	1.6	1.7	1.2
**N 871a**	1.3	1.8	2.0	1.4
**N 901**	0.9	0.9	1.0	1.2

Table S2.19. Results of interaction with nanoFAST-P68N

Cmpd	Enhancement			
	430	480	530	580
**HBR-DOM2**	6.3	41.9	66.5	2.6
**HBR-2,5-DM**	1.9	6.9	5.6	0.8
**HMBR**	1.3	2.2	1.3	1.1
**HBR-DOM**	1.1	1.7	3.6	2.1
**N 871b**	1.0	1.1	1.2	1.3
**MID 145**	1.2	1.4	1.3	1.1
**N 1052**	1.2	1.6	2.0	2.3
**N 1184**	1.0	1.1	1.1	1.3
**N 1122**	1.1	1.2	1.2	1.0
**N 1049**	1.1	1.4	1.6	1.7
**N 1123**	1.0	1.0	1.1	1.0
**N 1139**	1.0	1.1	1.7	2.8
**N 1118**	1.1	1.3	1.5	1.6
**SAI 112**	1.8	5.3	1.9	1.2
**N 1135**	1.0	1.0	1.1	1.1
**SAI 118**	1.5	3.0	1.3	1.0
**N 1124**	1.0	1.0	1.0	1.1
**SAI 122**	2.4	4.0	1.2	1.0
**N 1142**	1.1	1.2	1.3	1.2
**SAI 199**	2.5	4.2	1.1	1.0
**N 967**	1.0	1.1	1.4	1.6
**N 1039**	1.2	1.4	1.6	1.5
**N 1202**	1.0	1.0	1.2	1.2
**N 960b**	1.1	1.2	1.4	1.6
**N 1036**	1.0	1.0	1.1	0.9
**SAI 117**	1.7	3.4	1.2	1.0
**N 1042**	1.0	1.0	1.0	1.2
**SAI 121**	1.2	1.8	1.2	1.1
**MID 147**	1.6	2.8	3.4	2.1
**SAI 125**	1.7	3.5	1.2	0.8
**MID 151**	1.0	1.0	1.0	1.0
**N 865**	1.0	1.0	1.1	1.3
**MID 153**	1.0	1.0	1.0	1.0
**MID 343**	1.0	1.1	1.4	1.9
**M 3007a**	1.0	1.1	1.2	1.3
**MID 367**	1.0	1.1	1.2	1.3
**N 1048**	1.1	1.2	1.4	1.7
**N 979**	1.0	1.0	1.0	0.8
**N 971**	1.0	1.0	1.0	1.0
**N 973**	1.1	1.1	1.1	1.2
**N 976**	1.0	1.0	1.0	1.0
**N 980**	1.0	1.0	1.1	1.0
**N 1205**	1.2	1.4	1.8	1.7
**N 960a**	1.0	1.1	1.2	1.1

**ZS 331**	1.1	1.3	1.4	1.5
**N 1027**	1.0	1.0	1.0	1.1
**M 2766**	1.1	1.1	1.2	1.3
**ZS 362**	1.0	1.0	1.0	1.4
**N 1179**	1.0	1.0	1.1	1.2
**N 1180**	0.9	0.9	1.0	1.1
**N 1204**	1.0	1.2	1.5	1.2
**N 1206**	1.0	1.0	1.1	1.0
**ZS 309**	1.2	1.6	2.2	1.5
**ZS 316**	1.0	1.0	1.0	1.1
**ZS 319**	1.0	0.9	0.9	1.0
**ZS 325**	1.0	1.0	1.0	1.0
**MID 213**	1.0	1.0	1.1	0.9
**M 2767**	1.1	1.5	3.0	3.1
**M 2876**	1.0	1.0	1.1	1.0
**SH 16**	1.1	1.1	1.1	1.1
**SAI 472**	1.1	1.1	1.1	0.8
**SAI 499**	1.0	1.0	1.0	1.0
**SAI 503**	1.0	1.0	1.0	1.0
**SAI 474**	1.0	1.0	1.0	0.9
**SAI 477**	1.7	1.3	1.0	1.0
**N 1196**	1.0	1.0	1.1	1.1
**N 1197**	1.0	1.0	1.1	1.0
**SAI 127**	1.8	5.0	1.7	1.2
**N 1056**	1.0	1.0	1.1	1.2
**N 1068**	1.0	1.0	1.0	1.2
**N 1069**	1.0	1.1	1.2	1.1
**A9**	1.0	1.0	1.0	1.0
**N 1198**	1.0	1.0	1.1	1.0
**N 1193**	2.4	4.0	2.5	2.0
**N 1199**	1.1	1.2	1.6	1.1
**SAI 363**	1.3	2.3	1.1	1.0
**SAI 366**	9.5	26.5	3.7	1.1
**SAI 362**	2.3	7.1	1.9	1.0
**SAI 365**	8.2	23.4	4.0	1.0
**SAI 120**	1.0	0.9	1.1	1.0
**SAI 458**	1.1	1.6	1.1	0.9
**SAI 459**	1.0	1.0	1.0	0.9
**SAI 487**	1.0	1.0	1.0	1.0
**N 1131**	1.0	1.0	1.0	1.0
**MID 323**	1.0	1.1	1.4	1.5
**SAI 379**	1.2	1.6	1.1	1.0
**SAI 367**	1.2	1.1	1.1	1.0
**M 2738d**	1.0	1.3	1.6	0.9
**ZS 260**	1.6	2.5	2.5	1.4
**N 871a**	1.5	2.6	3.0	1.7
**N 901**	1.0	1.0	1.3	1.6

Table S2.20. Results of interaction with nanoFAST-P68T

Cmpd	Enhancement			
	430	480	530	580
**HBR-DOM2**	11.8	81.6	103.8	1.4
**HBR-2,5-DM**	2.3	7.4	2.4	0.9
**HMBR**	1.4	2.3	1.1	1.2
**HBR-DOM**	1.1	1.4	2.2	1.2
**N 871b**	1.0	1.1	1.2	1.2
**MID 145**	1.2	1.4	1.3	1.0
**N 1052**	1.2	1.6	2.0	1.5
**N 1184**	1.0	1.0	1.1	0.9
**N 1122**	1.1	1.2	1.2	1.0
**N 1049**	1.1	1.3	1.7	1.5
**N 1123**	1.0	1.0	1.1	1.2
**N 1139**	1.0	1.2	2.1	2.7
**N 1118**	1.1	1.2	1.2	1.1
**SAI 112**	1.4	2.7	1.9	1.0
**N 1135**	1.0	1.0	1.1	1.0
**SAI 118**	1.1	1.2	1.1	1.0
**N 1124**	1.0	1.0	1.0	1.1
**SAI 122**	2.6	2.8	1.2	1.0
**N 1142**	1.1	1.2	1.3	1.2
**SAI 199**	3.0	3.1	1.1	1.2
**N 967**	1.0	1.0	1.3	1.6
**N 1039**	1.2	1.4	1.5	1.3
**N 1202**	1.0	1.0	1.2	1.2
**N 960b**	1.0	1.2	1.5	1.3
**N 1036**	1.0	1.0	1.0	1.1
**SAI 117**	1.5	1.9	1.2	0.8
**N 1042**	1.0	1.0	1.0	1.1
**SAI 121**	1.2	1.4	1.2	1.0
**MID 147**	1.6	2.8	3.4	1.8
**SAI 125**	1.5	2.0	1.1	1.0
**MID 151**	1.0	1.0	1.1	1.1
**N 865**	1.0	1.0	1.0	1.1
**MID 153**	1.0	1.0	1.2	1.3
**MID 343**	1.0	1.1	1.6	1.9
**M 3007a**	1.0	1.0	1.2	1.0
**MID 367**	1.0	1.1	1.2	1.2
**N 1048**	1.0	1.2	1.7	1.8
**N 979**	1.0	1.0	1.0	1.0
**N 971**	1.0	1.0	1.0	1.0
**N 973**	1.0	1.1	1.1	1.3
**N 976**	1.0	1.0	1.0	1.0
**N 980**	1.0	1.0	1.1	1.1
**N 1205**	1.3	1.5	1.7	1.4
**N 960a**	1.0	1.1	1.2	1.2

**ZS 331**	1.2	1.5	1.7	1.4
**N 1027**	1.0	1.0	1.0	1.1
**M 2766**	1.1	1.1	1.2	1.1
**ZS 362**	1.0	1.0	1.0	1.0
**N 1179**	1.0	1.0	1.1	1.1
**N 1180**	1.0	1.0	1.0	1.1
**N 1204**	1.2	1.4	1.4	1.3
**N 1206**	1.2	1.4	1.1	1.0
**ZS 309**	1.2	1.8	2.6	1.6
**ZS 316**	1.0	1.0	1.0	1.0
**ZS 319**	1.0	1.0	1.0	1.0
**ZS 325**	1.0	1.0	1.1	1.2
**MID 213**	1.0	1.1	1.1	1.1
**M 2767**	1.2	2.0	3.6	3.5
**M 2876**	1.2	1.1	1.1	1.2
**SH 16**	1.1	1.1	1.1	1.1
**SAI 472**	1.2	1.3	1.1	1.1
**SAI 499**	1.0	1.0	1.0	1.0
**SAI 503**	1.0	1.0	1.0	1.0
**SAI 474**	1.1	1.0	1.1	0.8
**SAI 477**	3.9	1.8	1.0	1.0
**N 1196**	1.0	1.0	1.2	1.0
**N 1197**	1.0	1.0	1.0	1.1
**SAI 127**	1.8	3.1	1.5	1.0
**N 1056**	1.0	1.0	1.2	1.2
**N 1068**	1.0	1.0	1.0	1.0
**N 1069**	1.0	1.1	1.2	1.1
**A9**	1.0	1.0	0.9	1.1
**N 1198**	1.0	1.0	1.1	1.0
**N 1193**	2.6	3.9	2.5	2.4
**N 1199**	1.1	1.2	1.6	1.5
**SAI 363**	1.9	3.2	1.1	0.9
**SAI 366**	11.4	30.2	2.2	1.1
**SAI 362**	4.9	12.8	1.5	1.0
**SAI 365**	11.1	29.8	2.6	0.9
**SAI 120**	1.1	1.1	1.1	0.9
**SAI 458**	1.4	1.9	1.0	1.0
**SAI 459**	1.0	1.0	1.0	1.0
**SAI 487**	1.0	1.0	1.0	1.0
**N 1131**	1.0	1.0	1.0	1.1
**MID 323**	1.0	1.1	1.5	2.0
**SAI 379**	1.2	1.3	1.1	1.0
**SAI 367**	1.3	1.1	1.1	1.0
**M 2738d**	1.1	1.4	1.9	1.1
**ZS 260**	1.6	2.7	2.8	1.5
**N 871a**	1.6	2.5	2.8	1.7
**N 901**	1.0	1.1	1.3	1.5

Table S2.21. Results of interaction with nanoFAST-P68Q

Cmpd	Enhancement			
	430	480	530	580
**HBR-DOM2**	14.5	87.7	135.6	2.0
**HBR-2,5-DM**	2.9	10.2	3.3	1.1
**HMBR**	1.4	2.1	1.2	0.9
**HBR-DOM**	1.0	1.3	1.9	1.3
**N 871b**	1.0	1.1	1.4	1.3
**MID 145**	1.2	1.5	1.5	1.2
**N 1052**	1.2	1.6	2.1	2.3
**N 1184**	1.0	1.2	1.5	1.2
**N 1122**	1.0	0.8	1.1	1.2
**N 1049**	1.1	1.4	1.5	1.7
**N 1123**	1.0	1.0	1.0	1.0
**N 1139**	1.0	1.2	1.8	2.9
**N 1118**	1.1	1.1	1.1	1.4
**SAI 112**	1.4	2.5	1.8	0.9
**N 1135**	1.0	1.0	1.0	0.7
**SAI 118**	1.0	1.2	1.1	0.8
**N 1124**	1.0	1.0	1.0	1.2
**SAI 122**	3.1	3.5	1.2	1.0
**N 1142**	1.1	1.2	1.2	1.0
**SAI 199**	3.5	3.8	1.0	1.1
**N 967**	1.0	1.1	1.4	1.5
**N 1039**	1.2	1.6	1.9	1.3
**N 1202**	1.0	1.1	1.4	1.4
**N 960b**	1.0	1.2	1.5	1.7
**N 1036**	1.0	1.0	1.1	1.1
**SAI 117**	1.2	1.6	1.1	0.9
**N 1042**	1.0	1.0	1.0	0.9
**SAI 121**	1.2	1.3	1.2	1.0
**MID 147**	1.5	2.8	3.9	2.5
**SAI 125**	1.3	1.8	1.1	1.2
**MID 151**	1.0	1.0	1.0	0.9
**N 865**	1.0	1.0	1.0	1.0
**MID 153**	1.0	1.0	1.0	0.9
**MID 343**	1.0	1.1	1.6	2.9
**M 3007a**	1.0	1.1	1.2	1.1
**MID 367**	1.0	1.1	1.2	1.5
**N 1048**	1.0	1.2	1.8	2.3
**N 979**	1.0	1.0	1.1	1.3
**N 971**	1.0	1.0	1.0	0.9
**N 973**	1.0	1.1	1.1	1.2
**N 976**	1.0	1.0	1.0	1.0
**N 980**	1.0	1.0	1.1	0.9
**N 1205**	1.2	1.5	2.0	1.7
**N 960a**	1.0	1.0	1.1	1.0

**ZS 331**	1.2	1.4	1.7	2.0
**N 1027**	1.0	1.0	1.0	1.2
**M 2766**	1.0	1.2	1.3	1.1
**ZS 362**	1.0	1.0	1.0	1.0
**N 1179**	1.1	1.0	1.0	1.0
**N 1180**	1.1	1.0	1.0	0.9
**N 1204**	1.2	1.4	1.7	1.4
**N 1206**	1.1	1.3	1.1	1.0
**ZS 309**	1.3	1.8	2.5	1.7
**ZS 316**	1.1	1.0	1.0	1.1
**ZS 319**	1.1	1.0	1.1	1.0
**ZS 325**	1.1	1.0	1.0	1.0
**MID 213**	1.1	1.1	1.2	1.2
**M 2767**	1.2	1.6	2.1	2.1
**M 2876**	1.0	1.0	1.0	1.1
**SH 16**	1.2	1.2	1.2	1.1
**SAI 472**	1.2	1.3	1.1	1.0
**SAI 499**	1.1	1.0	1.1	0.9
**SAI 503**	1.1	1.0	1.0	0.9
**SAI 474**	1.1	1.0	1.0	1.1
**SAI 477**	2.7	1.5	1.0	0.9
**N 1196**	1.1	1.1	1.1	1.1
**N 1197**	1.1	1.0	1.1	1.0
**SAI 127**	1.7	2.9	1.4	1.0
**N 1056**	1.0	1.1	1.1	1.2
**N 1068**	1.1	1.1	1.1	1.2
**N 1069**	1.1	1.2	1.3	1.1
**A9**	1.1	1.0	1.0	1.0
**N 1198**	1.1	1.1	1.1	1.0
**N 1193**	1.8	2.6	2.1	2.2
**N 1199**	1.1	1.2	1.5	1.3
**SAI 363**	1.9	3.4	1.1	1.0
**SAI 366**	12.5	37.0	2.7	1.1
**SAI 362**	4.1	12.2	1.6	1.0
**SAI 365**	11.8	35.6	3.2	0.9
**SAI 120**	1.1	1.1	1.1	1.1
**SAI 458**	1.4	2.1	1.0	1.1
**SAI 459**	1.0	1.0	1.0	1.0
**SAI 487**	1.1	1.0	1.1	1.1
**N 1131**	1.1	1.1	1.0	1.1
**MID 323**	1.1	1.1	1.4	1.6
**SAI 379**	1.2	1.3	1.1	1.0
**SAI 367**	1.2	1.1	1.1	1.1
**M 2738d**	1.1	1.5	2.1	1.1
**ZS 260**	1.5	2.3	2.5	1.5
**N 871a**	1.5	2.5	3.2	2.0
**N 901**	1.1	1.1	1.3	1.5

Table S2.22. Results of interaction with nanoFAST-P68S

Cmpd	Enhancement			
	430	480	530	580
**HBR-DOM2**	10.5	70.1	96.7	1.4
**HBR-2,5-DM**	2.1	6.3	2.2	1.0
**HMBR**	1.3	1.8	1.1	1.0
**HBR-DOM**	1.1	1.4	2.0	1.2
**N 871b**	1.1	1.1	1.3	1.2
**MID 145**	1.1	1.2	1.2	1.0
**N 1052**	1.2	1.3	1.4	1.3
**N 1184**	1.1	1.1	1.3	1.1
**N 1122**	1.1	1.2	1.1	1.1
**N 1049**	1.1	1.1	1.2	1.1
**N 1123**	1.1	1.0	1.1	1.0
**N 1139**	1.1	1.1	1.8	2.1
**N 1118**	1.1	1.2	1.2	1.0
**SAI 112**	1.5	2.4	1.7	1.1
**N 1135**	1.1	1.0	1.0	1.1
**SAI 118**	1.2	1.2	1.1	1.0
**N 1124**	1.1	1.1	1.1	1.1
**SAI 122**	2.5	2.7	1.2	1.1
**N 1142**	1.1	1.1	1.1	1.0
**SAI 199**	2.6	2.6	1.0	1.1
**N 967**	1.1	1.1	1.2	1.2
**N 1039**	1.2	1.3	1.4	1.2
**N 1202**	1.1	1.1	1.3	1.2
**N 960b**	1.1	1.1	1.2	1.3
**N 1036**	1.1	1.1	1.1	1.1
**SAI 117**	1.3	1.6	1.2	0.9
**N 1042**	1.1	1.0	1.0	1.1
**SAI 121**	1.2	1.3	1.2	1.0
**MID 147**	1.3	1.5	1.8	1.4
**SAI 125**	1.4	1.7	1.1	1.1
**MID 151**	1.1	1.1	1.1	1.2
**N 865**	1.1	1.0	1.1	1.0
**MID 153**	1.1	1.1	1.2	1.2
**MID 343**	1.1	1.1	1.4	1.4
**M 3007a**	1.1	1.1	1.2	1.0
**MID 367**	1.1	1.1	1.1	1.1
**N 1048**	1.1	1.2	1.7	1.7
**N 979**	1.1	1.0	1.1	1.0
**N 971**	1.1	1.0	1.0	1.1
**N 973**	1.1	1.0	1.1	1.1
**N 976**	1.1	1.0	1.0	1.2
**N 980**	1.1	1.0	1.1	0.9
**N 1205**	1.2	1.4	1.6	1.2
**N 960a**	1.1	1.1	1.1	1.0

**ZS 331**	1.1	1.2	1.3	1.2
**N 1027**	1.1	1.0	1.0	1.0
**M 2766**	1.1	1.1	1.2	1.0
**ZS 362**	1.1	1.0	1.0	1.0
**N 1179**	1.1	1.1	1.0	1.0
**N 1180**	1.1	1.1	1.1	1.0
**N 1204**	1.2	1.3	1.4	1.1
**N 1206**	1.1	1.2	1.1	1.1
**ZS 309**	1.3	1.6	1.7	1.2
**ZS 316**	1.1	1.0	1.0	0.9
**ZS 319**	1.0	0.9	0.8	1.0
**ZS 325**	1.1	1.0	1.0	1.0
**MID 213**	1.1	1.1	1.1	1.1
**M 2767**	1.2	1.5	2.0	2.0
**M 2876**	1.1	1.0	1.0	1.0
**SH 16**	1.2	1.2	1.2	1.1
**SAI 472**	1.2	1.2	1.1	1.1
**SAI 499**	1.1	1.0	1.0	1.2
**SAI 503**	1.1	1.0	1.1	1.1
**SAI 474**	1.1	1.0	1.0	1.0
**SAI 477**	2.2	1.5	1.0	1.0
**N 1196**	1.1	1.1	1.1	1.1
**N 1197**	1.1	1.0	1.1	1.0
**SAI 127**	1.5	2.4	1.4	1.1
**N 1056**	1.1	1.0	1.1	1.2
**N 1068**	1.1	1.1	1.1	1.0
**N 1069**	1.1	1.2	1.3	1.1
**A9**	1.1	1.0	1.0	1.0
**N 1198**	1.1	1.0	1.0	1.1
**N 1193**	2.3	3.2	2.3	2.2
**N 1199**	1.1	1.2	1.6	1.3
**SAI 363**	1.6	2.8	1.1	1.1
**SAI 366**	10.7	29.2	2.3	1.0
**SAI 362**	3.1	8.3	1.4	1.0
**SAI 365**	9.6	26.7	2.5	1.0
**SAI 120**	1.1	1.1	1.1	1.1
**SAI 458**	1.3	1.9	1.0	1.0
**SAI 459**	1.1	1.0	1.0	1.0
**SAI 487**	1.1	1.0	1.0	1.1
**N 1131**	1.1	1.1	1.0	1.1
**MID 323**	1.1	1.1	1.4	1.5
**SAI 379**	1.2	1.3	1.1	1.1
**SAI 367**	1.2	1.1	1.1	1.0
**M 2738d**	1.1	1.5	1.9	1.0
**ZS 260**	1.5	2.3	2.4	1.5
**N 871a**	1.5	2.1	2.1	1.2
**N 901**	0.9	1.1	1.2	1.4

Table S2.23. Results of interaction with nanoFAST-G69S

Cmpd	Enhancement			
	430	480	530	580
**HBR-DOM2**	3.3	19.7	40.6	1.3
**HBR-2,5-DM**	1.3	2.6	1.6	1.0
**HMBR**	1.1	1.2	1.1	1.0
**HBR-DOM**	1.1	1.2	1.6	1.2
**N 871b**	1.1	1.1	1.1	1.1
**MID 145**	1.1	1.1	1.1	1.1
**N 1052**	1.1	1.2	1.2	1.2
**N 1184**	1.1	1.1	1.1	1.0
**N 1122**	1.1	1.1	1.2	1.1
**N 1049**	1.1	1.1	1.2	1.1
**N 1123**	1.1	1.0	1.1	1.1
**N 1139**	1.1	1.3	2.7	4.5
**N 1118**	1.1	1.2	1.1	1.0
**SAI 112**	1.5	2.0	1.7	1.0
**N 1135**	1.1	1.0	1.1	1.2
**SAI 118**	1.1	1.3	1.4	1.1
**N 1124**	1.1	1.1	1.1	1.1
**SAI 122**	2.0	2.6	1.2	1.3
**N 1142**	1.1	1.1	1.2	1.0
**SAI 199**	1.9	2.4	1.1	1.0
**N 967**	1.1	1.0	1.1	1.1
**N 1039**	1.1	1.2	1.2	1.2
**N 1202**	1.1	1.0	1.0	1.0
**N 960b**	1.1	1.2	1.2	1.3
**N 1036**	1.1	1.0	1.0	1.1
**SAI 117**	1.2	1.4	1.3	1.0
**N 1042**	1.1	1.0	1.0	1.1
**SAI 121**	1.2	1.3	1.2	0.9
**MID 147**	1.3	1.4	1.6	1.3
**SAI 125**	1.2	1.4	1.2	1.1
**MID 151**	1.1	1.1	1.2	1.3
**N 865**	1.1	1.0	1.0	0.9
**MID 153**	1.1	1.1	1.2	1.2
**MID 343**	1.1	1.1	1.2	1.6
**M 3007a**	1.1	1.1	1.1	1.0
**MID 367**	1.1	1.0	1.1	1.1
**N 1048**	1.1	1.2	1.3	1.2
**N 979**	1.1	1.0	1.1	0.9
**N 971**	1.1	1.1	1.0	1.0
**N 973**	1.1	1.1	1.1	1.2
**N 976**	1.1	1.0	1.0	1.1
**N 980**	1.1	1.0	1.1	1.0
**N 1205**	1.3	1.5	1.8	1.5
**N 960a**	1.1	1.1	1.1	1.1

**ZS 331**	1.2	1.3	1.3	1.3
**N 1027**	1.1	1.0	1.0	1.0
**M 2766**	1.1	1.1	1.1	1.1
**ZS 362**	1.1	1.0	1.0	1.0
**N 1179**	1.1	1.1	1.1	1.0
**N 1180**	1.1	1.1	1.1	1.1
**N 1204**	1.3	1.4	1.4	1.6
**N 1206**	1.1	1.0	1.0	1.1
**ZS 309**	1.3	1.6	2.2	2.0
**ZS 316**	1.1	1.0	1.0	1.2
**ZS 319**	1.1	1.0	1.1	1.0
**ZS 325**	1.1	1.1	1.0	0.9
**MID 213**	1.1	1.1	1.1	1.0
**M 2767**	1.3	1.9	2.8	2.3
**M 2876**	1.1	1.0	1.1	1.0
**SH 16**	1.1	1.1	1.1	1.2
**SAI 472**	1.1	1.1	1.1	1.0
**SAI 499**	1.1	1.1	1.0	0.9
**SAI 503**	1.1	1.0	1.1	1.4
**SAI 474**	1.1	1.0	1.0	1.0
**SAI 477**	1.7	1.4	1.0	0.9
**N 1196**	1.1	1.1	1.1	1.0
**N 1197**	1.1	1.1	1.1	0.9
**SAI 127**	1.4	1.8	1.6	1.1
**N 1056**	1.1	1.1	1.1	1.5
**N 1068**	1.1	1.1	1.1	1.2
**N 1069**	1.1	1.3	1.3	1.2
**A9**	1.1	1.0	1.0	1.0
**N 1198**	1.1	1.1	1.0	1.2
**N 1193**	1.5	1.9	2.0	2.4
**N 1199**	1.1	1.2	1.5	1.3
**SAI 363**	1.4	2.3	1.1	1.1
**SAI 366**	8.3	25.1	3.2	1.1
**SAI 362**	2.1	5.4	1.4	0.9
**SAI 365**	6.2	18.6	3.2	1.0
**SAI 120**	1.2	1.2	1.2	1.0
**SAI 458**	1.2	1.3	1.1	1.0
**SAI 459**	1.1	1.0	1.0	0.9
**SAI 487**	1.1	1.0	1.0	1.1
**N 1131**	1.1	1.1	1.0	1.1
**MID 323**	1.1	1.1	1.4	1.9
**SAI 379**	1.1	1.2	1.2	1.0
**SAI 367**	1.2	1.2	1.1	1.0
**M 2738d**	1.1	1.3	1.7	1.2
**ZS 260**	1.4	2.2	2.3	1.4
**N 871a**	1.5	2.0	2.3	1.7
**N 901**	1.1	1.1	1.3	1.7

Table S2.24. Results of interaction with nanoFAST-G69T

Cmpd	Enhancement			
	430	480	530	580
**HBR-DOM2**	5.2	41.2	73.9	1.9
**HBR-2,5-DM**	1.4	4.1	2.0	1.1
**HMBR**	1.1	1.4	1.1	1.3
**HBR-DOM**	1.0	1.2	1.6	1.3
**N 871b**	1.0	1.0	1.0	1.0
**MID 145**	1.1	1.3	1.2	0.8
**N 1052**	1.1	1.4	1.7	1.6
**N 1184**	1.0	1.0	1.0	0.9
**N 1122**	1.0	1.1	1.1	1.1
**N 1049**	1.1	1.3	1.5	1.2
**N 1123**	1.0	1.0	1.1	1.7
**N 1139**	1.0	1.2	2.4	4.1
**N 1118**	1.0	1.1	1.1	1.5
**SAI 112**	1.2	1.9	1.7	1.2
**N 1135**	1.0	1.0	1.1	1.4
**SAI 118**	1.0	1.1	1.1	0.9
**N 1124**	1.0	1.0	1.0	1.2
**SAI 122**	2.3	2.9	1.2	1.0
**N 1142**	1.1	1.2	1.2	1.4
**SAI 199**	2.3	2.9	1.0	0.9
**N 967**	1.0	1.0	1.2	1.4
**N 1039**	1.2	1.3	1.3	1.3
**N 1202**	0.9	0.9	1.0	1.3
**N 960b**	1.1	1.2	1.3	1.4
**N 1036**	1.0	1.0	1.1	1.3
**SAI 117**	1.1	1.3	1.1	1.5
**N 1042**	1.0	1.0	1.0	1.2
**SAI 121**	1.1	1.3	1.2	1.2
**MID 147**	1.4	2.4	2.7	1.6
**SAI 125**	1.1	1.3	1.1	0.8
**MID 151**	1.0	1.0	1.0	0.9
**N 865**	1.0	1.0	1.0	0.9
**MID 153**	1.0	1.0	0.9	0.9
**MID 343**	1.0	1.0	1.3	1.6
**M 3007a**	1.0	1.0	1.2	1.1
**MID 367**	1.0	1.0	1.2	1.6
**N 1048**	1.0	1.1	1.1	1.0
**N 979**	1.0	1.0	1.0	1.2
**N 971**	1.0	1.0	1.0	1.1
**N 973**	1.0	1.1	1.0	0.9
**N 976**	1.0	1.0	1.0	0.6
**N 980**	1.0	1.0	1.1	0.8
**N 1205**	1.4	1.6	1.6	1.6
**N 960a**	1.0	1.1	1.2	1.1

**ZS 331**	1.1	1.3	1.1	1.0
**N 1027**	1.0	1.0	1.0	1.0
**M 2766**	1.0	1.1	1.0	1.2
**ZS 362**	1.0	1.0	1.0	0.9
**N 1179**	1.0	1.0	1.0	1.0
**N 1180**	1.0	1.0	1.0	1.1
**N 1204**	1.2	1.3	1.4	1.4
**N 1206**	1.0	1.0	1.0	1.1
**ZS 309**	1.2	1.6	2.2	1.6
**ZS 316**	1.0	1.0	1.0	1.0
**ZS 319**	1.0	1.0	0.9	0.9
**ZS 325**	1.0	1.0	1.0	1.1
**MID 213**	1.0	1.0	1.1	1.2
**M 2767**	1.4	2.7	5.8	3.5
**M 2876**	1.0	1.0	1.0	0.9
**SH 16**	1.1	1.0	1.1	0.9
**SAI 472**	1.1	1.2	1.1	1.2
**SAI 499**	1.0	1.0	1.0	1.1
**SAI 503**	1.0	1.0	1.0	1.0
**SAI 474**	1.1	1.0	1.0	1.0
**SAI 477**	1.9	1.5	1.0	1.0
**N 1196**	1.0	1.0	1.1	1.0
**N 1197**	1.0	1.0	1.0	1.1
**SAI 127**	1.1	1.5	1.3	1.0
**N 1056**	1.1	1.0	1.1	1.3
**N 1068**	1.0	1.0	1.0	1.1
**N 1069**	1.0	1.2	1.4	1.0
**A9**	1.0	1.0	1.0	1.0
**N 1198**	1.0	1.1	1.1	0.9
**N 1193**	1.5	1.9	2.0	2.0
**N 1199**	1.1	1.2	1.5	1.2
**SAI 363**	1.4	2.6	1.1	0.9
**SAI 366**	9.9	29.2	3.7	1.0
**SAI 362**	2.6	7.5	1.5	1.1
**SAI 365**	7.4	22.0	3.5	1.2
**SAI 120**	1.1	1.1	1.1	1.1
**SAI 458**	1.2	1.6	1.0	1.0
**SAI 459**	1.0	1.0	1.0	0.9
**SAI 487**	1.0	1.0	1.0	1.0
**N 1131**	1.0	1.0	1.0	0.9
**MID 323**	1.0	1.1	1.7	2.0
**SAI 379**	1.0	1.1	1.1	0.8
**SAI 367**	1.1	1.0	0.9	1.0
**M 2738d**	1.0	1.2	1.5	1.0
**ZS 260**	1.4	2.2	2.2	1.3
**N 871a**	1.4	2.2	2.5	1.4
**N 901**	1.0	1.0	1.1	1.3

Table S2.25. Results of interaction with nanoFAST-G69N

Cmpd	Enhancement			
	430	480	530	580
HBR-DOM2	5.0	39.9	66.2	1.9
HBR-2,5-DM	1.4	3.5	1.5	1.0
HMBR	1.2	1.3	1.0	1.2
HBR-DOM	1.0	1.2	1.6	1.2
N 871b	1.0	1.0	1.0	1.1
MID 145	1.1	1.1	1.1	0.9
N 1052	1.1	1.3	1.4	1.2
N 1184	1.0	1.0	1.0	1.0
N 1122	1.1	1.1	1.1	0.9
N 1049	1.0	1.1	1.2	1.3
N 1123	1.0	1.0	1.0	1.1
N 1139	1.0	1.2	2.5	4.6
N 1118	1.0	1.1	1.1	1.2
SAI 112	1.4	2.4	2.0	1.2
N 1135	1.0	1.0	1.0	1.4
SAI 118	1.1	1.2	1.1	1.0
N 1124	1.0	1.0	1.1	1.2
SAI 122	2.2	2.4	1.2	1.0
N 1142	1.1	1.1	1.1	1.4
SAI 199	2.3	2.6	1.0	1.0
N 967	1.0	1.1	1.3	1.6
N 1039	1.1	1.2	1.2	1.2
N 1202	1.0	1.0	1.0	1.2
N 960b	1.0	1.2	1.4	1.7
N 1036	1.0	1.0	1.0	0.8
SAI 117	1.1	1.4	1.2	1.1
N 1042	1.0	1.0	1.0	0.9
SAI 121	1.1	1.2	1.2	1.1
MID 147	1.4	2.0	2.5	1.9
SAI 125	1.2	1.4	1.2	0.9
MID 151	1.0	1.1	1.1	1.1
N 865	1.0	1.0	1.0	0.8
MID 153	1.0	1.0	1.1	1.2
MID 343	1.0	1.0	1.2	1.6
M 3007a	1.0	1.0	1.1	1.2
MID 367	1.0	1.1	1.2	1.4
N 1048	1.0	1.1	1.2	1.3
N 979	1.0	1.0	1.0	1.0
N 971	1.0	1.0	1.0	1.1
N 973	1.0	1.1	1.1	1.3
N 976	1.0	1.0	1.0	0.9
N 980	1.0	1.0	1.1	1.1
N 1205	1.6	1.7	1.4	1.6
N 960a	1.0	1.1	1.2	1.0

**ZS 331**	1.1	1.3	1.4	1.2
**N 1027**	1.0	1.0	1.0	0.9
**M 2766**	1.0	1.1	1.1	1.2
**ZS 362**	1.0	1.0	1.0	0.8
**N 1179**	1.0	1.0	1.0	0.9
**N 1180**	1.0	1.0	1.0	1.0
**N 1204**	1.3	1.3	1.2	1.3
**N 1206**	1.0	1.0	1.0	1.0
**ZS 309**	1.1	1.5	2.5	2.0
**ZS 316**	1.0	1.0	1.0	1.0
**ZS 319**	1.0	1.0	1.0	1.0
**ZS 325**	1.0	1.0	1.0	1.0
**MID 213**	1.0	1.1	1.1	1.1
**M 2767**	1.1	1.5	2.3	2.3
**M 2876**	1.0	1.0	1.0	1.0
**SH 16**	1.0	1.0	1.1	1.0
**SAI 472**	1.0	1.1	1.1	0.9
**SAI 499**	1.0	1.0	1.0	0.9
**SAI 503**	1.0	1.0	1.0	1.0
**SAI 474**	1.1	1.0	1.0	1.0
**SAI 477**	1.7	1.3	1.0	0.9
**N 1196**	1.0	1.0	1.1	1.0
**N 1197**	1.0	1.0	1.0	0.9
**SAI 127**	1.1	1.7	1.4	1.0
**N 1056**	1.0	1.0	1.0	1.2
**N 1068**	1.0	1.0	1.0	0.9
**N 1069**	1.0	1.3	1.3	1.1
**A9**	1.0	1.0	1.0	0.8
**N 1198**	1.0	1.0	1.1	1.1
**N 1193**	1.5	2.1	2.0	2.3
**N 1199**	1.1	1.2	1.4	1.2
**SAI 363**	1.2	1.7	1.1	1.0
**SAI 366**	6.0	15.9	2.1	0.9
**SAI 362**	2.0	4.8	1.3	0.9
**SAI 365**	4.9	13.2	2.2	1.0
**SAI 120**	1.1	1.1	1.1	1.1
**SAI 458**	1.1	1.3	1.1	1.0
**SAI 459**	1.0	1.0	1.0	1.0
**SAI 487**	1.0	1.0	1.0	1.2
**N 1131**	1.0	1.0	1.0	1.0
**MID 323**	1.0	1.1	1.3	2.0
**SAI 379**	1.0	1.1	1.2	1.0
**SAI 367**	1.3	1.1	1.1	1.1
**M 2738d**	1.0	1.4	1.8	1.1
**ZS 260**	1.5	2.4	2.4	1.4
**N 871a**	1.4	2.0	2.3	1.6
**N 901**	1.0	1.0	1.1	1.5

Table S2.26. Results of interaction with nanoFAST-G69Q

Cmpd	Enhancement			
	430	480	530	580
HBR-DOM2	5.0	39.9	66.2	1.9
HBR-2,5-DM	1.4	3.5	1.5	1.0
HMBR	1.2	1.3	1.0	1.2
HBR-DOM	1.0	1.2	1.6	1.2
N 871b	1.0	1.0	1.0	1.1
MID 145	1.1	1.1	1.1	0.9
N 1052	1.1	1.3	1.4	1.2
N 1184	1.0	1.0	1.0	1.0
N 1122	1.1	1.1	1.1	0.9
N 1049	1.0	1.1	1.2	1.3
N 1123	1.0	1.0	1.0	1.1
N 1139	1.0	1.2	2.5	4.6
N 1118	1.0	1.1	1.1	1.2
SAI 112	1.4	2.4	2.0	1.2
N 1135	1.0	1.0	1.0	1.4
SAI 118	1.1	1.2	1.1	1.0
N 1124	1.0	1.0	1.1	1.2
SAI 122	2.2	2.4	1.2	1.0
N 1142	1.1	1.1	1.1	1.4
SAI 199	2.3	2.6	1.0	1.0
N 967	1.0	1.1	1.3	1.6
N 1039	1.1	1.2	1.2	1.2
N 1202	1.0	1.0	1.0	1.2
N 960b	1.0	1.2	1.4	1.7
N 1036	1.0	1.0	1.0	0.8
SAI 117	1.1	1.4	1.2	1.1
N 1042	1.0	1.0	1.0	0.9
SAI 121	1.1	1.2	1.2	1.1
MID 147	1.4	2.0	2.5	1.9
SAI 125	1.2	1.4	1.2	0.9
MID 151	1.0	1.1	1.1	1.1
N 865	1.0	1.0	1.0	0.8
MID 153	1.0	1.0	1.1	1.2
MID 343	1.0	1.0	1.2	1.6
M 3007a	1.0	1.0	1.1	1.2
MID 367	1.0	1.1	1.2	1.4
N 1048	1.0	1.1	1.2	1.3
N 979	1.0	1.0	1.0	1.0
N 971	1.0	1.0	1.0	1.1
N 973	1.0	1.1	1.1	1.3
N 976	1.0	1.0	1.0	0.9
N 980	1.0	1.0	1.1	1.1
N 1205	1.6	1.7	1.4	1.6
N 960a	1.0	1.1	1.2	1.0

**ZS 331**	1.1	1.3	1.4	1.2
**N 1027**	1.0	1.0	1.0	0.9
**M 2766**	1.0	1.1	1.1	1.2
**ZS 362**	1.0	1.0	1.0	0.8
**N 1179**	1.0	1.1	1.0	0.9
**N 1180**	1.0	1.0	1.1	1.0
**N 1204**	1.2	1.3	1.2	1.3
**N 1206**	1.0	1.0	1.0	1.1
**ZS 309**	1.2	1.7	2.5	2.4
**ZS 316**	1.0	1.0	1.0	1.1
**ZS 319**	1.0	1.0	1.0	1.1
**ZS 325**	1.0	1.0	1.0	0.9
**MID 213**	1.0	1.0	1.2	1.2
**M 2767**	1.3	2.7	5.5	3.8
**M 2876**	1.0	1.0	1.0	1.1
**SH 16**	1.0	1.0	1.1	0.9
**SAI 472**	1.1	1.1	1.1	0.9
**SAI 499**	1.0	1.0	1.0	1.1
**SAI 503**	1.0	1.0	1.0	1.1
**SAI 474**	1.0	1.0	1.0	1.1
**SAI 477**	1.2	1.1	1.0	1.0
**N 1196**	1.0	1.1	1.1	1.0
**N 1197**	1.0	1.0	1.0	1.1
**SAI 127**	1.2	1.7	1.4	0.9
**N 1056**	1.0	1.1	1.2	1.2
**N 1068**	1.0	1.0	1.0	0.9
**N 1069**	1.0	1.2	1.3	0.9
**A9**	1.0	1.0	1.0	1.1
**N 1198**	1.0	1.0	1.1	0.9
**N 1193**	1.7	2.3	1.8	1.8
**N 1199**	1.1	1.2	1.4	1.1
**SAI 363**	1.2	1.6	1.1	1.0
**SAI 366**	6.7	15.3	1.7	1.0
**SAI 362**	1.4	2.4	1.1	1.0
**SAI 365**	5.2	12.1	1.8	1.1
**SAI 120**	1.1	1.1	1.1	1.2
**SAI 458**	1.0	1.1	1.0	0.7
**SAI 459**	1.0	1.0	1.0	0.7
**SAI 487**	1.0	1.0	1.0	0.9
**N 1131**	1.0	1.0	1.0	0.9
**MID 323**	1.0	1.1	1.4	1.8
**SAI 379**	1.0	1.1	1.1	1.1
**SAI 367**	1.2	1.1	1.1	0.9
**M 2738d**	1.0	1.3	1.5	1.0
**ZS 260**	1.6	2.6	3.0	1.6
**N 871a**	1.5	2.3	2.7	1.8
**N 901**	1.0	1.0	1.1	1.7

Table S2.27. Results of interaction with nanoFAST-E46Q

Cmpd	Enhancement			
	430	480	530	580
HBR-DOM2	13.0	>145.6	>354.5	11.5
HBR-2,5-DM	12.9	121.0	130.5	1.1
HMBR	10.6	54.3	10.7	1.1
HBR-DOM	4.3	38.8	171.0	42.2
N 871b	1.6	5.9	19.5	27.8
MID 145	1.8	7.9	29.5	2.5
N 1052	1.6	4.0	15.9	15.7
N 1184	1.8	7.9	25.9	13.9
N 1122	1.6	4.7	6.1	1.4
N 1049	1.5	5.6	23.5	12.8
N 1123	1.1	1.1	2.3	3.5
N 1139	1.1	1.6	5.0	8.8
N 1118	1.9	6.8	11.3	1.5
SAI 112	12.2	>67.1	4.2	1.0
N 1135	1.1	1.2	1.4	1.7
SAI 118	11.9	41.0	2.7	1.1
N 1124	1.1	2.0	4.2	2.6
SAI 122	8.6	25.9	1.6	1.1
N 1142	1.1	1.3	2.0	1.4
SAI 199	16.9	54.8	1.8	1.0
N 967	1.2	2.7	10.4	20.5
N 1039	1.7	5.1	19.0	11.8
N 1202	1.7	7.0	23.3	24.3
N 960b	1.2	2.6	10.5	26.5
N 1036	1.9	6.6	1.9	1.4
SAI 117	14.8	53.1	2.7	1.0
N 1042	1.1	1.0	1.0	1.1
SAI 121	8.6	27.7	1.8	1.1
MID 147	2.5	5.0	14.1	21.4
SAI 125	16.5	62.2	3.0	1.1
MID 151	1.1	1.7	4.3	9.3
N 865	1.2	1.2	1.2	1.1
MID 153	1.1	1.3	2.7	7.3
MID 343	1.3	1.5	6.7	18.1
M 3007a	1.1	1.2	2.7	3.3
MID 367	1.2	1.2	4.2	14.4
N 1048	1.2	1.9	7.9	17.8
N 979	1.1	1.1	1.2	1.0
N 971	1.1	1.6	4.3	6.6
N 973	1.1	1.6	4.1	5.5
N 976	1.1	1.2	1.3	1.5
N 980	1.1	1.1	1.1	1.2
N 1205	2.2	8.4	27.0	18.4
N 960a	1.1	1.1	1.2	1.4

**ZS 331**	1.3	2.4	8.4	16.2
**N 1027**	2.4	1.1	1.1	1.1
**M 2766**	1.7	6.3	21.1	16.8
**ZS 362**	1.2	1.1	1.0	1.2
**N 1179**	1.2	1.1	1.1	1.0
**N 1180**	1.2	1.1	1.1	0.9
**N 1204**	2.0	7.4	21.5	14.0
**N 1206**	1.3	3.2	19.9	57.8
**ZS 309**	1.4	2.2	3.4	3.3
**ZS 316**	1.1	1.9	3.9	1.4
**ZS 319**	1.1	1.1	1.0	1.0
**ZS 325**	1.1	1.1	1.1	1.0
**MID 213**	1.3	2.3	10.3	23.0
**M 2767**	1.3	2.1	4.6	9.7
**M 2876**	1.2	1.5	1.0	1.3
**SH 16**	1.5	4.1	6.2	1.6
**SAI 472**	2.9	7.6	4.8	1.4
**SAI 499**	1.6	1.7	1.1	1.0
**SAI 503**	2.3	1.3	1.0	1.0
**SAI 474**	2.0	1.0	1.0	0.9
**SAI 477**	11.7	7.4	1.1	0.9
**N 1196**	1.1	1.1	1.1	0.9
**N 1197**	1.1	1.1	1.0	1.0
**SAI 127**	12.9	59.7	3.2	1.0
**N 1056**	1.1	1.2	1.8	3.3
**N 1068**	1.1	1.0	1.1	1.1
**N 1069**	1.1	1.1	1.2	1.2
**A9**	1.1	1.0	1.1	1.1
**N 1198**	1.1	1.0	1.2	1.2
**N 1193**	1.8	2.7	4.2	7.1
**N 1199**	1.2	1.6	3.1	2.6
**SAI 363**	7.5	30.2	1.8	0.9
**SAI 366**	19.7	>93.8	28.2	1.1
**SAI 362**	15.4	87.4	14.7	1.0
**SAI 365**	15.9	>87.3	27.4	0.9
**SAI 120**	1.1	1.2	1.2	1.1
**SAI 458**	7.0	31.6	5.7	1.2
**SAI 459**	1.1	1.1	1.0	1.2
**SAI 487**	1.1	1.0	1.0	0.9
**N 1131**	1.3	3.1	1.6	1.0
**MID 323**	1.1	1.2	2.2	4.0
**SAI 379**	6.8	11.0	1.2	1.3
**SAI 367**	1.6	2.3	1.7	1.2
**M 2738d**	1.5	13.2	28.9	1.1
**ZS 260**	1.9	3.0	3.6	3.0
**N 871a**	2.6	6.9	16.9	17.9
**N 901**	2.4	7.9	21.2	24.7

Table S2.28. Results of interaction with nano tFAST-E46Q

Cmpd	Enhancement			
	430	480	530	580
**HBR-DOM2**	13.2	>155.2	>420.8	20.3
**HBR-2,5-DM**	15.4	>170.4	228.3	1.0
**HMBR**	18.3	>116.2	35.1	1.6
**HBR-DOM**	7.1	77.7	389.6	122.4
**N 871b**	1.5	5.0	20.0	51.8
**MID 145**	1.3	2.9	10.0	2.5
**N 1052**	1.3	2.0	5.2	7.7
**N 1184**	1.7	6.4	25.0	35.0
**N 1122**	1.2	1.7	1.6	1.0
**N 1049**	1.2	1.9	5.7	6.3
**N 1123**	1.1	1.2	2.1	3.8
**N 1139**	1.1	2.0	8.4	16.4
**N 1118**	1.4	2.1	2.6	1.1
**SAI 112**	13.7	>78.5	5.6	1.2
**N 1135**	1.1	1.1	1.3	1.8
**SAI 118**	13.6	49.4	2.9	1.1
**N 1124**	1.1	1.5	3.0	2.7
**SAI 122**	10.2	33.8	1.9	1.2
**N 1142**	1.1	1.4	2.7	1.8
**SAI 199**	17.4	59.3	1.9	1.3
**N 967**	1.1	1.4	3.2	7.0
**N 1039**	1.9	6.1	26.5	37.9
**N 1202**	1.7	6.1	23.8	48.8
**N 960b**	1.2	1.8	5.0	14.7
**N 1036**	1.2	2.2	1.5	1.3
**SAI 117**	17.0	65.4	3.5	0.9
**N 1042**	1.1	1.0	1.1	1.0
**SAI 121**	11.7	46.4	2.5	1.1
**MID 147**	2.4	4.1	10.2	20.0
**SAI 125**	17.6	71.1	3.6	1.0
**MID 151**	1.1	1.2	1.8	2.8
**N 865**	1.1	1.1	1.1	1.1
**MID 153**	1.1	1.2	1.6	2.2
**MID 343**	1.2	1.3	3.6	12.6
**M 3007a**	1.1	1.3	3.2	5.0
**MID 367**	1.1	1.1	1.7	4.3
**N 1048**	1.4	2.2	10.8	30.2
**N 979**	1.1	1.1	1.1	1.1
**N 971**	1.1	1.1	1.2	1.3
**N 973**	1.1	1.1	1.2	1.3
**N 976**	1.1	1.1	1.3	1.6
**N 980**	1.1	1.0	1.1	1.0
**N 1205**	3.4	19.5	76.4	76.1
**N 960a**	1.1	1.2	1.4	1.5

**ZS 331**	1.4	2.3	4.8	9.5
**N 1027**	1.1	1.0	1.0	1.0
**M 2766**	1.7	5.8	22.5	36.7
**ZS 362**	1.1	1.1	1.0	1.0
**N 1179**	1.2	1.2	1.2	0.9
**N 1180**	1.2	1.2	1.1	1.0
**N 1204**	2.8	14.6	56.2	35.3
**N 1206**	1.1	1.4	4.1	8.1
**ZS 309**	1.8	3.0	5.0	4.3
**ZS 316**	1.1	1.1	1.2	1.0
**ZS 319**	1.1	1.1	1.1	1.0
**ZS 325**	1.1	1.1	1.2	1.1
**MID 213**	1.2	1.3	1.9	2.4
**M 2767**	1.5	2.9	6.7	10.6
**M 2876**	1.1	1.2	1.1	1.0
**SH 16**	1.7	5.6	12.9	1.1
**SAI 472**	4.3	14.6	6.2	1.1
**SAI 499**	1.6	2.2	1.2	1.1
**SAI 503**	1.3	1.1	1.2	1.0
**SAI 474**	5.1	1.1	1.1	1.0
**SAI 477**	45.1	31.6	1.1	1.0
**N 1196**	1.1	1.2	1.2	1.1
**N 1197**	1.1	1.1	1.1	1.0
**SAI 127**	14.3	>77.9	3.8	1.0
**N 1056**	1.1	1.1	1.5	1.9
**N 1068**	1.1	1.1	1.2	1.1
**N 1069**	1.1	1.3	1.4	1.1
**A9**	1.1	1.2	1.2	1.1
**N 1198**	1.1	1.1	1.1	1.0
**N 1193**	3.4	5.6	5.2	5.8
**N 1199**	1.3	2.0	4.7	2.7
**SAI 363**	9.5	51.4	2.9	1.0
**SAI 366**	18.6	>99.8	28.7	1.0
**SAI 362**	19.0	>134.4	21.4	1.0
**SAI 365**	16.9	>95.7	32.4	1.0
**SAI 120**	1.2	1.3	1.3	1.1
**SAI 458**	14.3	85.9	20.2	1.0
**SAI 459**	1.1	1.1	1.1	1.1
**SAI 487**	1.1	1.1	1.1	1.1
**N 1131**	1.3	2.8	2.2	1.0
**MID 323**	1.2	1.3	3.2	4.6
**SAI 379**	7.4	12.5	1.4	1.1
**SAI 367**	2.7	3.1	2.5	1.0
**M 2738d**	1.3	6.1	12.0	1.2
**ZS 260**	2.7	6.1	8.1	3.6
**N 871a**	3.3	7.9	20.3	23.1
**N 901**	4.2	19.2	60.0	57.8

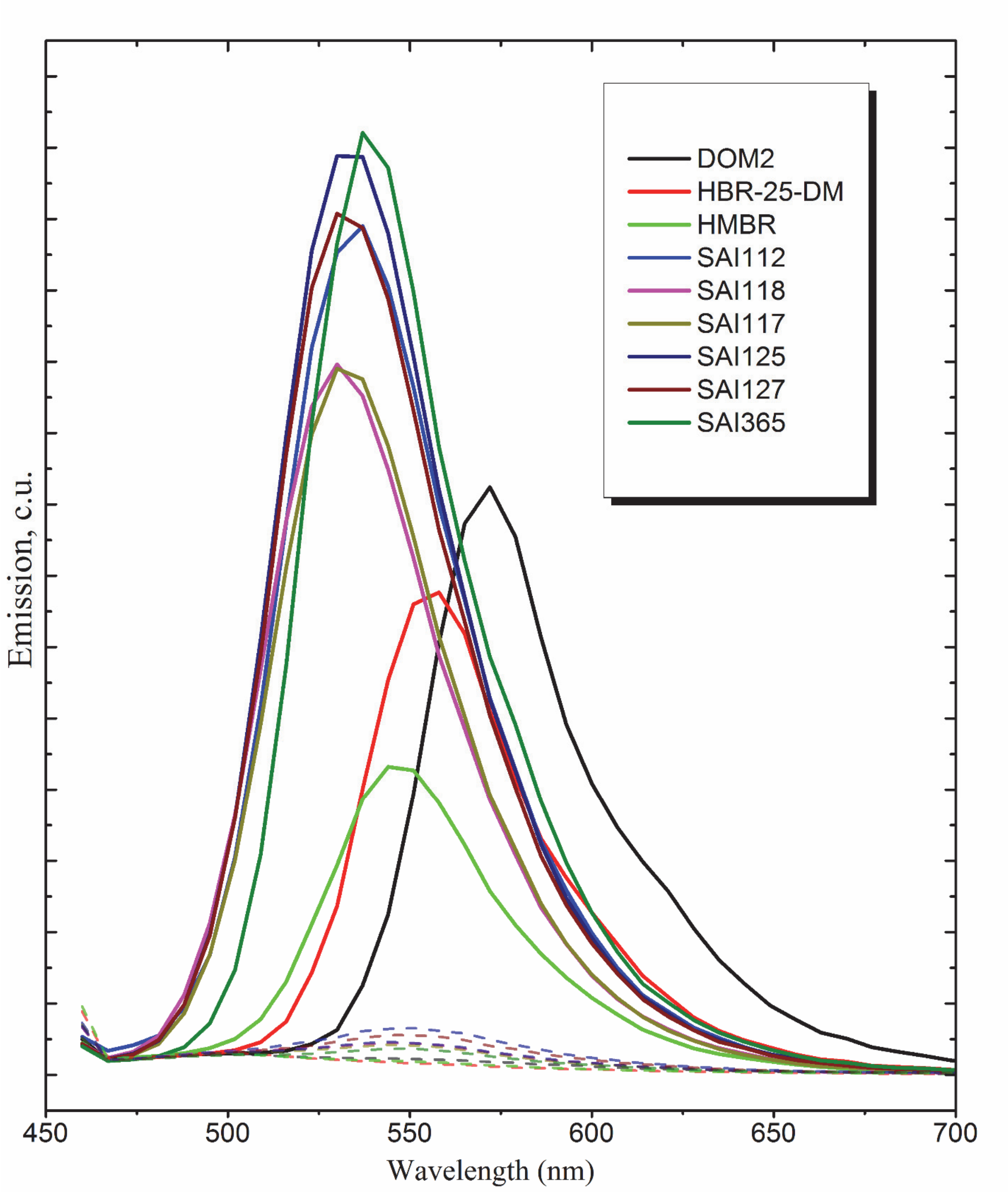

Figure S2.1. Emission spectra of various compounds in bound (solid lines) with nano-redFAST protein and free (dash lines) forms obtained from plate reader screening. The spectra are presented as is, without normalization (see the relevant part of the Methods section). Solutions with a concentration of 10 μM of corresponding fluorogens and 1 μM of nanoFAST variants in the PBS buffer were used.

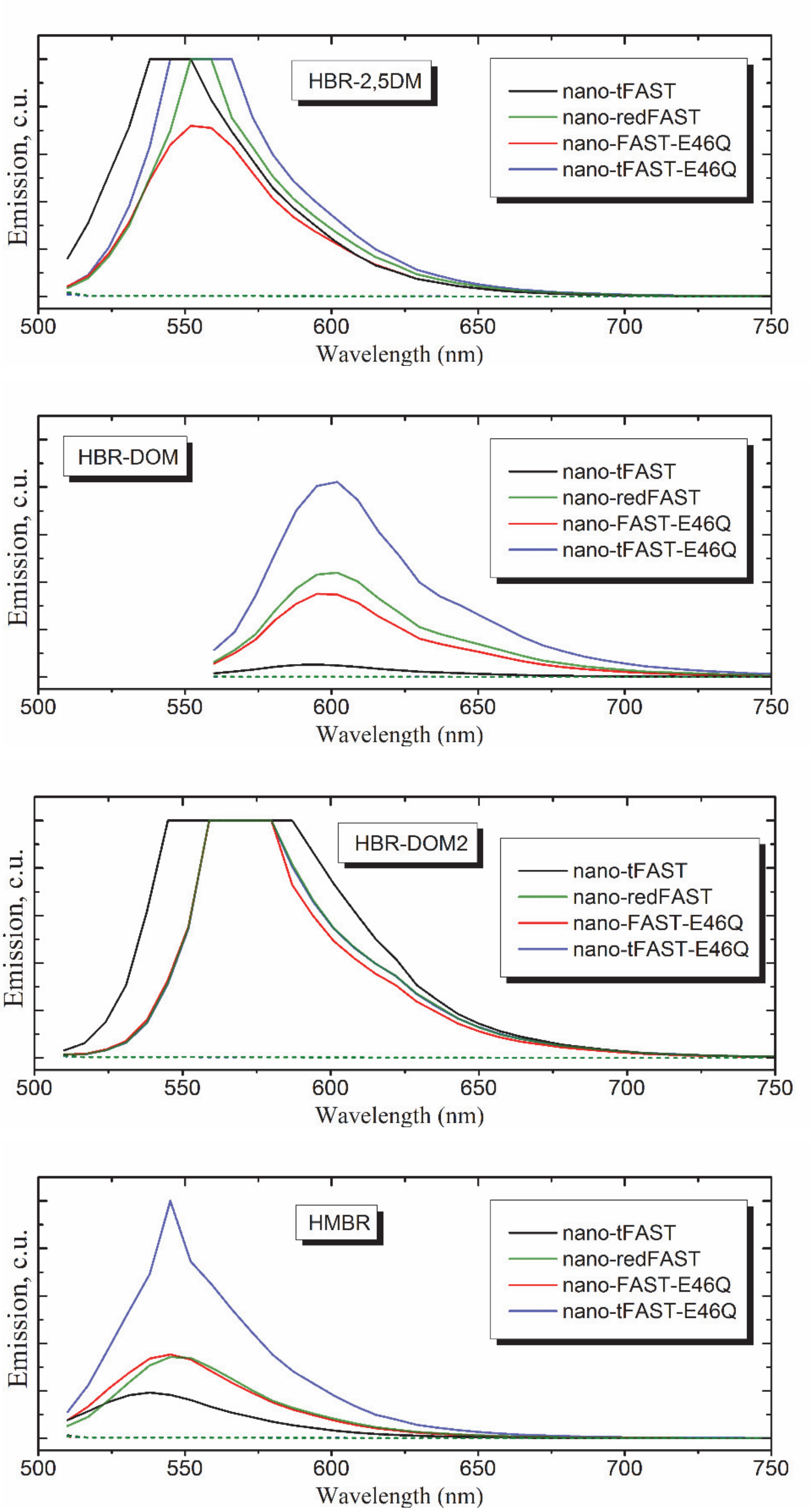

Figure S2.2. Emission spectra of various compounds in bound (solid lines) with nano-FAST variants and free (dash lines) forms obtained from plate reader screening. The spectra are presented as is, without normalization (see the relevant part of the Methods section). Solutions with a concentration of 10 µM of corresponding fluorogens and 1 µM of nanoFAST variants in the PBS buffer were used.

3. Determination of affinity constants

Table S3.1. Dissociation constants values of complexes [mutant-**HBR-DOM2**]

Mutant	$K_{D.}$ µM
**nano-iFAST**	1.19±0.06
**nano-frFAST**	1.12±0.22
**nano-pFAST**	0.074±0.016
**nano-tFAST**	0.19±0.02
**nano-redFAST**	0.70±0.13
**nanoFAST-E46Q**	0.43±0.05
**nano tFAST-E46Q**	0.13±0.01
K_D of complexes of others mutants >3	

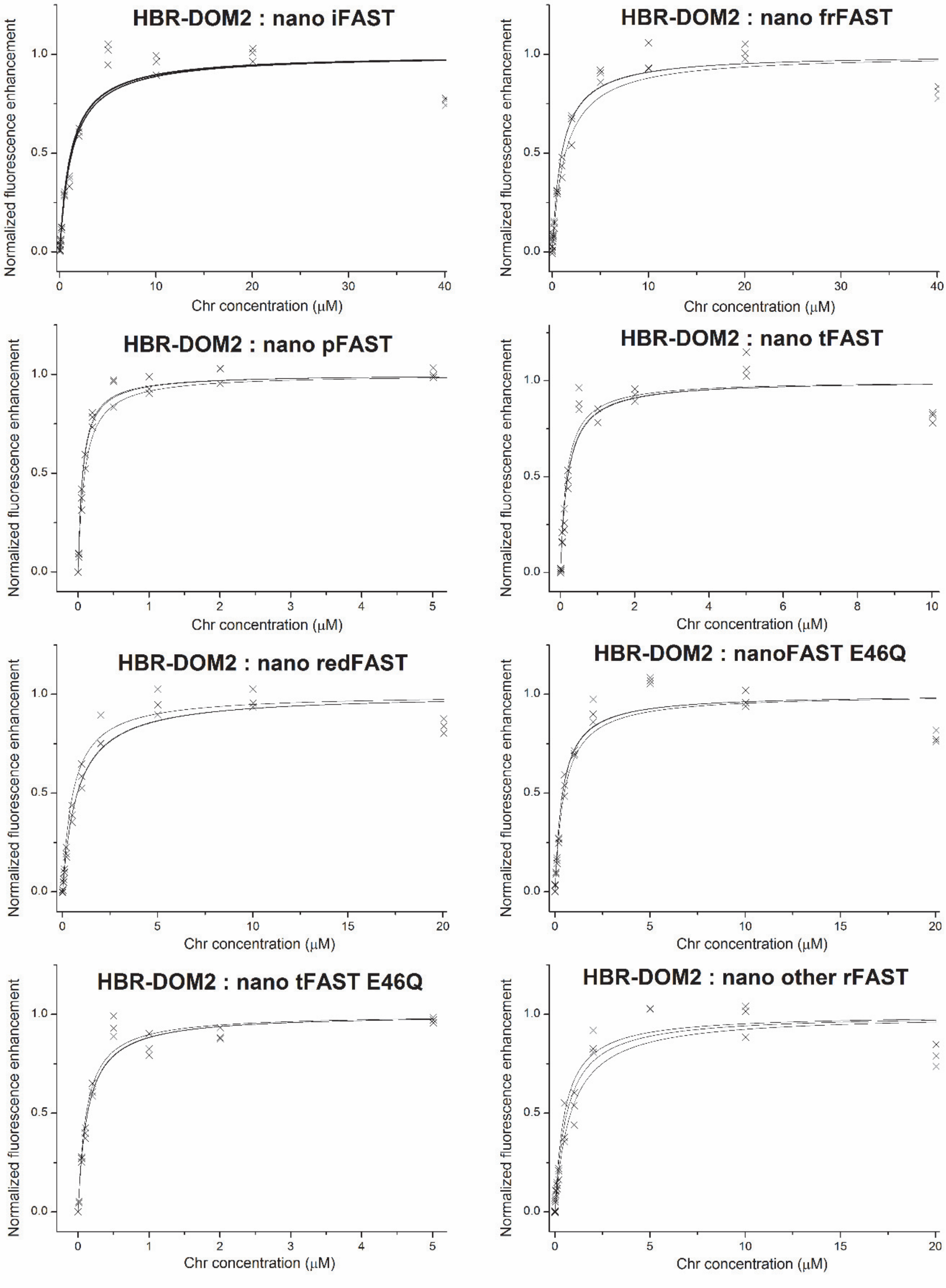

Figure S3.1. The graphs show the bound fraction for various **HBR-DOM2** concentrations.

Table S3.2. Dissociation constants values of complexes [mutant-**HBR-2,5-DM**]

Mutant	$K_{D,}$ μM
**nano-tFAST**	0.93±0.19
**nano-redFAST**	1.26±0.33
**nano-tFAST E46Q**	0.50±0.16

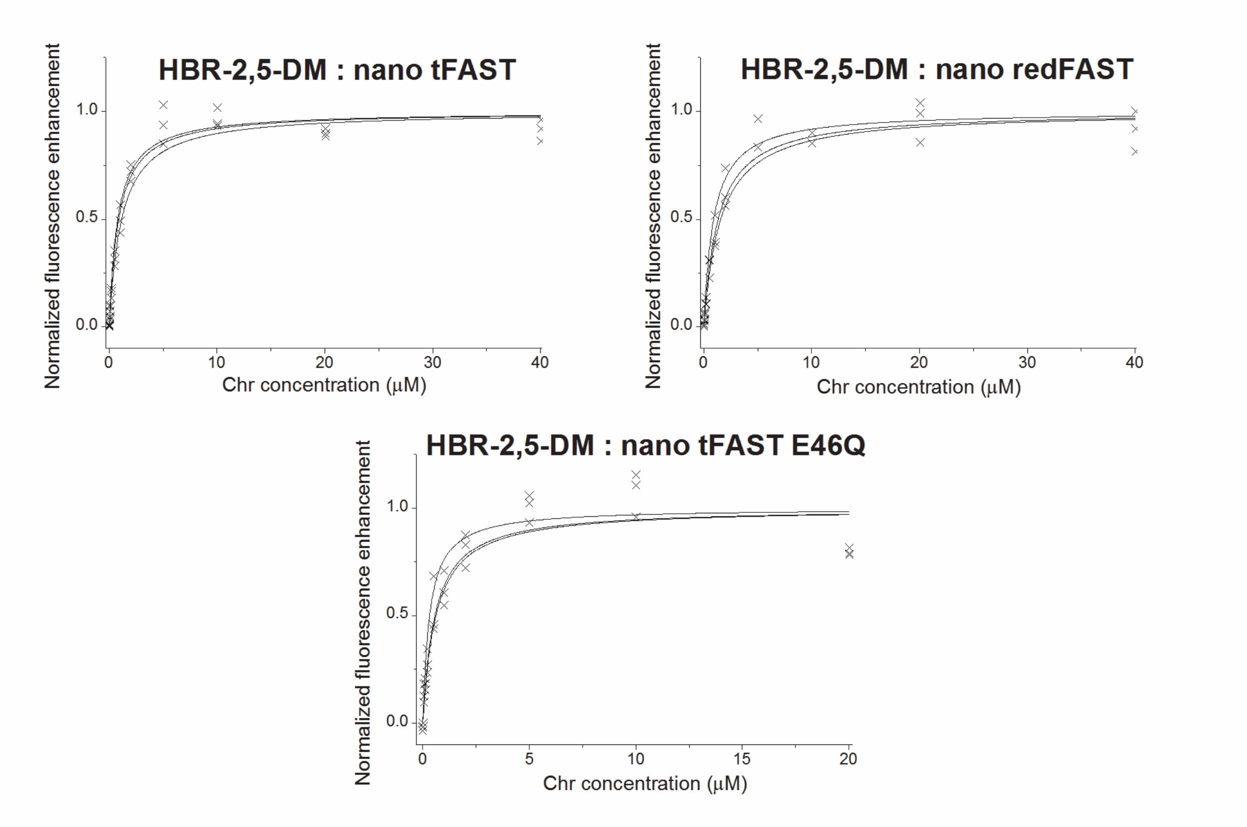

Figure S3.2. The graphs show the bound fraction for various **HBR-2,5-DM** concentrations.

Table S3.3. Dissociation constants values of complexes [mutant-**HMBR**]

Mutant	K_{D}, µM
**nano-redFAST**	>3
**nano-tFAST-E46Q**	>3

Table S3.4. Dissociation constants values of complexes [mutant-**HBR-DOM**]

Mutant	K_{D}, µM
**nano-redFAST**	>3
**nano-tFAST-E46Q**	1.69±0.32

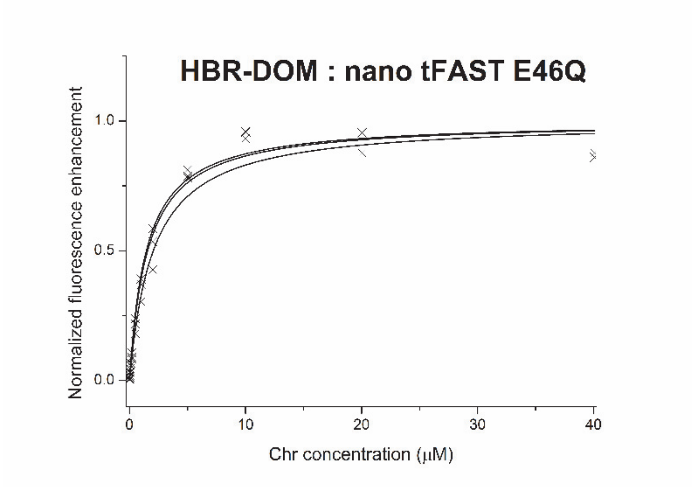

Figure S3.3. The graph shows the bound fraction for various **HBR-DOM** concentrations.

Table S3.5. Dissociation constants values of complexes [**nano-tFAST**-chromophore]

Chromophore	$K_{D.}$ µM
**HBR-DOM2**	0.19±0.02
**HBR-2,5-DM**	0.93±0.19

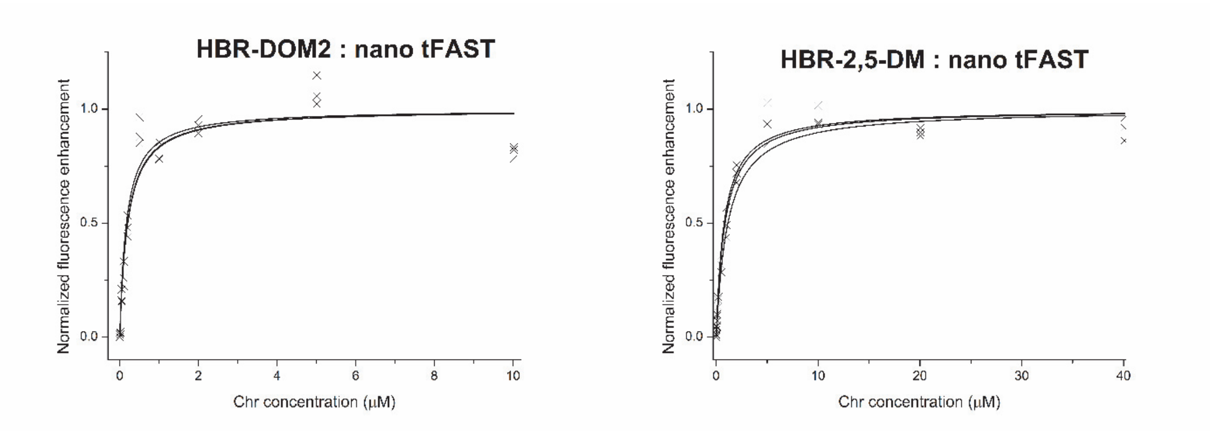

Figure S3.4. The graph shows the bound fraction for various chromophore concentrations.

Table S3.6. Dissociation constants values of complexes [**nano-redFAST**- chromophore]

Chromophore	Enhancement in screening	Structure	$K_{D.}$ µM
**HBR-DOM2**	>400	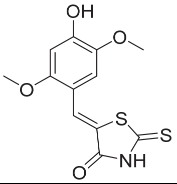	0.69±0.08
**HBR-2,5-DM**	194	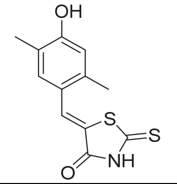	1.23±0.13
**HMBR**	55	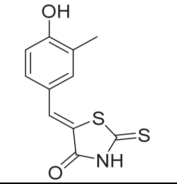	>3
**HBR-DOM**	205	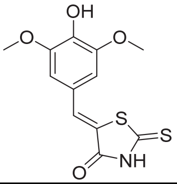	>3
**SAI 112**	>65	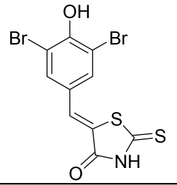	0.055±0.018
**SAI 118**	50	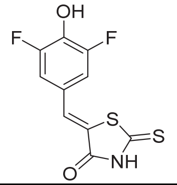	0.27±0.08
**SAI 199**	52	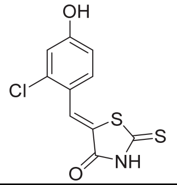	0.16±0.04
**SAI 117**	52	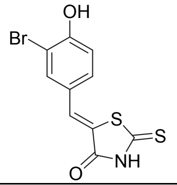	0.27±0.06
**SAI 125**	67	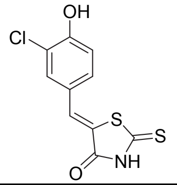	0.59±0.11

**SAI 127**	>70	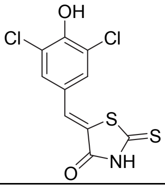	0.041±0.004
**SAI 366**	>89	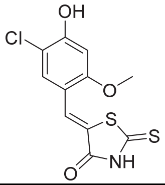	0.022±0.008
**SAI 362**	>105	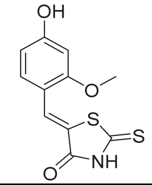	1.01±0.10
**SAI 365**	>87	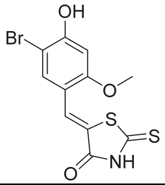	0.026±0.009

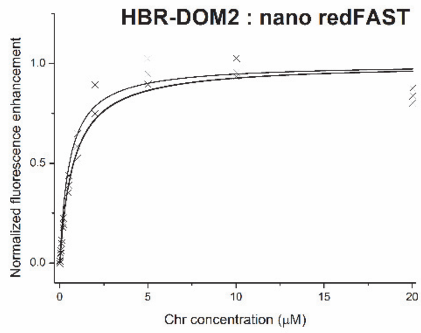
HBR-DOM2 : nano redFAST
Normalized fluorescence enhancement
Chr concentration (μM)

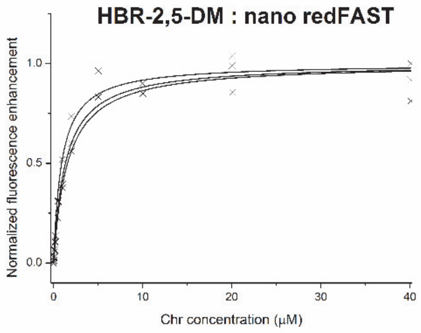
HBR-2,5-DM : nano redFAST
Normalized fluorescence enhancement
Chr concentration (μM)

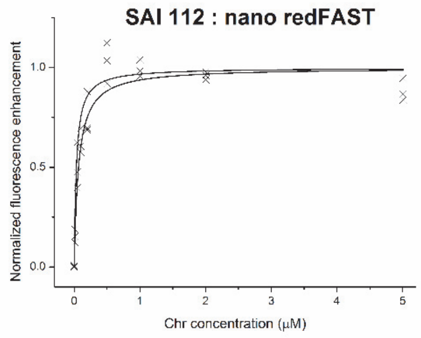
SAI 112 : nano redFAST
Normalized fluorescence enhancement
Chr concentration (μM)

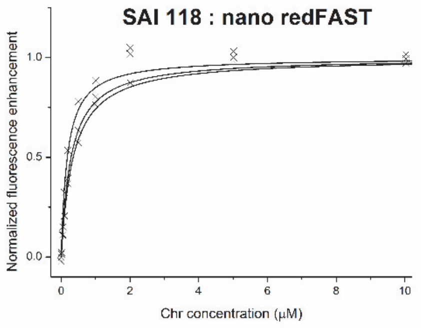
SAI 118 : nano redFAST
Normalized fluorescence enhancement
Chr concentration (μM)

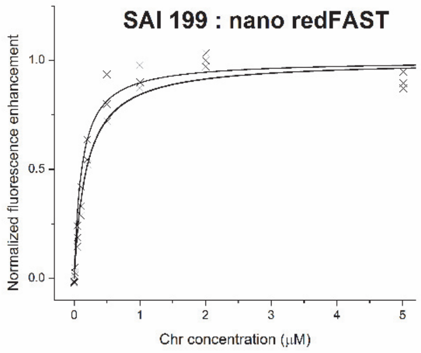
SAI 199 : nano redFAST
Normalized fluorescence enhancement
Chr concentration (μM)

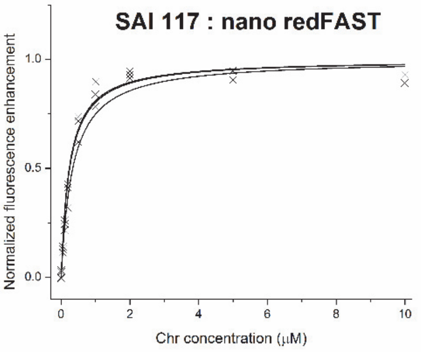
SAI 117 : nano redFAST
Normalized fluorescence enhancement
Chr concentration (μM)

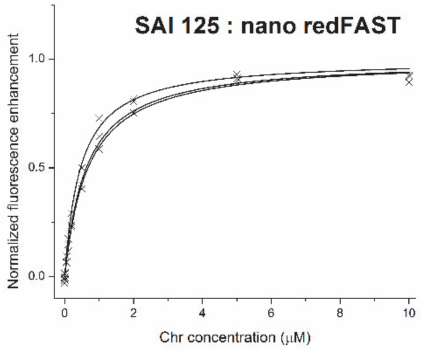
SAI 125 : nano redFAST
Normalized fluorescence enhancement
Chr concentration (μM)

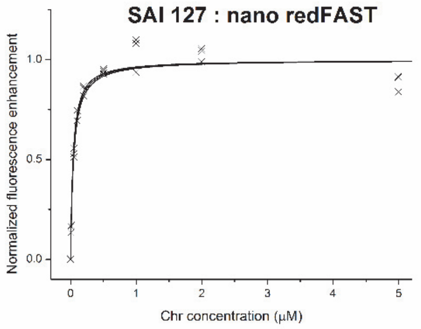
SAI 127 : nano redFAST
Normalized fluorescence enhancement
Chr concentration (μM)

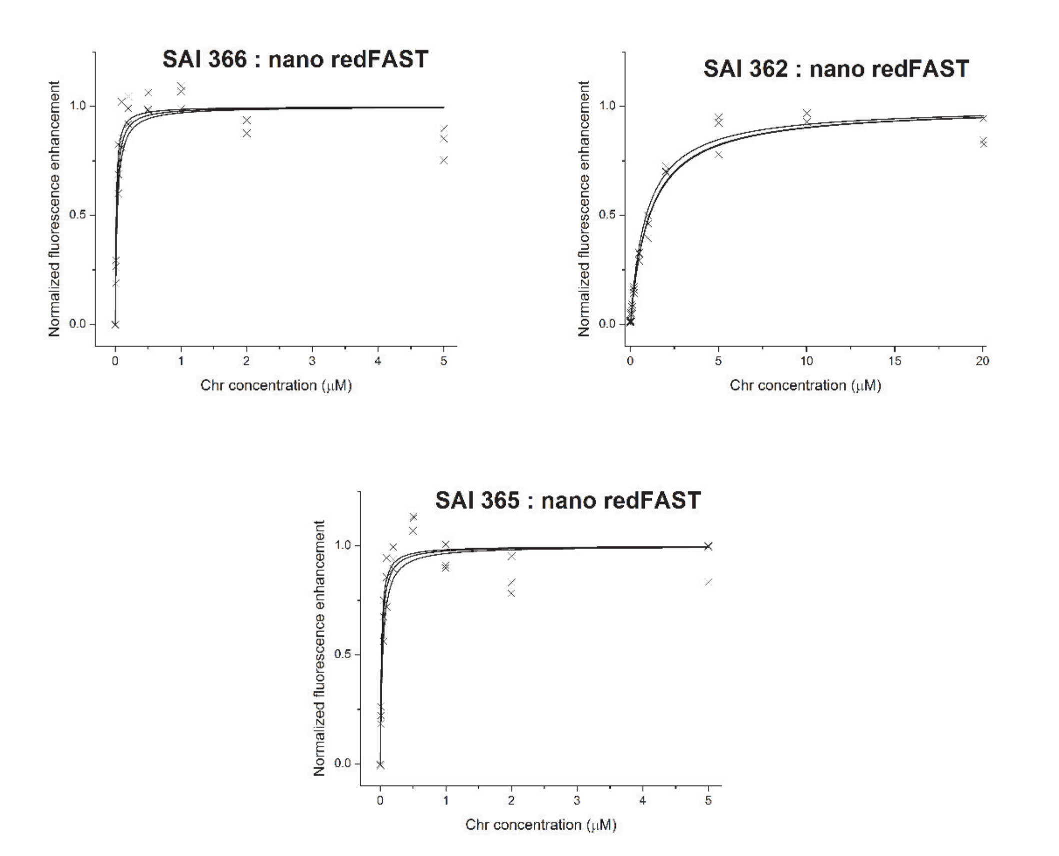

Figure S3.5. The graph shows the bound fraction for various chromophore concentrations.

Table S3.7. Dissociation constants values of complexes [**nano-tFAST-E46Q**-chromophore]

Chromophore	K_{D}. µM
**HBR-DOM2**	0.13±0.01
**HBR-2,5-DM**	0.50±0.16
**HMBR**	>3
**HBR-DOM**	1.69±0.32

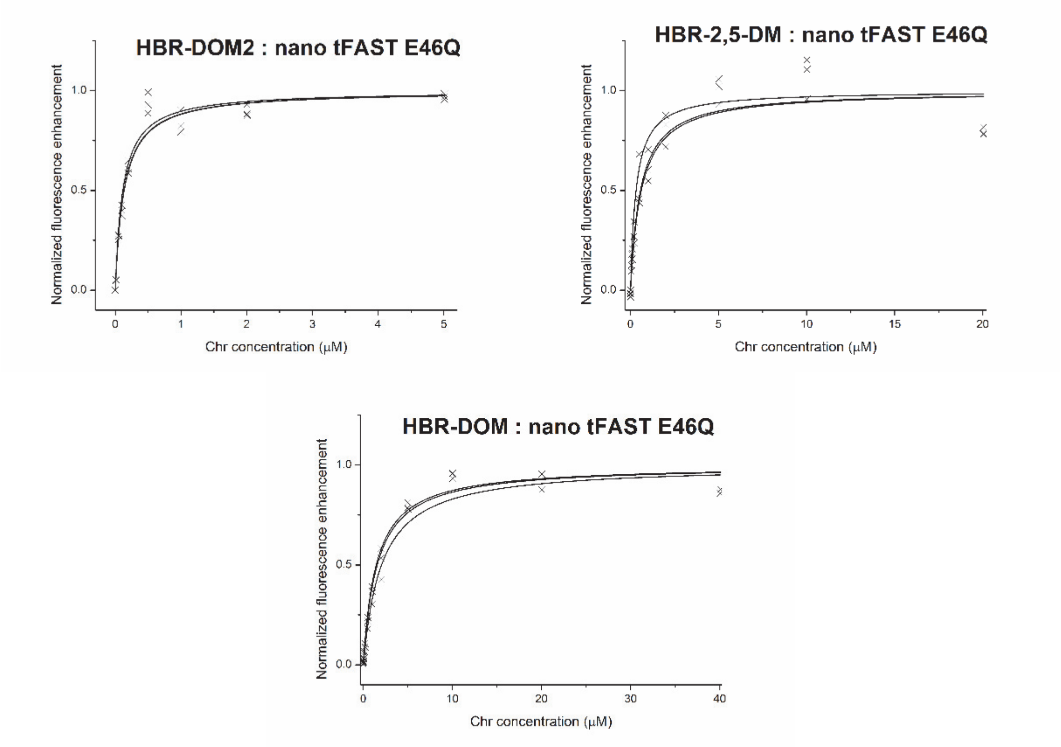

Figure S3.6. The graph shows the bound fraction for various chromophore concentrations.

4. Fluorescence Lifetime Measurements

Table S.4.1. Fluorescence lifetimes (τ) of **HBR-DOM2**, **HBR-DOM**, and **HBR-2,5-DM** fluorogen in complexes with nanoFAST variants. τ is the fluorescence lifetime of the corresponding exponential component; A is a relative contribution of the exponential decay component; χ^2 is a value of the Pearson's chi-squared test.

Fluoorogen	FAST variant	$\tau_1 \pm$ SD, ns	A_1, %	$\tau_2 \pm$ SD, ns	A_2, %	χ^2
**HBR-DOM2**	**nanoFAST**	0.095 ± 0.008	4	3.802 ± 0.007	96	1.166
	**nano-iFAST**	0.106 ± 0.008	4	3.644 ± 0.007	96	1.182
	**nano-redFAST**	0.091 ± 0.008	3	3.993 ± 0.007	97	1.196
	**nano tFAST**	0.090 ± 0.009	4	3.920 ± 0.007	96	1.226
	**nano tFAST-E46Q**	0.100 ± 0.008	3	4.038 ± 0.007	97	1.196
**HBR-DOM**	**nano tFAST-E46Q**	0.121 ± 0.008	5	3.377 ± 0.007	95	1.172
**HBR-2,5-DM**	**nano tFAST-E46Q**	0.102 ± 0.007	7	2.399 ± 0.006	93	1.200

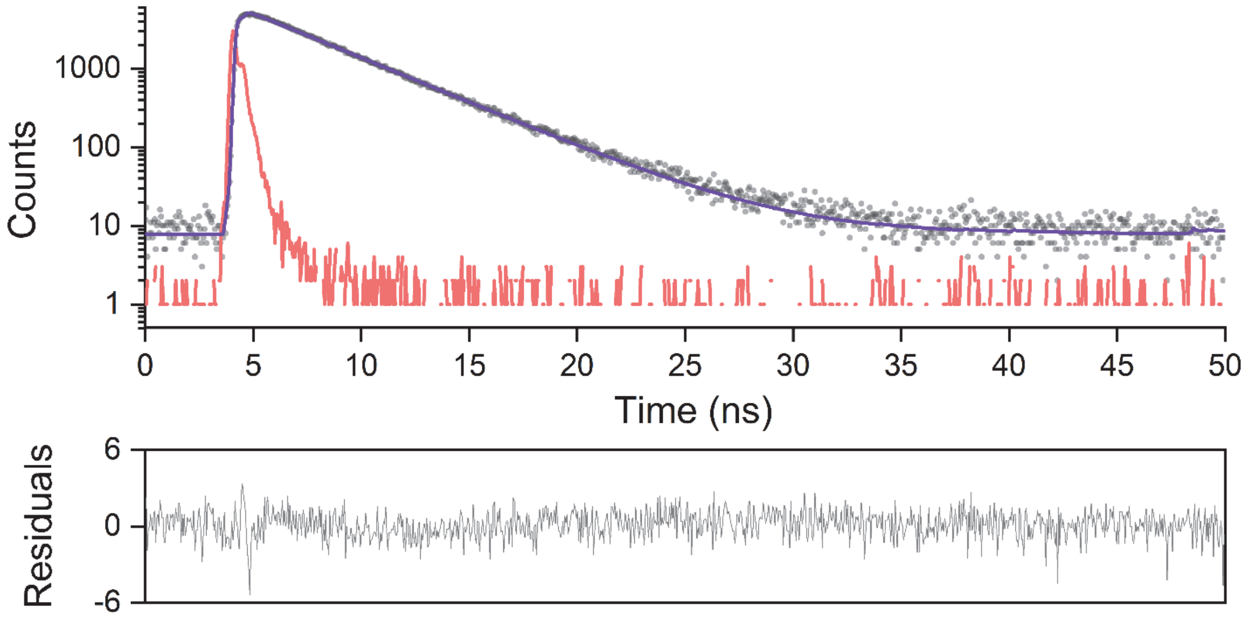

Figure S4.1. Fluorescence decay kinetics of the **HBR-DOM2** fluorogen bound by nanoFAST. Gray dots represent experimental decay data (photon arrivals), violet line shows exponential fit of the data, red curve denotes instrument response function (IRF). Residuals of fitting results are shown below.

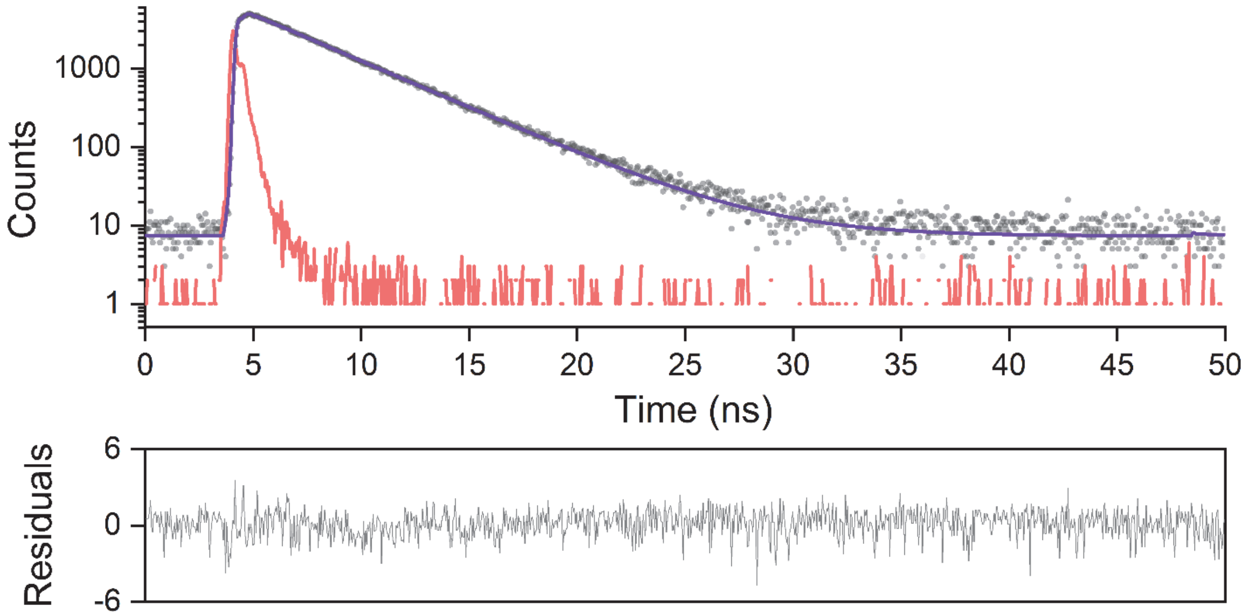

Figure S4.2. Fluorescence decay kinetics of the **HBR-DOM2** fluorogen bound by nano-iFAST. Gray dots represent experimental decay data (photon arrivals), violet line shows exponential fit of the data, red curve denotes instrument response function (IRF). Residuals of fitting results are shown below.

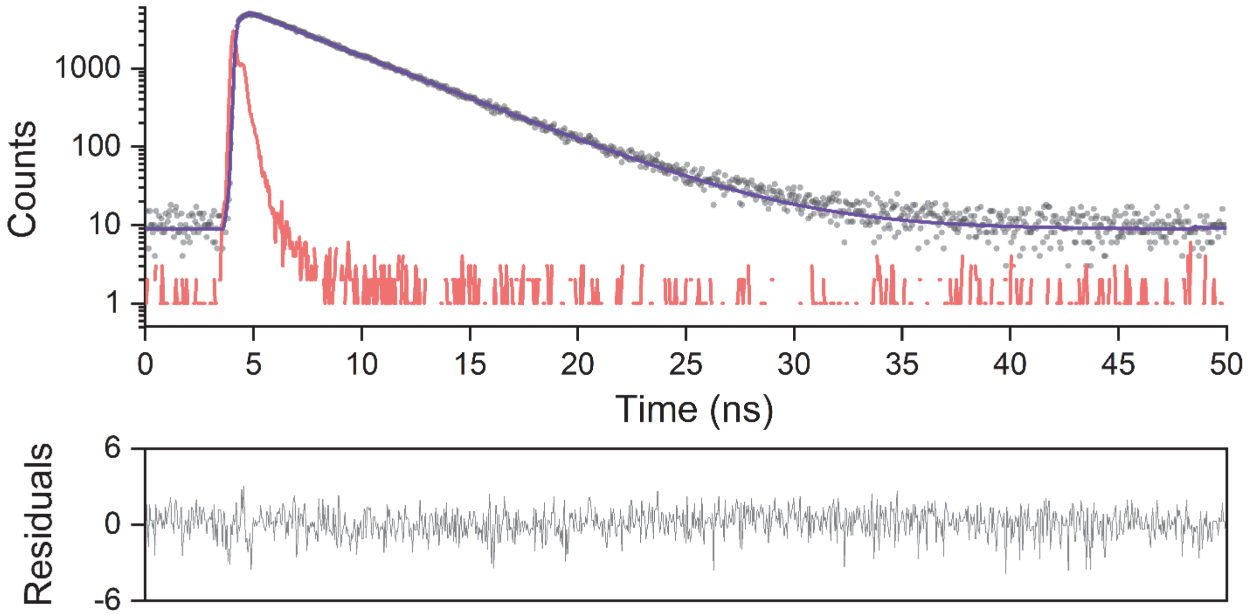

Figure S4.3. Fluorescence decay kinetics of the **HBR-DOM2** fluorogen bound by nano-redFAST. Gray dots represent experimental decay data (photon arrivals), violet line shows exponential fit of the data, red curve denotes instrument response function (IRF). Residuals of fitting results are shown below.

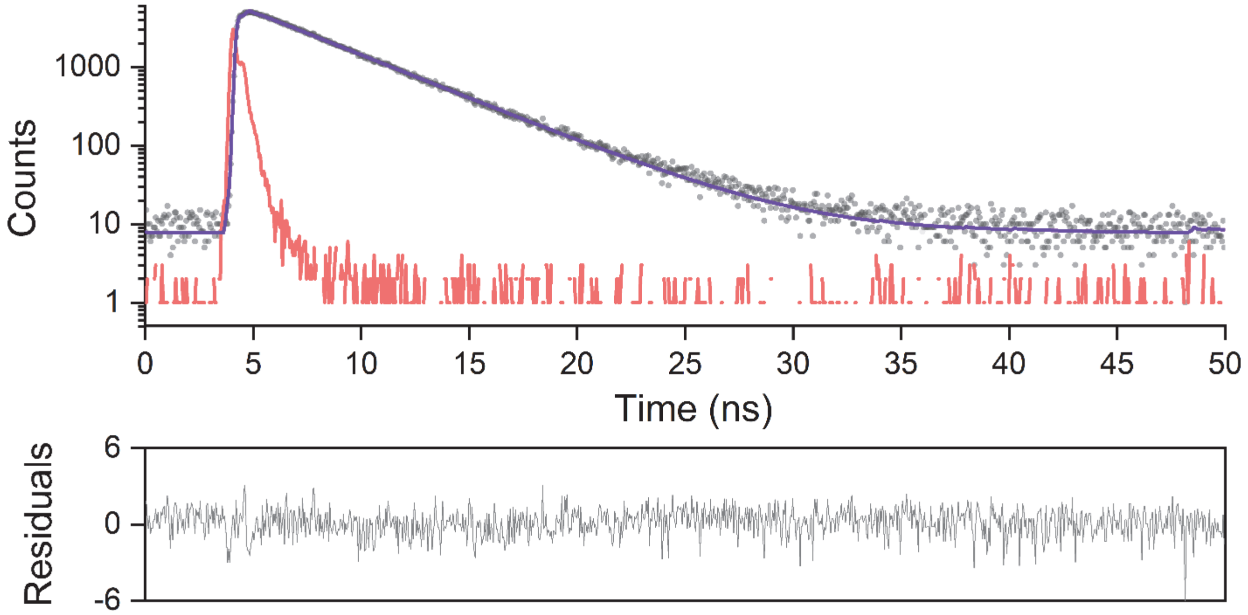

Figure S4.4. Fluorescence decay kinetics of the **HBR-DOM2** fluorogen bound by nano-tFAST. Gray dots represent experimental decay data (photon arrivals), violet line shows exponential fit of the data, red curve denotes instrument response function (IRF). Residuals of fitting results are shown below.

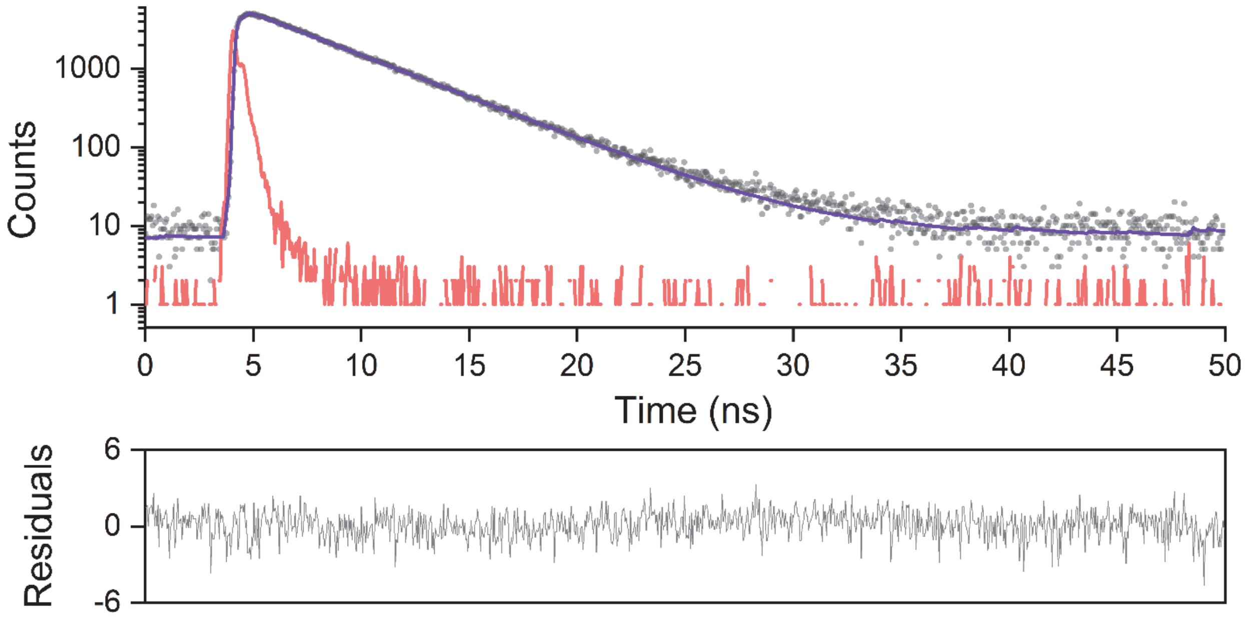

Figure S4.5. Fluorescence decay kinetics of the **HBR-DOM2** fluorogen bound by nano-tFAST-E46Q. Gray dots represent experimental decay data (photon arrivals), violet line shows exponential fit of the data, red curve denotes instrument response function (IRF). Residuals of fitting results are shown below.

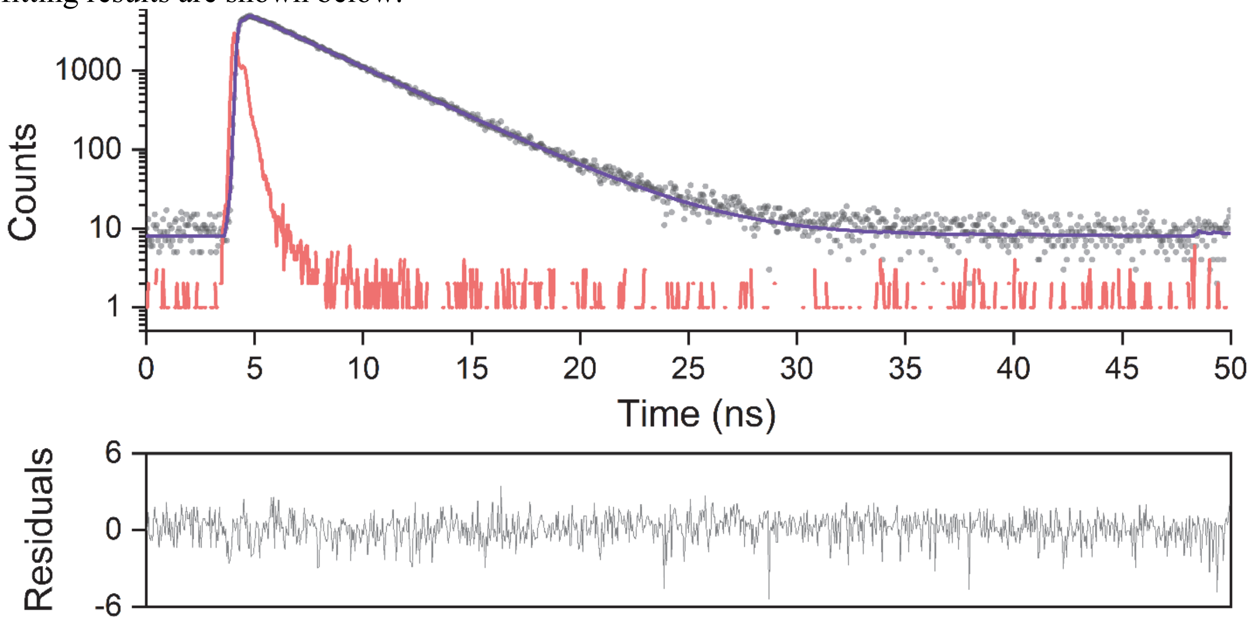

Figure S4.6. Fluorescence decay kinetics of the **HBR-DOM** fluorogen bound by nano-tFAST-E46Q. Gray dots represent experimental decay data (photon arrivals), violet line shows exponential fit of the data, red curve denotes instrument response function (IRF). Residuals of fitting results are shown below.

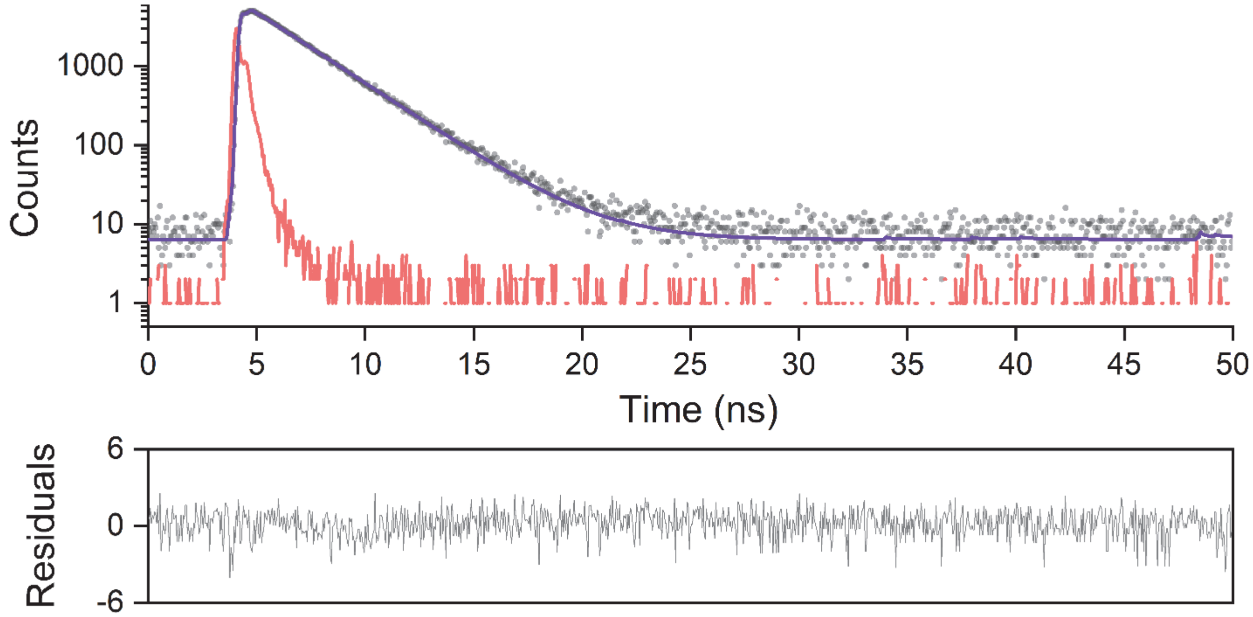

Figure S4.7. Fluorescence decay kinetics of the **HBR-2,5DM** fluorogen bound by nano-tFAST-E46Q. Gray dots represent experimental decay data (photon arrivals), violet line shows exponential fit of the data, red curve denotes instrument response function (IRF). Residuals of fitting results are shown below.

5. Spectra of complexes

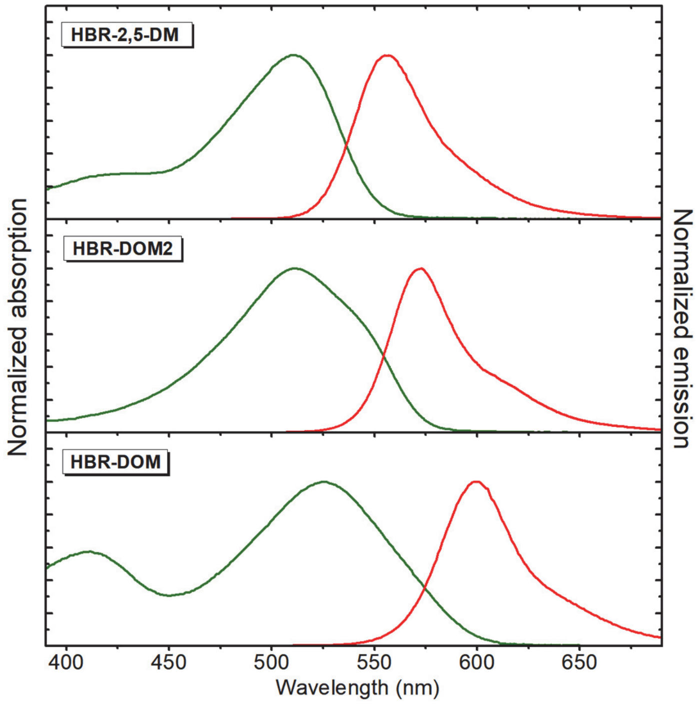

Figure S5.1. The absorption (green) and emission (red) spectra of various chromophores in complexes with nano-tFAST-E46Q.

6. Fluorescent microscopy

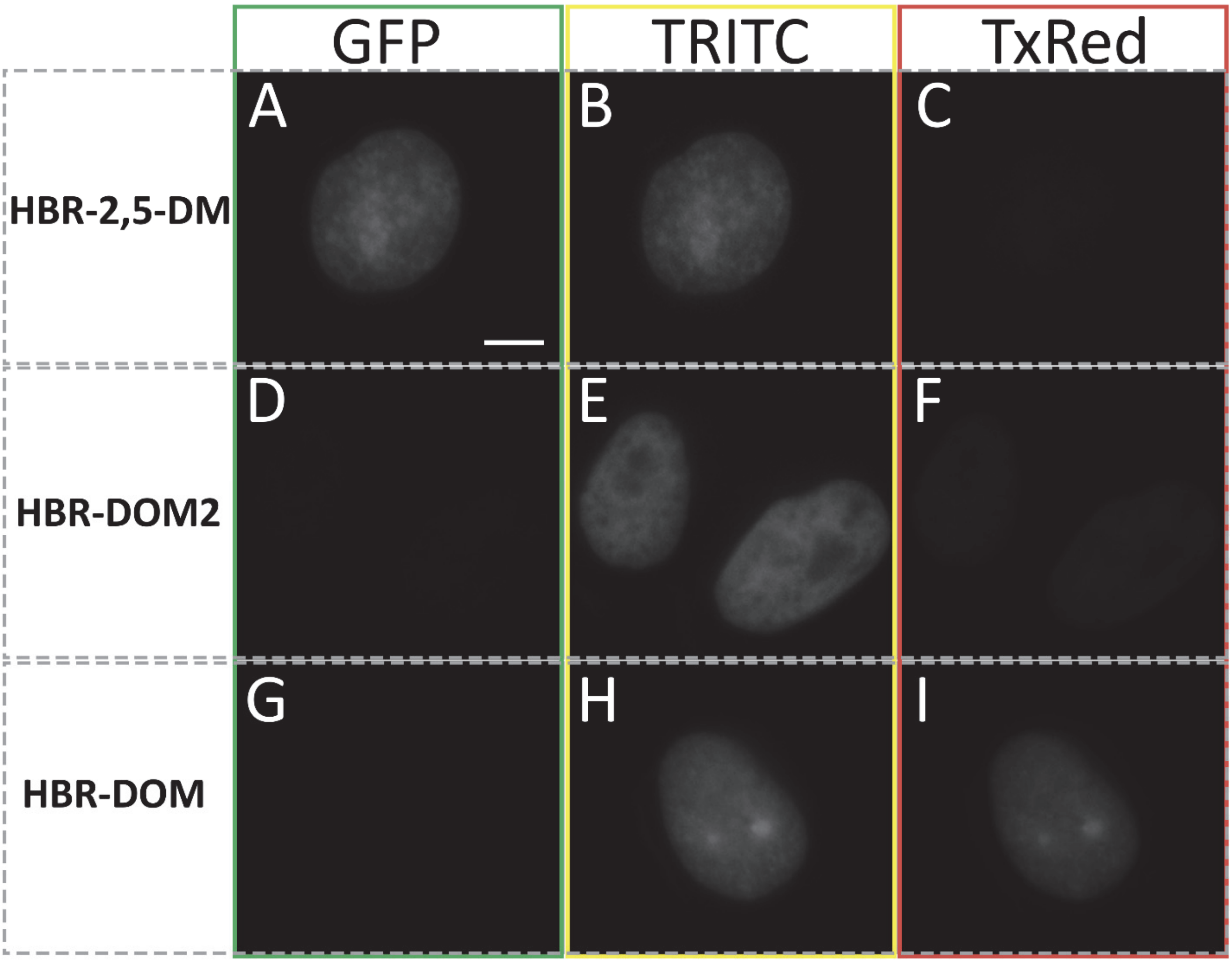

Figure S6.1. Live-cell imaging with a set of fluorogens using different filter sets. HeLa Kyoto cells transiently transfected with nano-tFast-E46Q-H2B. Cells were imaged using GFP filter (A, D, G), TRITC filter (B, E, H), and TxRed filter (C, F, I) in the presence of 5 μM **HBR-2,5-DM** (A, B, C), 5 μM **HBR-DOM2** (D, E, F), and 5 μM **HBR-DOM** (G, H, I). The scale bar is 10 μm. Imaging conditions, e.g. light intensity, gain, and exposure time for every filter set, were kept constant.

7. Compounds description

(*Z*)-5-(4-hydroxybenzylidene)-2-thioxothiazolidin-4-ones

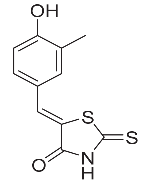

(Z)-5-(4-Hydroxy-3-methylbenzylidene)-2-thioxothiazolidin-4-one (HMBR)

Previously characterized. Spectral properties correspond to the previously reported.[3]

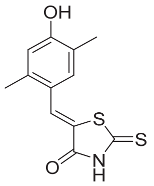

(Z)-5-(4-Hydroxy-2,5-dimethylbenzylidene)-2-thioxothiazolidin-4-one (HBR-2,5-DM)

Previously characterized. Spectral properties correspond to the previously reported.[4]

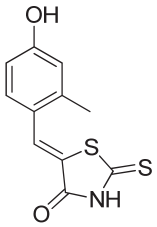

(Z)-5-(4-Hydroxy-2-methylbenzylidene)-2-thioxothiazolidin-4-one (SAI458)

Yield 204 mg (27%). Orange solid. M.p. over 250°C.

E/Z isomerism is observed for this compound. A description for the major isomer is given below.

^{1}H NMR (700 MHz, DMSO-d_6) δ 13.69 (brs, 1H), 10.24 (s, 1H), 7.67 (s, 1H), 7.25 (d, J = 8.5 Hz, 1H), 6.79 (dd, J = 8.5, 2.6 Hz, 1H), 6.75 (d, J = 2.6 Hz, 1H), 2.37 (s, 3H).

^{13}C NMR (176 MHz, DMSO-d_6) δ 195.8, 169.3, 160.1, 142.1, 130.1, 129.4, 122.7, 122.0, 118.0, 114.2, 19.5,

HRMS (ESI) m/z [M+H]$^+$ calcd for $C_{11}H_{10}NO_2S_2$: 252.0147, found: 252.0147.

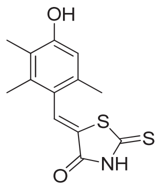

(Z)-5-(4-Hydroxy-2,3,6-trimethylbenzylidene)-2-thioxothiazolidin-4-one (SAI459)

Yield 142 mg (17%). Orange solid. M.p. 246-248°C.

E/Z isomerism is observed for this compound. A description for the major isomer is given below.

^{1}H NMR (700 MHz, DMSO-d_6) δ 13.64 (brs, 1H), 9.47 (s, 1H), 7.68 (s, 1H), 6.58 (s, 1H), 2.10 (s, 3H), 2.07 (s, 3H), 2.03 (s, 3H).

^{13}C NMR (176 MHz, DMSO-d_6) δ 196.4, 167.8, 155.8, 135.1, 134.0, 133.3, 130.9, 124.2, 120.7, 114.2, 19.8, 17.1, 11.5,

HRMS (ESI) m/z $[M+H]^+$ calcd for $C_{13}H_{14}NO_2S_2$: 280.0460, found: 280.0454.

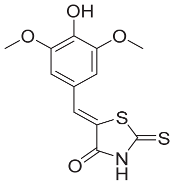

(Z)-5-(4-Hydroxy-3,5-dimethoxybenzylidene)-2-thioxothiazolidin-4-one (HBR-DOM)

Previously characterized. Spectral properties correspond to the previously reported.[4]

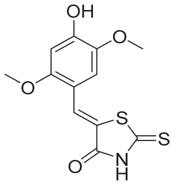

(Z)-5-(4-Hydroxy-2,5-dimethoxybenzylidene)-2-thioxothiazolidin-4-one (HBR-DOM2)

Previously characterized. Spectral properties correspond to the previously reported.[2]

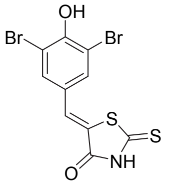

(*Z*)-5-(3,5-Dibromo-4-hydroxybenzylidene)-2-thioxothiazolidin-4-one (SAI112)

Previously characterized. Spectral properties correspond to the previously reported.[2]

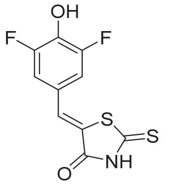

(*Z*)-5-(3,5-Difluoro-4-hydroxybenzylidene)-2-thioxothiazolidin-4-one (SAI118)

Previously characterized. Spectral properties correspond to the previously reported.[2]

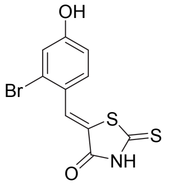

(*Z*)-5-(2-Bromo-4-hydroxybenzylidene)-2-thioxothiazolidin-4-one (SAI122)

Previously characterized. Spectral properties correspond to the previously reported.[2]

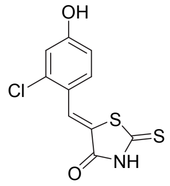

(*Z*)-5-(2-Chloro-4-hydroxybenzylidene)-2-thioxothiazolidin-4-one (SAI199)

Previously characterized. Spectral properties correspond to the previously reported.[2]

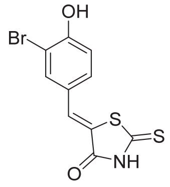

(*Z*)-5-(3-Bromo-4-hydroxybenzylidene)-2-thioxothiazolidin-4-one (SAI117)

Previously characterized. Spectral properties correspond to the previously reported.[2]

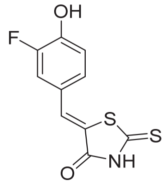

(*Z*)-5-(3-Fluoro-4-hydroxybenzylidene)-2-thioxothiazolidin-4-one (SAI121)

Previously characterized. Spectral properties correspond to the previously reported.[2]

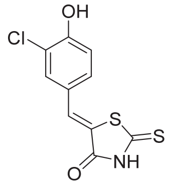

(*Z*)-5-(3-Chloro-4-hydroxybenzylidene)-2-thioxothiazolidin-4-one (SAI125)

Previously characterized. Spectral properties correspond to the previously reported.[2]

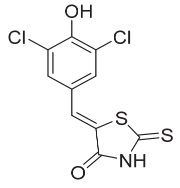

(*Z*)-5-(3,5-Dichloro-4-hydroxybenzylidene)-2-thioxothiazolidin-4-one (SAI127)

Previously characterized. Spectral properties correspond to the previously reported.[2]

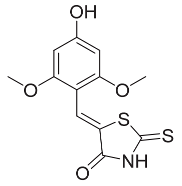

(*Z*)-5-(4-Hydroxy-2,6-dimethoxybenzylidene)-2-thioxothiazolidin-4-one (SAI363)

Previously characterized. Spectral properties correspond to the previously reported.[2]

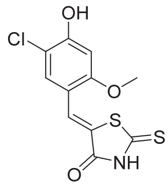

(*Z*)-5-(5-Chloro-4-hydroxy-2-methoxybenzylidene)-2-thioxothiazolidin-4-one (SAI366)

Previously characterized. Spectral properties correspond to the previously reported.[2]

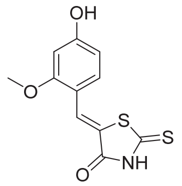

(*Z*)-5-(4-Hydroxy-2-methoxybenzylidene)-2-thioxothiazolidin-4-one (SAI362)

Previously characterized. Spectral properties correspond to the previously reported.[2]

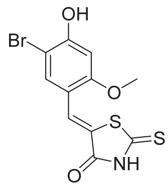

(*Z*)-5-(5-Bromo-4-hydroxy-2-methoxybenzylidene)-2-thioxothiazolidin-4-one (SAI365)

Previously characterized. Spectral properties correspond to the previously reported.[2]

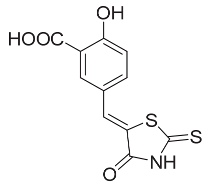

(*Z*)-2-Hydroxy-5-((4-oxo-2-thioxothiazolidin-5-ylidene)methyl)benzoic acid (SAI120)

Previously characterized. Spectral properties correspond to the previously reported.[2]

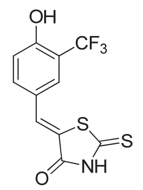

(*Z*)-5-(4-Hydroxy-3-(trifluoromethyl)benzylidene)-2-thioxothiazolidin-4-one (SAI379)

Yield 198 mg (22%). Yellow solid. M.p. 254-256°C.

^{1}H NMR (700 MHz, DMSO-d_6) δ 7.18 (d, J = 8.7 Hz, 1H), 7.63 (s, 1H), 7.66 (dd, J = 8.7, 2.3 Hz, 1H), 7.79 (d, J = 2.4 Hz, 1H), 11.50 (brs, 1H), 13.76 (brs, 1H).

^{13}C NMR (176 MHz, DMSO-d_6) δ 116.5 (q, J = 30.5 Hz), 118.2, 123.1, 123.4 (q, J = 272.4 Hz), 123.8, 130.2 (q, J = 4.8 Hz), 130.9, 135.3, 157.9, 169.3, 195.2.

HRMS (ESI) m/z $[M+H]^+$ calcd for $C_{11}H_7F_3NO_2S_2$: 305.9865, found: 305.9866.

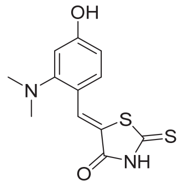

(*Z*)-5-(2-(Dimethylamino)-4-hydroxybenzylidene)-2-thioxothiazolidin-4-one (SAI 367)

Previously characterized. Spectral properties correspond to the previously reported.[2]

5-(4-hydroxybenzylidene)-2-thioxoimidazolidin-4-ones

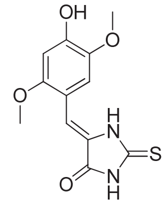

(Z)-5-(4-Hydroxy-2,5-dimethoxybenzylidene)-2-thioxoimidazolidin-4-one (SAI472)

The dried precipitate was subject to flash column chromatography (gradient elution with $CHCl_3$ to $CHCl_3$/MeOH, 100:4). Yield 160 mg (19%). Orange solid. M.p. over 250°C.

E/Z isomerism is observed for this compound. A description for the major isomer is given below.

^{1}H NMR (700 MHz, DMSO-d_6) δ 12.19 (s, 1H), 11.98 (s, 1H), 9.77 (s, 1H), 7.19 (s, 1H), 6.71 (s, 1H), 6.54 (s, 1H), 3.84 (s, 3H), 3.77 (s, 3H).

^{13}C NMR (176 MHz, DMSO-d_6) δ 177.8, 165.8, 153.9, 150.5, 141.8, 124.9, 114.4, 111.2, 107.4, 100.2, 57.0, 56.0.

HRMS (ESI) m/z $[M+H]^+$ calcd for $C_{12}H_{13}N_2O_4S$: 281.0591, found: 281.0590.

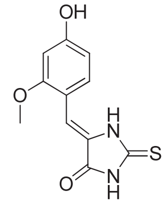

(Z)-5-(4-Hydroxy-2-methoxybenzylidene)-2-thioxoimidazolidin-4-one (SAI499)

Yield 240 mg (32%). Brown solid. M.p. over 250°C.

^{1}H NMR (700 MHz, DMSO-d_6) δ 12.17 (s, 1H), 11.81 (s, 1H), 10.10 (brs, 1H), 7.65 (d, J = 8.5 Hz, 1H), 6.69 (s, 1H), 6.45 (d, J = 2.3 Hz, 1H), 6.42 (dd, J = 8.5, 2.3 Hz, 1H), 3.81 (s, 3H).

^{13}C NMR (75 MHz, DMSO-d_6) δ 177.8, 165.8, 161.0, 159.5, 131.5, 125.0, 112.2, 108.0, 107.2, 99.0, 55.6.

HRMS (ESI) m/z $[M+H]^+$ calcd for $C_{11}H_{11}N_2O_3S$: 251.0485, found: 251.0483.

5-(4-hydroxybenzylidene)-imidazolidine-2,4-diones

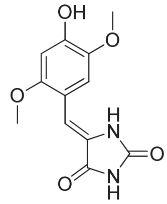

(Z)-5-(4-Hydroxy-2,5-dimethoxybenzylidene)imidazolidine-2,4-dione (SAI503)

Yield 274 mg (52%). Yellow solid. M.p. over 250°C.

^{1}H NMR (700 MHz, DMSO-*d*$_6$) δ 11.05 (brs, 1H), 10.32 (brs, 1H), 9.52 (brs, 1H), 7.04 (s, 1H), 6.62 (s, 1H), 6.53 (s, 1H), 3.81 (s, 3H), 3.75 (s, 3H).

^{13}C NMR (75 MHz, DMSO-*d*$_6$) δ 165.7, 155.7, 153.0, 149.2, 141.6, 125.3, 113.4, 111.7, 104.0, 100.3, 56.7, 56.0.

HRMS (ESI) m/z $[M+H]^+$ calcd for $C_{12}H_{13}N_2O_5$: 265.0819, found: 265.0818.

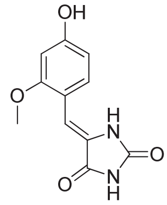

(Z)-5-(4-Hydroxy-2-methoxybenzylidene)imidazolidine-2.4-dione (SAI487)

Yield 84 mg (18%). Brown solid. M.p. 237-239°C.

^{1}H NMR (700 MHz, DMSO-*d*$_6$) δ 10.21 (brs, 3H), 7.45 (d, J = 8.4 Hz, 1H), 6.61 (s, 1H), 6.44 (d, J = 2.3 Hz, 1H), 6.39 (dd, J = 8.4, 2.3 Hz, 1H), 3.79 (s, 3H).

^{13}C NMR (176 MHz, DMSO-*d*$_6$) δ 165.6, 159.8, 158.8, 155.4, 130.3, 125.3, 112.6, 107.7, 103.7, 99.0, 55.5.

HRMS (ESI) m/z $[M+H]^+$ calcd for $C_{11}H_{11}N_2O_4$: 235.0713, found: 235.0715.

5-(4-hydroxybenzylidene)-thiazolidine-2,4-diones

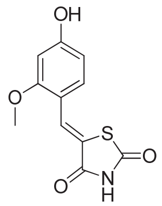

(Z)-5-(4-Hydroxy-2-methoxybenzylidene)thiazolidine-2,4-dione (SAI474)

Yield 452 mg (90%). Yellow solid.

^{1}H NMR (700 MHz, DMSO-d_6) δ 12.35 (brs, 1H), 10.36 (brs, 1H), 7.92 (s, 1H), 7.25 (d, J = 8.5 Hz, 1H), 6.53 (dd, J = 8.5, 2.3 Hz, 1H), 6.51 (d, J = 2.3 Hz, 1H), 3.83 (s, 3H).

Previously characterized. Spectral properties correspond to the previously reported.[5]

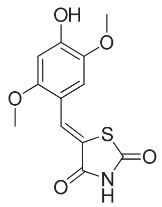

(Z)-5-(4-Hydroxy-2,5-dimethoxybenzylidene)thiazolidine-2,4-dione (SAI477)

Yield 472 mg (84%). Orange solid. M.p. over 250°C.

^{1}H NMR (700 MHz, DMSO-d_6) δ 12.37 (brs, 1H), 10.11 (s, 1H), 7.92 (s, 1H), 6.90 (s, 1H), 6.61 (s, 1H), 3.80 (s, 3H), 3.77 (s, 3H).

^{13}C NMR (176 MHz, DMSO-d_6) δ 168.1, 167.5, 154.4, 151.4, 142.0, 127.0, 118.4, 112.2, 111.5, 100.6, 56.2, 56.0.

HRMS (ESI) m/z $[M+H]^+$ calcd for $C_{12}H_{12}NO_5S$: 282.0431, found: 282.0430.

5-(Z)-benzylidene-2-methyl-3-alkyl/aryl-3,5-dihydro-4H-imidazol-4-ones

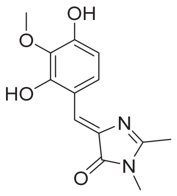

(*Z*)-5-(2,4-Dihydroxy-3-methoxybenzylidene)-2,3-dimethyl-3,5-dihydro-4H-imidazol-4-one (N1042)

Previously characterized. Spectral properties correspond to the previously reported.[2]

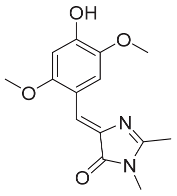

(*Z*)-5-(4-Hydroxy-2,5-dimethoxybenzylidene)-2,3-dimethyl-3,5-dihydro-4H-imidazol-4-one (N1036)

Previously characterized. Spectral properties correspond to the previously reported.[2]

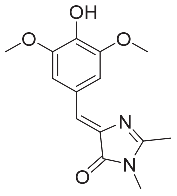

(*Z*)-5-(4-Hydroxy-3,5-dimethoxybenzylidene)-2,3-dimethyl-3,5-dihydro-4H-imidazol-4-one (N979)

Previously characterized. Spectral properties correspond to the previously reported.[2]

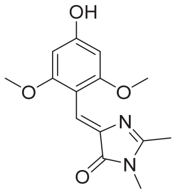

(*Z*)-5-(4-Hydroxy-2,6-dimethoxybenzylidene)-2,3-dimethyl-3,5-dihydro-4H-imidazol-4-one (N976)

Previously characterized. Spectral properties correspond to the previously reported.[2]

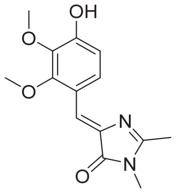

(*Z*)-5-(4-Hydroxy-2,3-dimethoxybenzylidene)-2,3-dimethyl-3,5-dihydro-4H-imidazol-4-one (N1027)

Previously characterized. Spectral properties correspond to the previously reported.[2]

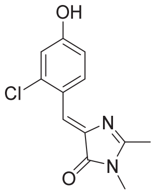

(*Z*)-5-(2-Chloro-4-hyroxybenzylidene)-2,3-dimethyl-3,5-dihydro-4H-imidazol-4-one (N1179)

Previously characterized. Spectral properties correspond to the previously reported.[2]

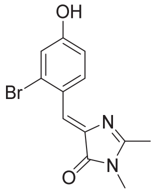

(*Z*)-5-(2-Bromo-4-hyroxybenzylidene)-2,3-dimethyl-3,5-dihydro-4H-imidazol-4-one (N1180)

Previously characterized. Spectral properties correspond to the previously reported.[2]

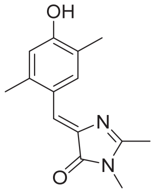

(*Z*)-5-(4-Hydroxy-2,5-dimethylbenzylidene)-2,3-dimethyl-3,5-dihydro-4H-imidazol-4-one (M2876)

Previously characterized. Spectral properties correspond to the previously reported.[2]

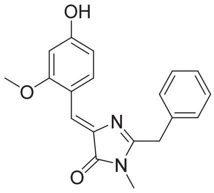

(Z)-2-Benzyl-5-(4-hydroxy-2-methoxybenzylidene)-3-methyl-3,5-dihydro-4H-imidazol-4-one (N1135)

Previously characterized. Spectral properties correspond to the previously reported.[7]

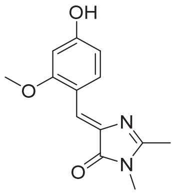

(Z)-5-(4-Hydroxy-2-methoxybenzylidene)-2,3-dimethyl-3,5-dihydro-4H-imidazol-4-one (N865)

Previously characterized. Spectral properties correspond to the previously reported.[8]

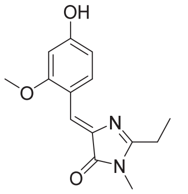

(Z)-5-(4-Hydroxy-2-methoxybenzylidene)-2-ethyl-3-methyl-3,5-dihydro-4H-imidazol-4-one (ZS362)

Previously characterized. Spectral properties correspond to the previously reported.[7]

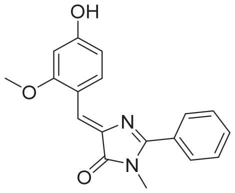

(Z)-5-(4-hydroxy-2-methoxybenzylidene)-3-methyl-2-phenyl-3,5-dihydro-4H-imidazol-4-one (N1131)

Orange solid (1.91 g. 62%). M. p. = 262-265 ºC.

^{1}H NMR (700 MHz, DMSO-d_6) δ ppm 10.32 (br. s.. 1 H), 8.76 (d, *J*=8.6 Hz, 1 H), 7.90 (d, *J*=6.9 Hz, 2 H), 7.57 - 7.63 (m. 3 H), 7.46 (s, 1 H), 6.51 (dd, *J*=8.8, 2.1 Hz, 1 H), 6.47 (d, *J*=2.1 Hz, 1 H), 3.86 (s, 3 H), 3.25 (s, 3 H).

^{13}C NMR (75 MHz, DMSO-d_6) δ ppm 170.6, 162.1, 160.8, 160.4, 135.4, 134.1, 131.2, 129.3, 128.8, 128.6, 120.9, 114.1, 108.8, 98.7, 55.7, 28.6.

HRMS (ESI) m/z: 309.1234 found (calcd for $C_{18}H_{17}N_2O_3^+$, $[M+H]^+$ 309.1234).

(Z)-2-(4-hydroxy-2-methoxybenzylidene)imidazo[1,2-a]pyridin-3(2H)-ones

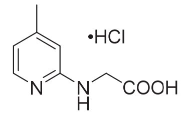

2-((4-methylpyridin-2-yl)amino)acetic acid hydrochloride

Synthetic routes and spectral characteristics were previously described.[7]

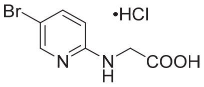

(5-Bromopyridin-2-yl)glycine hydrochloride

Synthetic routes and spectral characteristics were previously described.[9]

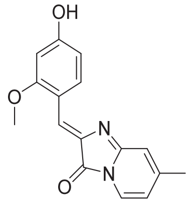

(Z)-2-(4-Hydroxy-2-methoxybenzylidene)-7-methylimidazo[1,2-a]pyridin-3(2H)-one (M3007a)

Previously characterized. Spectral properties correspond to the previously reported.[7]

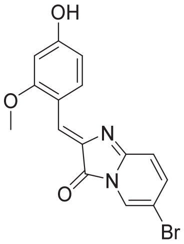

(Z)-6-Bromo-2-(4-hydroxy-2-methoxybenzylidene)imidazo[1,2-a]pyridin-3(2H)-one (MID323)

Red solid (0.2 g, 10%). M. p. ~ 285 ºC with decomposition.

^{1}H NMR (700 MHz, DMSO-d_6) δ ppm 10.52 (br. s., 1 H), 8.79 (d, *J*=8.8 Hz, 1 H), 7.97 (d, *J*=1.0 Hz, 1 H), 7.57 (s, 1 H), 7.32 (dd, *J*=9.8, 1.8 Hz, 1 H), 6.98 (d, *J*=9.7 Hz, 1 H), 6.53 (dd, *J*=8.7, 2.0 Hz, 1 H), 6.47 (d, *J*=2.1 Hz, 1 H), 3.87 (s, 3 H).

^{13}C NMR (176 MHz, DMSO-d_6) δ ppm 165.1, 163.0, 161.3, 151.9, 139.3, 134.7, 134.4, 126.0, 122.9, 120.0, 114.7, 109.1, 103.0, 98.7, 55.7.

HRMS (ESI) m/z: 347.0032 found (calcd for $C_{15}H_{12}BrN_2O_3^+$, $[M+H]^+$ 347.0026).

5-(Z)-benzylidene-2-(E)-arylvinyl-3-methyl-3,5-dihydro-4H-imidazol-4-ones

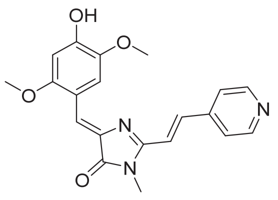

5-((Z)-4-Hydroxy-2,5-dimethoxybenzylidene)-3-methyl-2-((E)-2-(pyridin-4-yl)vinyl)-3,5-dihydro-4H-imidazol-4-one (N1048)

Previously characterized. Spectral properties correspond to the previously reported.[2]

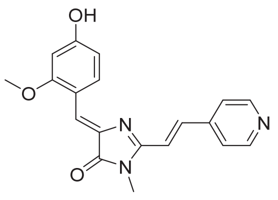

(Z)-5-(4-Hydroxy-2-methoxybenzylidene)-3-methyl-2-((E)-2(pyridine-4-yl)vinyl)-3,5-dihydro-4H-imidazol-4-one (N871b)

Previously characterized. Spectral properties correspond to the previously reported.[8]

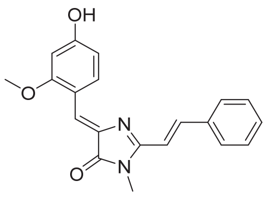

(Z)-5-(4-Hydroxy-2-methoxybenzylidene)-3-methyl-2-((E)-styryl)-3,5-dihydro-4H-imidazol-4-one (MID145)

Previously characterized. Spectral properties correspond to the previously reported.[7]

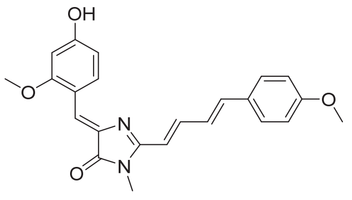

(Z)-5-(4-Hydroxy-2-methoxybenzylidene)-2-((1E,3E)-4-(4-methoxyphenyl)buta-1,3-dien-1-yl)-3-methyl-3,5-dihydro-4H-imidazol-4-one (N1052)

Previously characterized. Spectral properties correspond to the previously reported.[7]

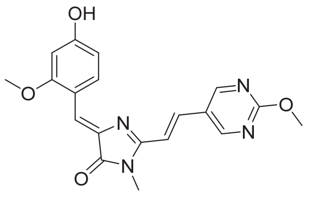

5-((Z)-4-Hydroxy-2-methoxybenzylidene)-2-((E)-2-(2-methoxypyrimidin-5-yl)vinyl)-3-methyl-3,5-dihydro-4H-imidazol-4-one (N1184)

Previously characterized. Spectral properties correspond to the previously reported.[7]

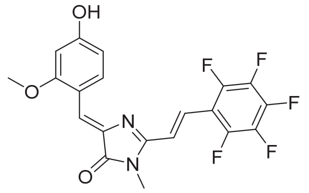

5-((Z)-4-Hydroxy-2-methoxybenzylidene)-3-methyl-2-((E)-2-(perfluorophenyl)vinyl)-3,5-dihydro-4H-imidazol-4-one (N1049)

Previously characterized. Spectral properties correspond to the previously reported.[7]

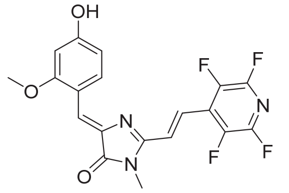

5-((Z)-4-Hydroxy-2-methoxybenzylidene)-3-methyl-2-((E)-2-(perfluoropyridin-4-yl)vinyl)-3,5-dihydro-4H-imidazol-4-one (N967)

Previously characterized. Spectral properties correspond to the previously reported.[7]

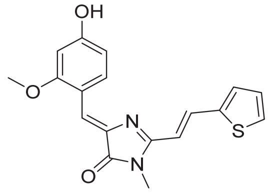

5-((Z)-4-Hydroxy-2-methoxybenzylidene)-3-methyl-2-((E)-2-(thiophen-2-yl)vinyl)-3,5-dihydro-4H-imidazol-4-one (N1039)

Previously characterized. Spectral properties correspond to the previously reported.[7]

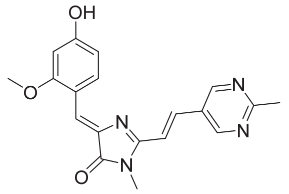

5-((Z)-4-Hydroxy-2-methoxybenzylidene)-3-methyl-2-((E)-2-(2-methylpyrimidin-5-yl)vinyl)-3,5-dihydro-4H-imidazol-4-one (N1202)

Previously characterized. Spectral properties correspond to the previously reported.[7]

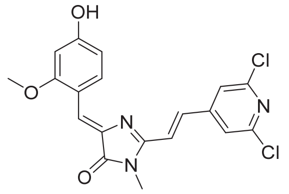

5-((Z)-4-Hydroxy-2-methoxybenzylidene)-2-((E)-2-(2,6-dichloropyridin-4-yl)vinyl)-3-methyl-3,5-dihydro-4H-imidazol-4-one (N960b)

Previously characterized. Spectral properties correspond to the previously reported.[7]

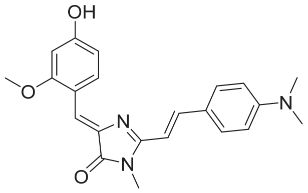

5-((Z)-4-Hydroxy-2-methoxybenzylidene)-3-methyl-2-((E)-4-(dimethylamino)styryl)-3,5-dihydro-4H-imidazol-4-one (MID147)

Previously characterized. Spectral properties correspond to the previously reported.[7]

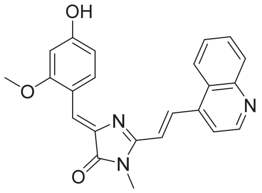

5-((Z)-4-Hydroxy-2-methoxybenzylidene)-3-methyl-2-((E)-2-(quinolin-4-yl)vinyl)-3,5-dihydro-4H-imidazol-4-one (ZS331)

Previously characterized. Spectral properties correspond to the previously reported.[7]

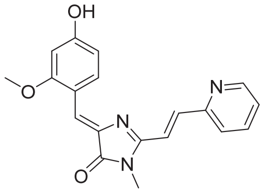

5-((Z)-4-Hydroxy-2-methoxybenzylidene)-3-methyl-2-((E)-2-(pyridin-2-yl)vinyl)-3,5-dihydro-4H-imidazol-4-one (M2766)

Previously characterized. Spectral properties correspond to the previously reported.[7]

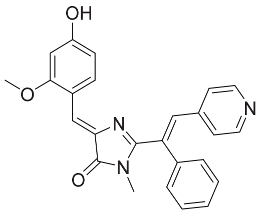

5-((Z)-4-Hydroxy-2-methoxybenzylidene)-3-methyl-2-((E)-1-phenyl-2-(pyridin-4-yl)vinyl)-3,5-dihydro-4H-imidazol-4-one (N1142)

Previously characterized. Spectral properties correspond to the previously reported.[7]

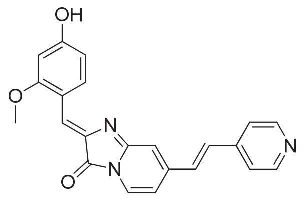

(Z)-2-(4-Hydroxy-2-methoxybenzylidene)-7-((E)-2-(pyridin-4-yl)vinyl)imidazo[1,2-a]pyridin-3(2H)-one (MID343)

Previously characterized. Spectral properties correspond to the previously reported.[7]

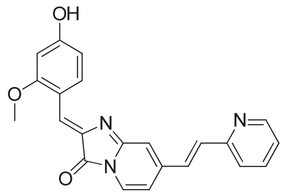

(Z)-2-(4-Hydroxy-2-methoxybenzylidene)-7-((E)-2-(pyridin-2-yl)vinyl)imidazo[1,2-a]pyridin-3(2H)-one (MID367)

Previously characterized. Spectral properties correspond to the previously reported.[7]

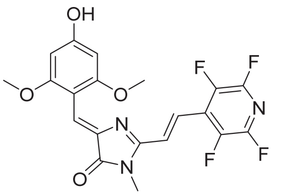

5-((Z)-4-Hydroxy-2,6-dimethoxybenzylidene)-3-methyl-2-((E)-2-(perfluoropyridin-4-yl)vinyl)-3,5-dihydro-4H-imidazol-4-one (N971)

Previously characterized. Spectral properties correspond to the previously reported.[2]

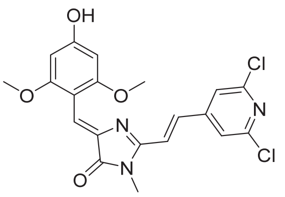

5-((Z)-4-Hydroxy-2,6-dimethoxybenzylidene)-2-((E)-2-(2,6-dichloropyridin-4-yl)vinyl)-3-methyl-3,5-dihydro-4H-imidazol-4-one (N973)

Previously characterized. Spectral properties correspond to the previously reported.[2]

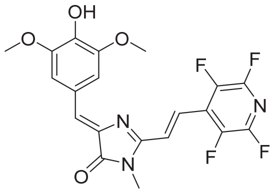

5-((Z)-4-Hydroxy-3,5-dimethoxybenzylidene)-2-((E)-2-(perfluoropyridin-4-yl)vinyl)-3-methyl-3,5-dihydro-4H-imidazol-4-one (N980)

Previously characterized. Spectral properties correspond to the previously reported.[2]

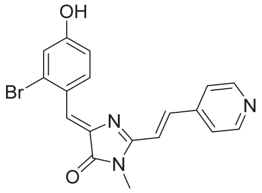

5-((Z)-2-Bromo-4-hydroxybenzylidene)-3-methyl-2-((E)-2-(pyridine-4-yl)vinyl)-3,5-dihydro-4H-imidazol-4-one (N1205)

Previously characterized. Spectral properties correspond to the previously reported.[2]

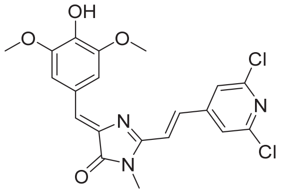

5-((Z)-4-Hydroxy-3,5-dimethoxybenzylidene)-2-((E)-2-(2,6-dichloropyridin-4-yl)vinyl)-3-methyl-3,5-dihydro-4H-imidazol-4-one (N960a)

Previously characterized. Spectral properties correspond to the previously reported.[2]

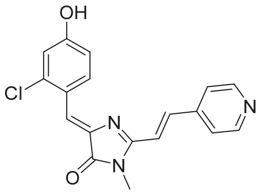

5-((Z)-2-Chloro-4-hydroxybenzylidene)-3-methyl-2-((E)-2-(pyridine-4-yl)vinyl)-3,5-dihydro-4H-imidazol-4-one (N1204)

Previously characterized. Spectral properties correspond to the previously reported.[2]

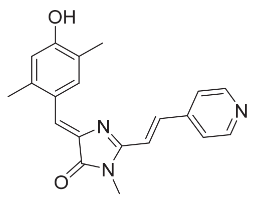

5-((Z)-4-Hydroxy-2,5-dimethylbenzylidene)-3-methyl-2-((E)-2-(pyridine-4-yl)vinyl)-3,5-dihydro-4H-imidazol-4-one (N1206)

Previously characterized. Spectral properties correspond to the previously reported.[2]

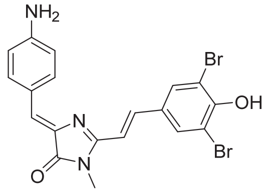

5-((Z)-4-Aminobenzylidene)-2-((E)-3,5-dibromo-4-hyrdoxystyryl)-3-methyl-3,5-dihydro-4H-imidazol-4-one (ZS309)

Previously characterized. Spectral properties correspond to the previously reported.[2]

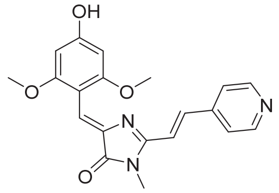

5-((Z)-4-Hydroxy-2,6-dimethoxybenzylidene)-3-methyl-2-((E)-2-(pyridin-4-yl)vinyl)-3,5-dihydro-4H-imidazol-4-one (ZS316)

Previously characterized. Spectral properties correspond to the previously reported.[2]

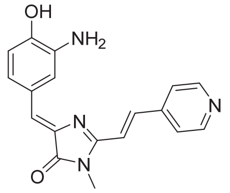

5-((Z)-3-Amino-4-hydroxybenzylidene)-3-methyl-2-((E)-2-(pyridin-4-yl)vinyl)-3,5-dihydro-4H-imidazol-4-one (ZS325)

Previously characterized. Spectral properties correspond to the previously reported.[2]

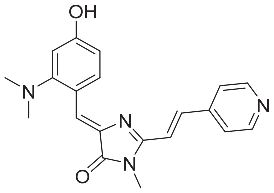

5-((Z)-2-(Dimethylamino)-4-hydroxybenzylidene)-3-methyl-2-((E)-2-(pyridin-4-yl)vinyl)-3,5-dihydro-4H-imidazol-4-one (MID213)

Previously characterized. Spectral properties correspond to the previously reported.[2]

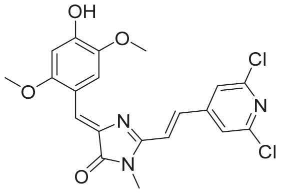

5-((Z)-4-Hydroxy-2,5-dimethoxybenzylidene)-3-methyl-2-((E)-2-(2,6-dichloropyridin-4-yl)vinyl)-3,5-dihydro-4H-imidazol-4-one (M2767)

Previously characterized. Spectral properties correspond to the previously reported.[2]

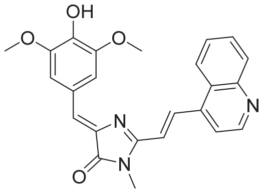

5-((*Z*)-4-Hydroxy-3,5-dimethoxybenzylidene)-3-methyl-2-((*E*)-2-(quinolin-4-yl)vinyl)-3,5-dihydro-4*H*-imidazol-4-one (N1196)

Previously characterized. Spectral properties correspond to the previously reported.[10]

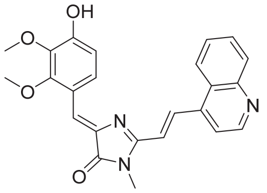

5-((*Z*)-4-Hydroxy-2,3-dimethoxybenzylidene)-3-methyl-2-((*E*)-2-(quinolin-4-yl)vinyl)-3,5-dihydro-4*H*-imidazol-4-one (N1197)

Previously characterized. Spectral properties correspond to the previously reported.[10]

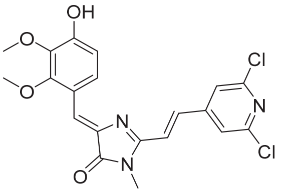

2-((*E*)-2-(2,6-dichloropyridin-4-yl)vinyl)-5-((*Z*)-4-hydroxy-2,3-dimethoxybenzylidene)-3-methyl-3,5-dihydro-4*H*-imidazol-4-one (N1198)

Previously characterized. Spectral properties correspond to the previously reported.[10]

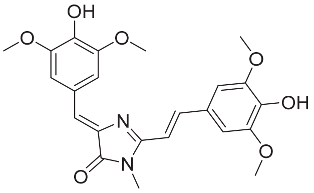

5-((*Z*)-4-hydroxy-3,5-dimethoxybenzylidene)-2-((*E*)-4-hydroxy-3,5-dimethoxystyryl)-3-methyl-3,5-dihydro-4*H*-imidazol-4-one (N1193)

Previously characterized. Spectral properties correspond to the previously reported.[10]

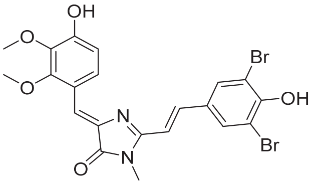

2-((*E*)-3,5-dibromo-4-hydroxystyryl)-5-((*Z*)-4-hydroxy-2,3-dimethoxybenzylidene)-3-methyl-3,5-dihydro-4*H*-imidazol-4-one (N1199)

Previously characterized. Spectral properties correspond to the previously reported.[10]

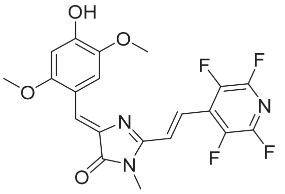

5-((Z)-4-Hydroxy-2,5-dimethoxybenzylidene)-3-methyl-2-((E)-2-(perfluoropyridin-4-yl)vinyl)-3,5-dihydro-4H-imidazol-4-one (N1056)

Previously characterized. Spectral properties correspond to the previously reported.[2]

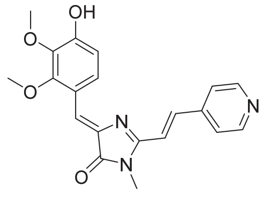

5-((Z)-4-Hydroxy-2,3-dimethoxybenzylidene)-3-methyl-2-((E)-2-(pyridin-4-yl)vinyl)-3,5-dihydro-4H-imidazol-4-one (N1068)

Previously characterized. Spectral properties correspond to the previously reported.[2]

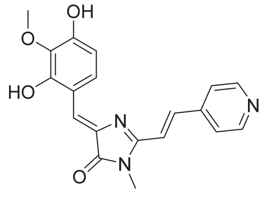

5-((Z)-2,4-Dihydroxy-3-methoxybenzylidene)-3-methyl-2-((E)-2-(pyridin-4-yl)vinyl)-3,5-dihydro-4H-imidazol-4-one (N1069)

Previously characterized. Spectral properties correspond to the previously reported.[2]

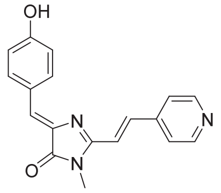

5-((Z)-4-Hydroxybenzylidene)-3-methyl-2-((E)-2-(pyridin-4-yl)vinyl)-3,5-dihydro-4H-imidazol-4-one (A9)

Previously characterized. Spectral properties correspond to the previously reported.[8]

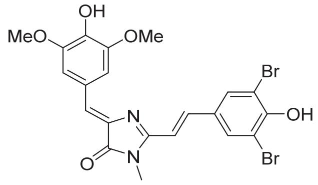

2-((E)-3,5-dibromo-4-hydroxystyryl)-5-((Z)-4-hydroxy-3,5-dimethoxybenzylidene)-3-methyl-3,5-dihydro-4H-imidazol-4-one (Z260)

Previously characterized. Spectral properties correspond to the previously reported.[8]

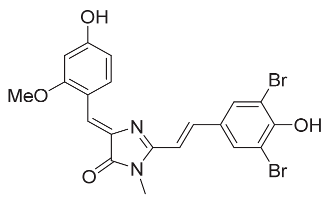

2-((E)-3,5-dibromo-4-hydroxystyryl)-5-((Z)-4-hydroxy-2-methoxybenzylidene)-3-methyl-3,5-dihydro-4H-imidazol-4-one (N871a)

Previously characterized. Spectral properties correspond to the previously reported.[8]

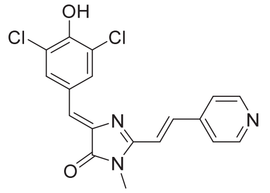

5-((Z)-3,5-dichloro-4-hydroxybenzylidene)-3-methyl-2-((E)-2-(pyridin-4-yl)vinyl)-3,5-dihydro-4H-imidazol-4-one (N901)

Previously characterized. Spectral properties correspond to the previously reported.[8]

Other compounds

Several compounds were prepeared in accordnce with previously reported procedures (see references below) or were taken from stock of our laboratory.

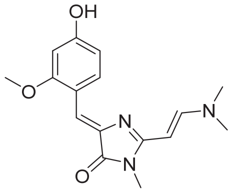

5-((Z)-4-Hydroxy-2-methoxybenzylidene)-3-methyl-2-((E)-2-(dimethylamino)vinyl)-3,5-dihydro-4H-imidazol-4-one (N1122)

Synthetic routes and spectral characteristics were previously described.[7]

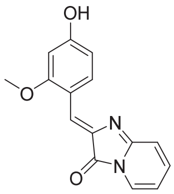

(Z)-2-(4-Hydroxy-2-methoxybenzylidene)imidazo[1,2-a]pyrydin-3(2H)-one (N1123)

Synthetic routes and spectral characteristics were previously described.[7]

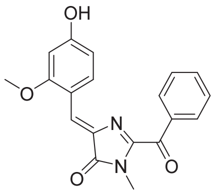

(Z)-2-Benzoyl-5-(4-hydroxy-2-methoxybenzylidene)-3-methyl-3,5-dihydro-4H-imidazol-4-one (N1139)

Synthetic routes and spectral characteristics were previously described.[7]

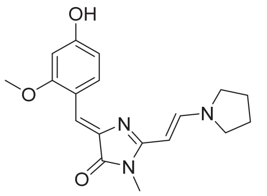

5-((Z)-4-Hydroxy-2-methoxybenzylidene)-3-methyl-2-((E)-2-(pyrrolidin-1-yl)vinyl)-3,5-dihydro-4H-imidazol-4-one (N1118)

Synthetic routes and spectral characteristics were previously described.[7]

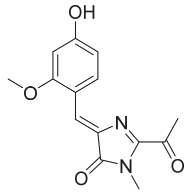

(Z)-2-Acetyl-5-(4-hydroxy-2-methoxybenzylidene)-3-methyl-3,5-dihydro-4H-imidazol-4-one (N1124)

Synthetic routes and spectral characteristics were previously described.[7]

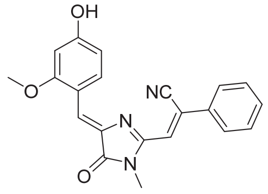

(Z)-3-(4-((Z)-4-Hydroxy-2-methoxybenzylidene)-1-methyl-5-oxo-4.5-dihydro-1H-imidazol-2-yl)-2-phenylacrylonitrile (MID151)

Synthetic routes and spectral characteristics were previously described.[7]

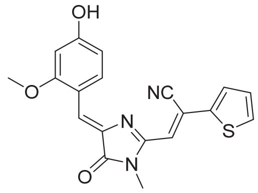

(Z)-3-(4-((Z)-4-Hydroxy-2-methoxybenzylidene)-1-methyl-5-oxo-4.5-dihydro-1H-imidazol-2-yl)-2-(thiophen-2-yl)acrylonitrile (MID153)

Synthetic routes and spectral characteristics were previously described.[7]

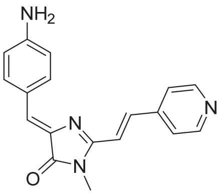

5-((Z)-4-Aminobenzylidene)-3-methyl-2-((E)-2-(pyrydin-4-yl)vinyl)-3,5-dihydro-4H-imidazol-4-one (ZS319)

Synthetic routes and spectral characteristics were previously described.[2]

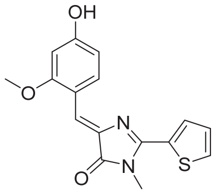

(*Z*)-5-(4-Hydroxy-2-methoxylbenzylidene)-3-methyl-2-(thiophene-2-yl)-3,5-dihydro-4H-imidazol-4-one (SH16)

Synthetic routes and spectral characteristics were previously described.[11]

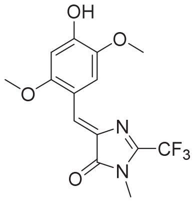

(Z)-5-(4-Hydroxy-2,5-dimethoxybenzylidene)-3-methyl-2-(trifluoromethyl)-3,5-dihydro-4H-imidazol-4-one (M2738d)

Synthetic routes and spectral characteristics were previously described.[12]

8. References

1. Würth C., Grabolle M., Pauli J., Spieles M., Resch-Genger U. Relative and Absolute Determination of Fluorescence Quantum Yields of Transparent Samples. *Nature Protocols.* **2013**, 8(8), 1535.
2. Mineev K.S., Goncharuk S.A., Goncharuk M.V., Povarova N.V., Sokolov A.I., Baleeva N.S., Smirnov A.Y., Myasnyanko I.N., Ruchkin D.A., Bukhdruker S., Remeeva A., Mishin A., Borshchevskiy V., Gordeliy V., Arseniev A.S., Gorbachev D.A., Gavrikov A.S., Mishin A.S., Baranov M.S. NanoFAST: Structure-Based Design of a Small Fluorogen-Activating Protein with Only 98 Amino Acids. *Chem. Sci.* **2021**, 12(19), 6719.
3. Plamont M. A., Billon-Denis R., Maurin S., Gauron C., Pimenta F. M., Specht C. G., Shi J., Qurard J., Pan B., Rossignol J., Moncoq K., Morellet N., Volovitch M., Lescop E., Chen Y., Triller A., Vriz S., Saux T., Jullien L., Gautier A. Small Fluorescence-Activating and Absorption-Shifting Tag for Tunable Protein Imaging In Vivo. *Proc. Natl. Acad. Sci. USA* **2016**, 113, 497.
4. Li C., Plamont M. A., Sladitschek H. L., Rodrigues V., Aujard I., Neveu P., Saux T., Jullien L., Gautier, A. Dynamic multicolor protein labeling in living cells. *Chem. Sci.* **2017**, 8, 5598.
5. Wang, Z., Liu, Z., Lee, W., Kim, S-N., Yoon, G., Cheon, S.H. Design, synthesis and docking study of 5-(substituted benzylidene)thiazolidine-2,4-dione derivatives as inhibitors of protein tyrosine phosphatase 1B. *Bioorg. Med. Chem. Lett.* **2014**, 24 (15), 3337.
6. Baldridge A., Kowalik J., Tolbert L. M. Efficient Synthesis of New 4-Arylideneimidazolin-5-Ones Related to the GFP Chromophore by 2+3 Cyclocondensation of Arylideneimines with Imidate Ylides. *Synthesis*. **2010**, 14, 2424.
7. Myasnyanko I.N., Gavrikov A.S., Zaitseva S.O., Smirnov A.Y., Zaitseva E.R., Sokolov A.I., Malyshevskaya K.K., Baleeva N.S., Mishin A.S., Baranov M.S. Study of the Position of the Conjugated Substitute Influence on the Optical Properties of the Kaede Protein Chromophore Derivatives. *Chem. Eur. J.* **2021**, 27, 3986
8. Povarova N.V., Zaitseva S.O., Baleeva N.S., Smirnov A.Yu., Myasnyanko I.N., Zagudaylova M.B., Bozhanova N.G., Gorbachev D.A., Malyshevskaya K.K., Gavrikov A.S., Mishin A.S., Baranov M.S. Red-Shifted Substrates for FAST Fluorogen-Activating Protein Based on the GFP-Like Chromophores. *Chem. Eur. J.* **2019**, 25 (41), 9592.
9. Myasnyanko I.N., Baleeva N.S., Baranov M.S. Study of the Position of the Conjugated Substitute Influence on the Optical Properties of the Kaede Protein Chromophore Derivatives. *Russ. J. Bioorg. Chem.,* **2022**, 48, 110.
10. Ryazantsev D.Y., Myshkin M.Y., Alferova V.A., Tsvetkov V.B., Shustova E.Y., Kamzeeva P.N., Kovalets P.V., Zaitseva E.R., Baleeva N.S., Zatsepin T.S., Shenkarev Z.O., Baranov M.S., Kozlovskaya L.I., Aralov A.V. Probing GFP Chromophore Analogs as Anti-HIV Agents Targeting LTR-III G-Quadruplex. *Biomolecules*. **2021**, 11 (10), 1409.
11. Baleeva N.S., Smirnov A.Y., Myasnyanko I.N., Gavrikov A.S., Baranov M.S. Thiophene Analog of the GFP Chromophore As Fluorogen for FAST Protein. *Russ. J. Bioorg. Chem.* **2021**, 47(5), 1118.
12. Chen C., Tachibana S.R., Baleeva N.S., Myasnyanko I.N., Bogdanov A.M., Gavrikov A.S., Mishin A.S., Malyshevskaya K.K., Baranov M.S., Fang C. Developing Bright Green Fluorescent Protein (GFP)-like Fluorogens for Live-Cell Imaging with Nonpolar Protein-Chromophore Interactions. *Chemistry A European Journal.* **2021**, 27(35), 8946.

9. Copies of 1H and 13C NMR spectra

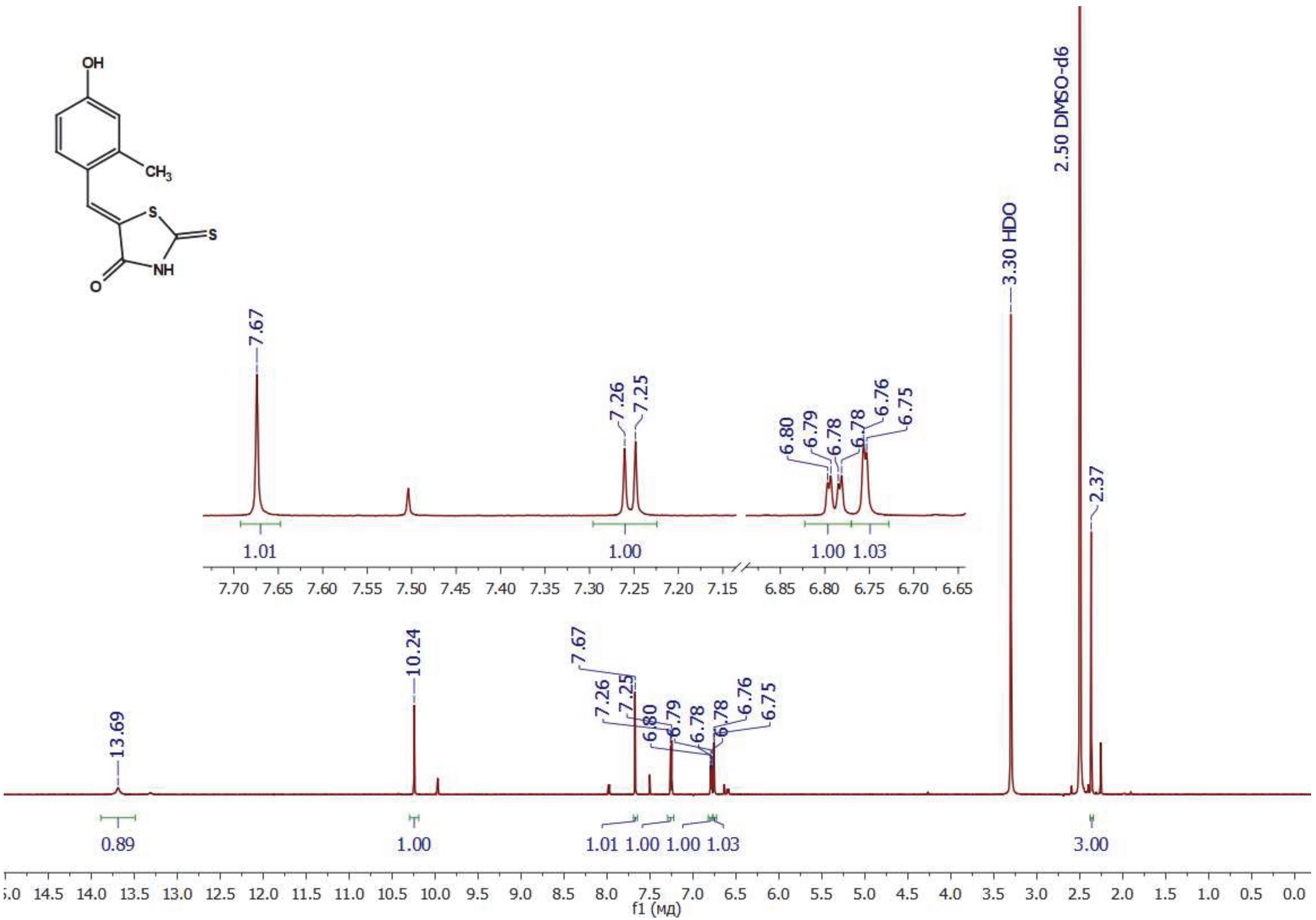

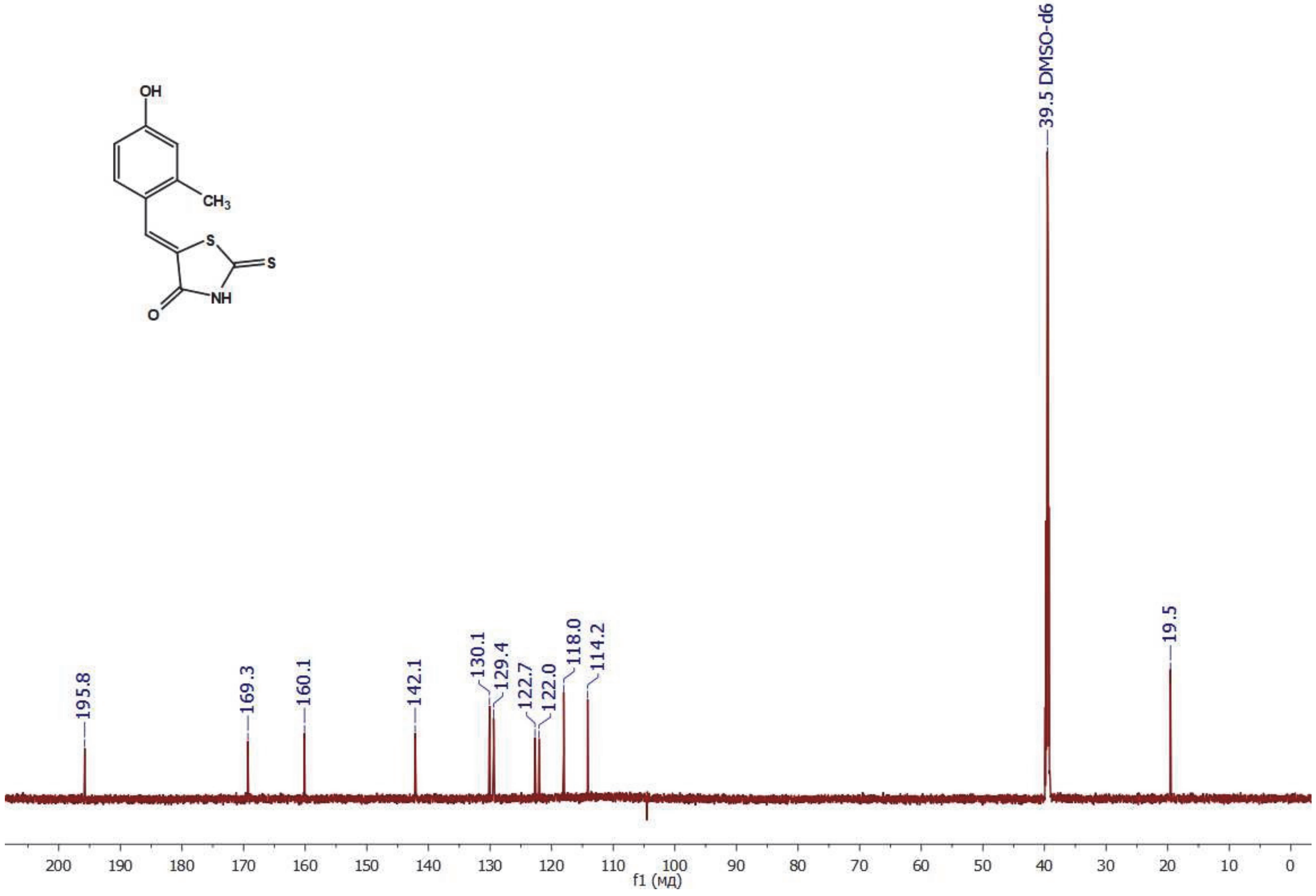
OH
CH3
S
S
NH
O
195.8
169.3
160.1
142.1
130.1
129.4
122.7
122.0
118.0
114.2
39.5 DMSO-d6
19.5
200
190
180
170
160
150
140
130
120
110
100
90
80
70
60
50
40
30
20
10
0
f1 (мд)

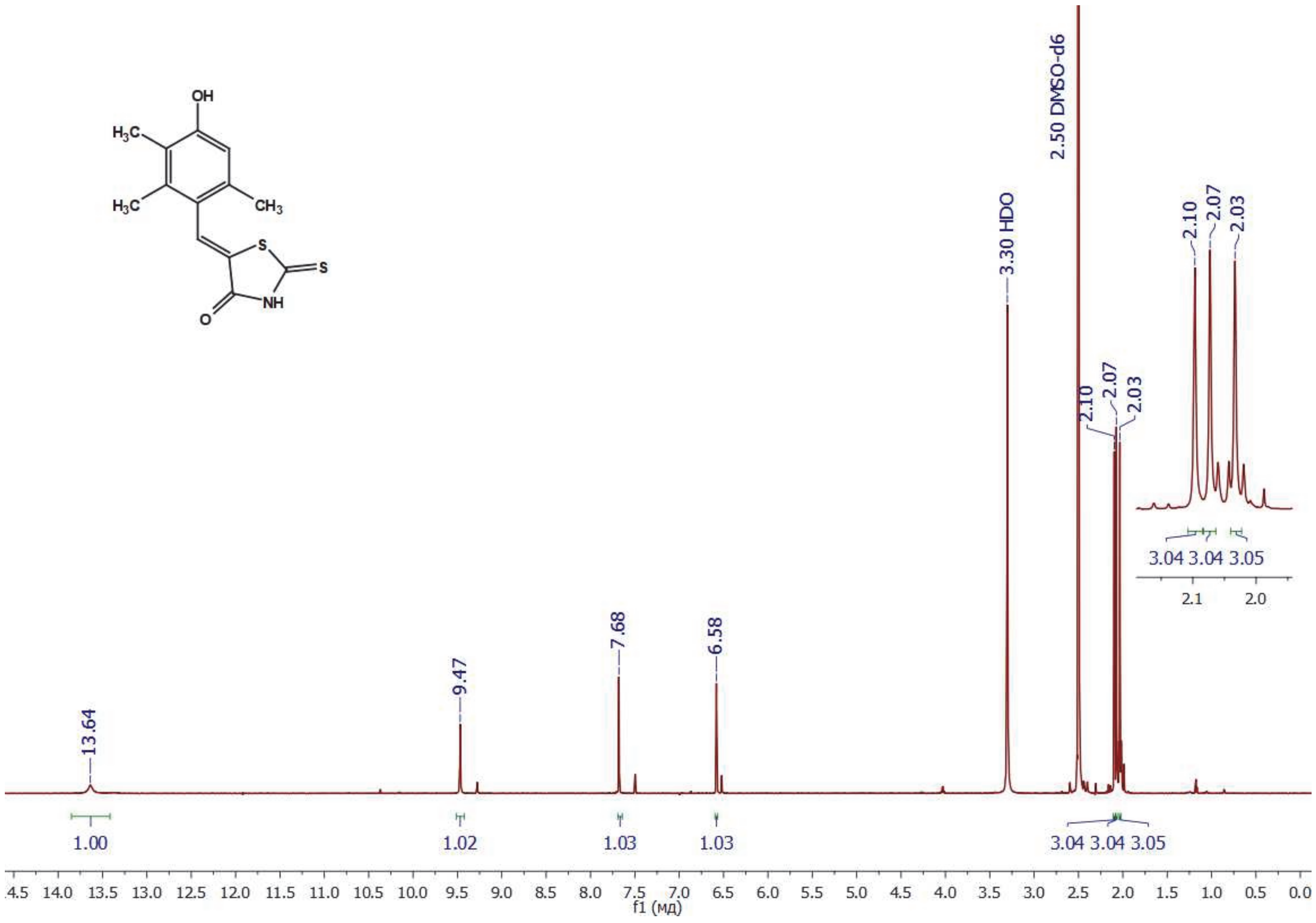
OH
H3C
H3C
CH3
S
S
NH
O
13.64
9.47
7.68
6.58
3.30 HDO
2.50 DMSO-d6
2.10
2.07
2.03
3.04 3.04 3.05
2.1
2.0
1.00
1.02
1.03
1.03
3.04 3.04 3.05
4.5 14.0 13.5 13.0 12.5 12.0 11.5 11.0 10.5 10.0 9.5 9.0 8.5 8.0 7.5 7.0 6.5 6.0 5.5 5.0 4.5 4.0 3.5 3.0 2.5 2.0 1.5 1.0 0.5 0.0
f1 (мд)

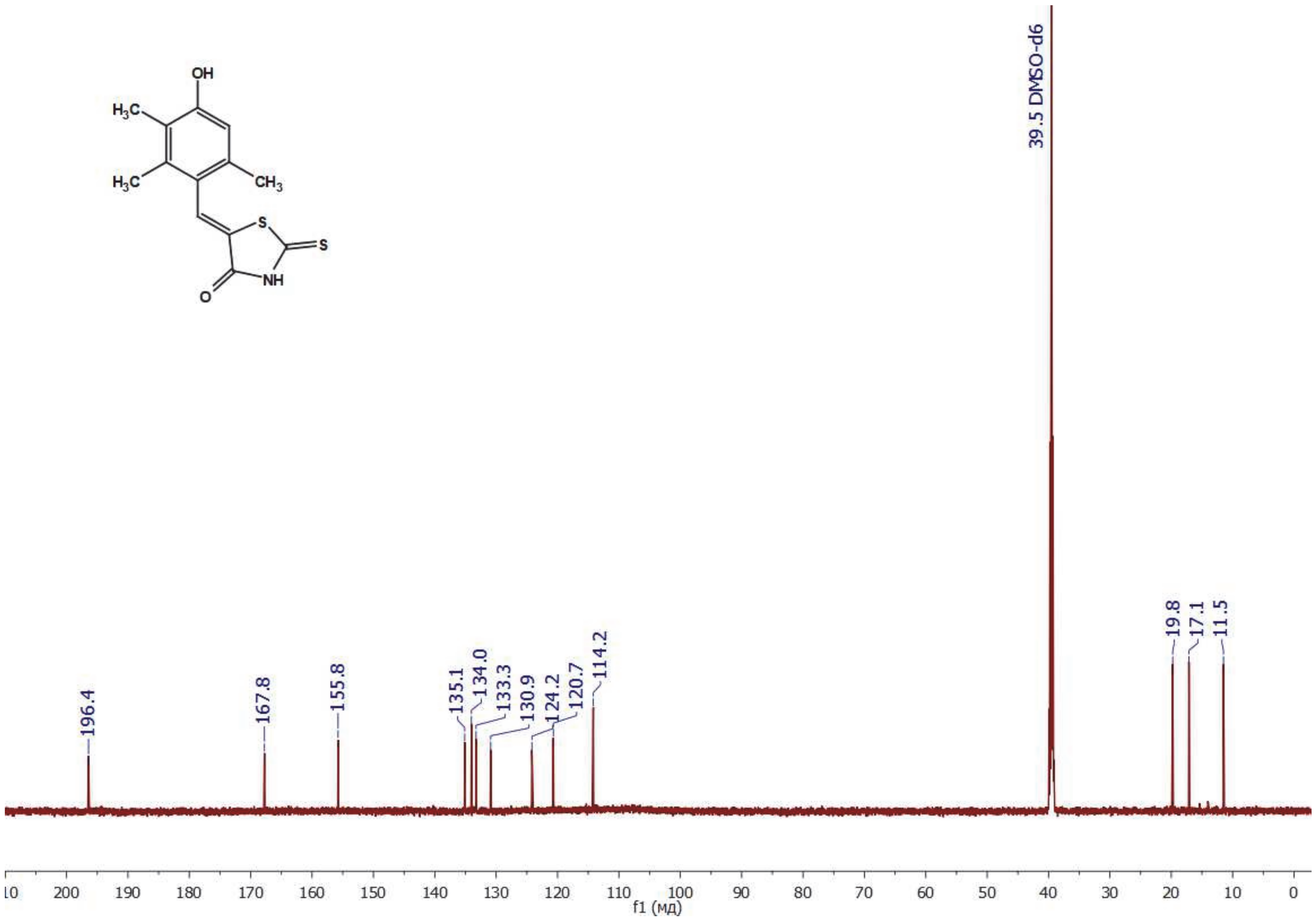
OH
H3C
H3C
CH3
S
S
NH
O
39.5 DMSO-d6
196.4
167.8
155.8
135.1
134.0
133.3
130.9
124.2
120.7
114.2
19.8
17.1
11.5
f1 (мд)

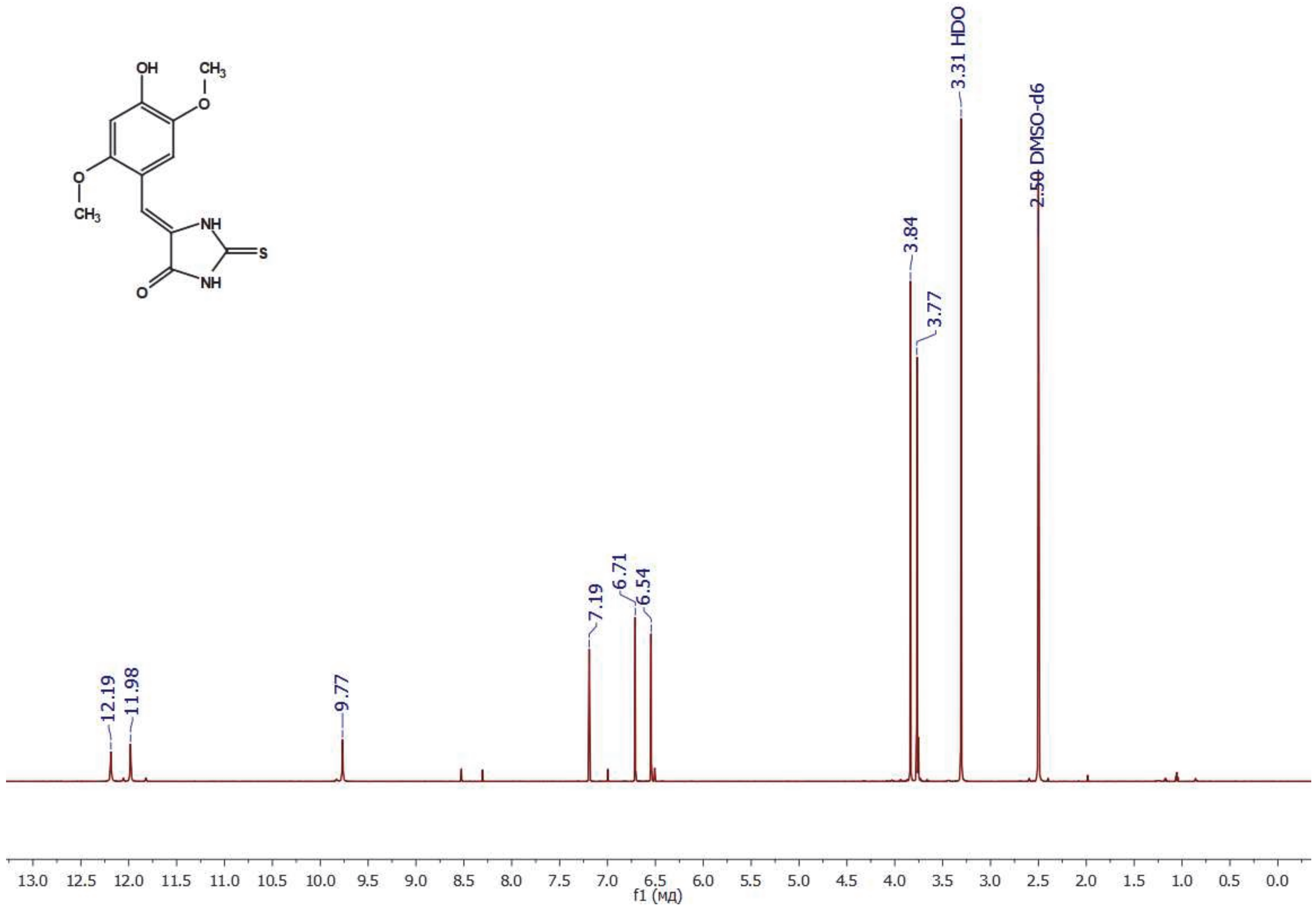
OH
CH3
O
O
CH3
NH
S
NH
O
12.19
11.98
9.77
7.19
6.71
6.54
3.84
3.77
3.31 HDO
2.50 DMSO-d6
13.0 12.5 12.0 11.5 11.0 10.5 10.0 9.5 9.0 8.5 8.0 7.5 7.0 6.5 6.0 5.5 5.0 4.5 4.0 3.5 3.0 2.5 2.0 1.5 1.0 0.5 0.0
f1 (мд)

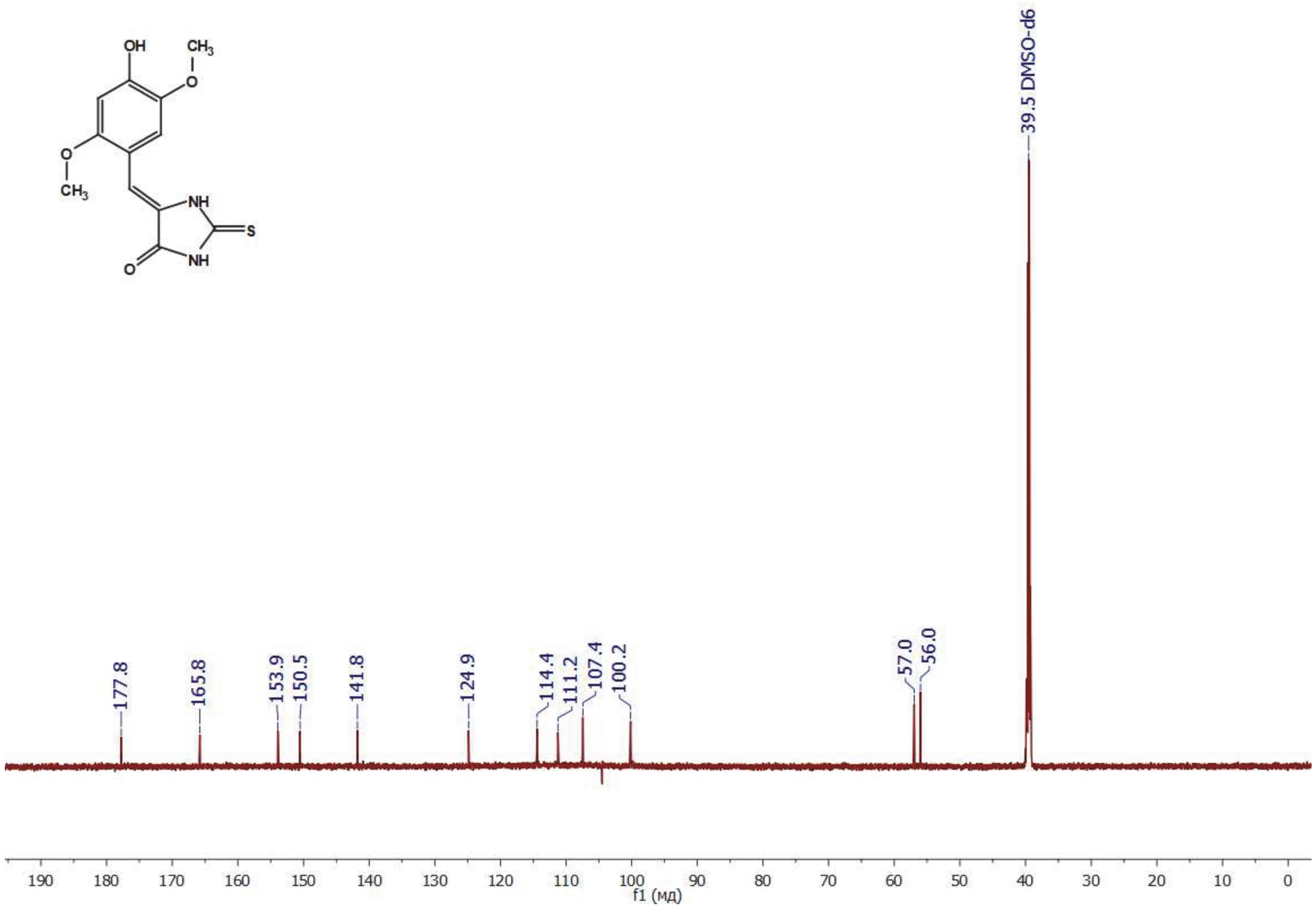
OH
CH3
O
O
CH3
NH
S
NH
O
39.5 DMSO-d6
177.8
165.8
153.9
150.5
141.8
124.9
114.4
111.2
107.4
100.2
57.0
56.0
190
180
170
160
150
140
130
120
110
100
90
80
70
60
50
40
30
20
10
0
f1 (мд)

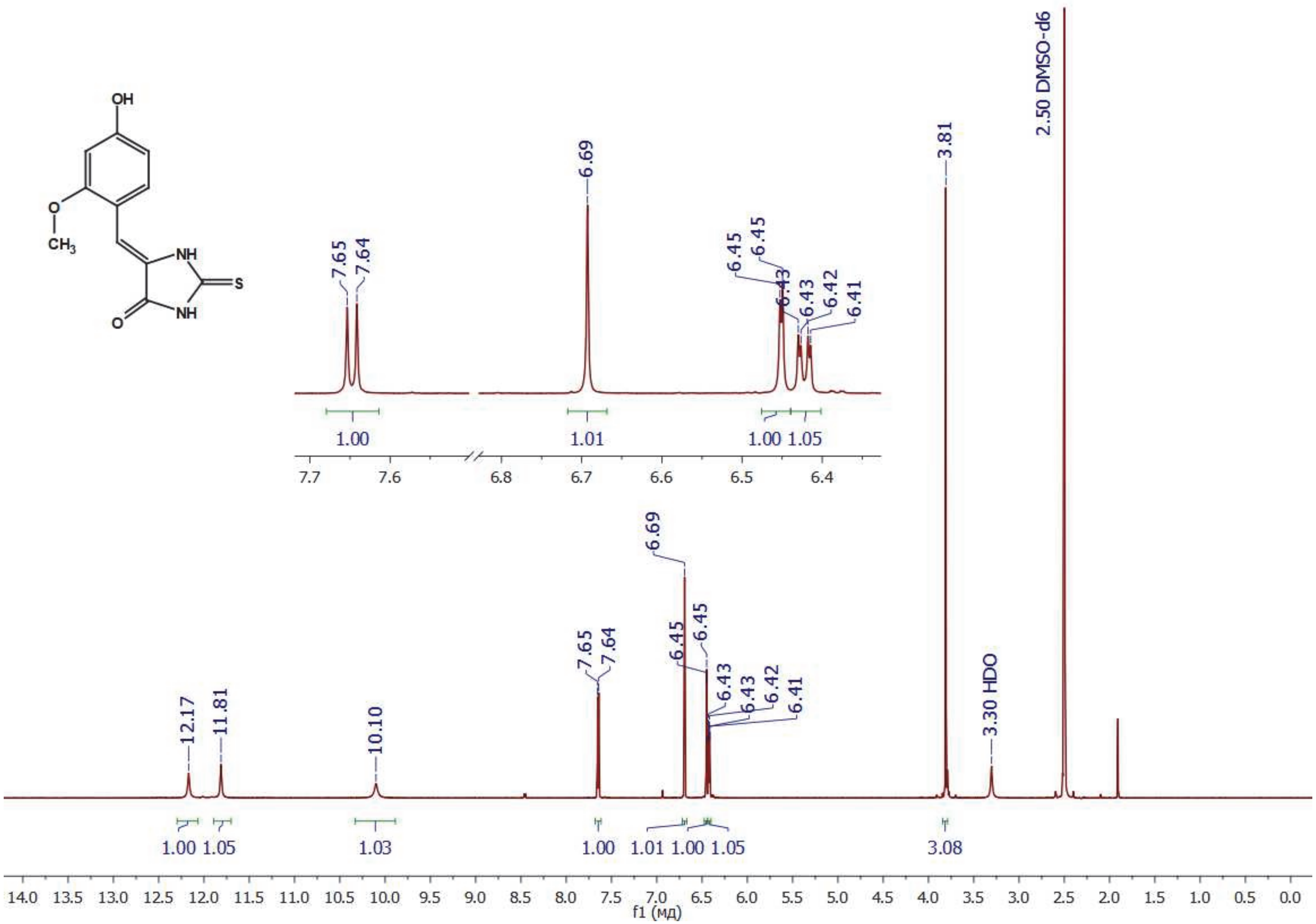

OH
O
CH3
NH
S
NH
O
7.65
7.64
6.69
6.45
6.45
6.43
6.43
6.42
6.41
1.00
1.01
1.00 1.05
7.7 7.6 6.8 6.7 6.6 6.5 6.4
12.17
11.81
10.10
7.65
7.64
6.69
6.45
6.45
6.43
6.43
6.42
6.41
3.81
3.30 HDO
2.50 DMSO-d6
1.00 1.05
1.03
1.00
1.01 1.00 1.05
3.08
14.0 13.5 13.0 12.5 12.0 11.5 11.0 10.5 10.0 9.5 9.0 8.5 8.0 7.5 7.0 6.5 6.0 5.5 5.0 4.5 4.0 3.5 3.0 2.5 2.0 1.5 1.0 0.5 0.0
f1 (мд)

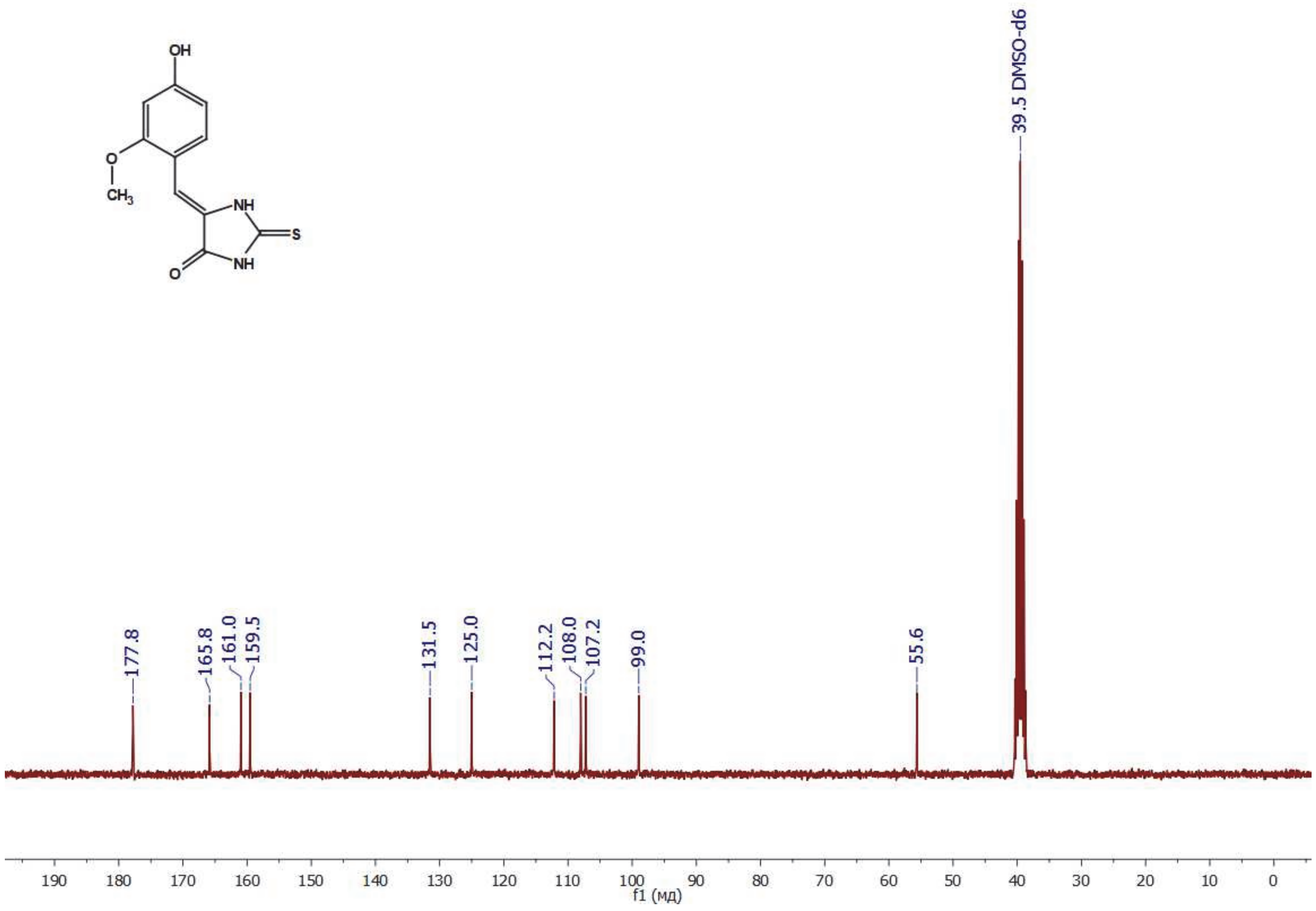

OH
O
CH3
NH
S
NH
O
177.8
165.8
161.0
159.5
131.5
125.0
112.2
108.0
107.2
99.0
55.6
39.5 DMSO-d6
190
180
170
160
150
140
130
120
110
100
90
80
70
60
50
40
30
20
10
0
f1 (мд)

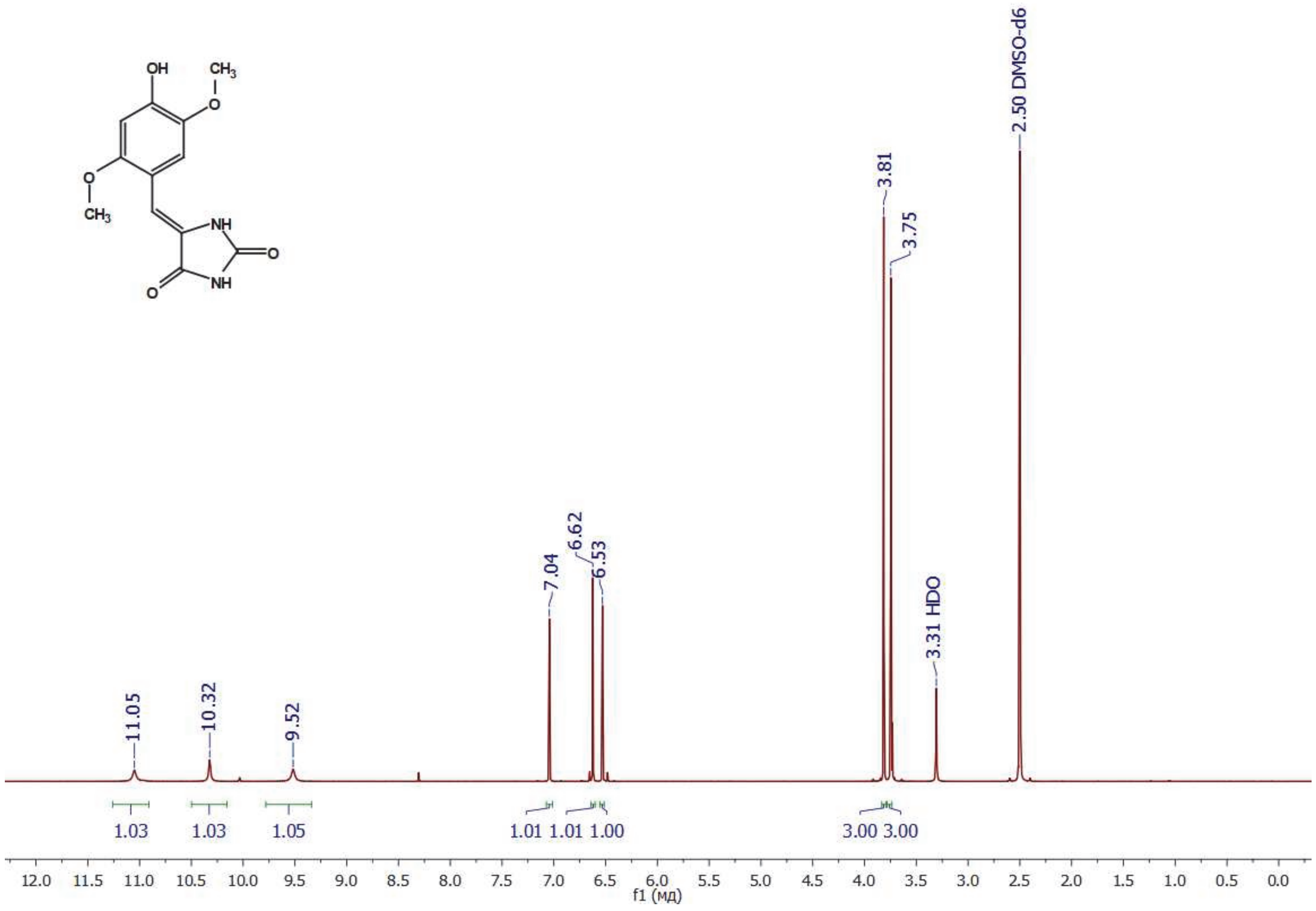
OH
CH3
O
O
CH3
NH
O
NH
O
11.05
10.32
9.52
7.04
6.62
6.53
3.81
3.75
3.31 HDO
2.50 DMSO-d6
1.03
1.03
1.05
1.01 1.01 1.00
3.00 3.00
12.0 11.5 11.0 10.5 10.0 9.5 9.0 8.5 8.0 7.5 7.0 6.5 6.0 5.5 5.0 4.5 4.0 3.5 3.0 2.5 2.0 1.5 1.0 0.5 0.0
f1 (мд)

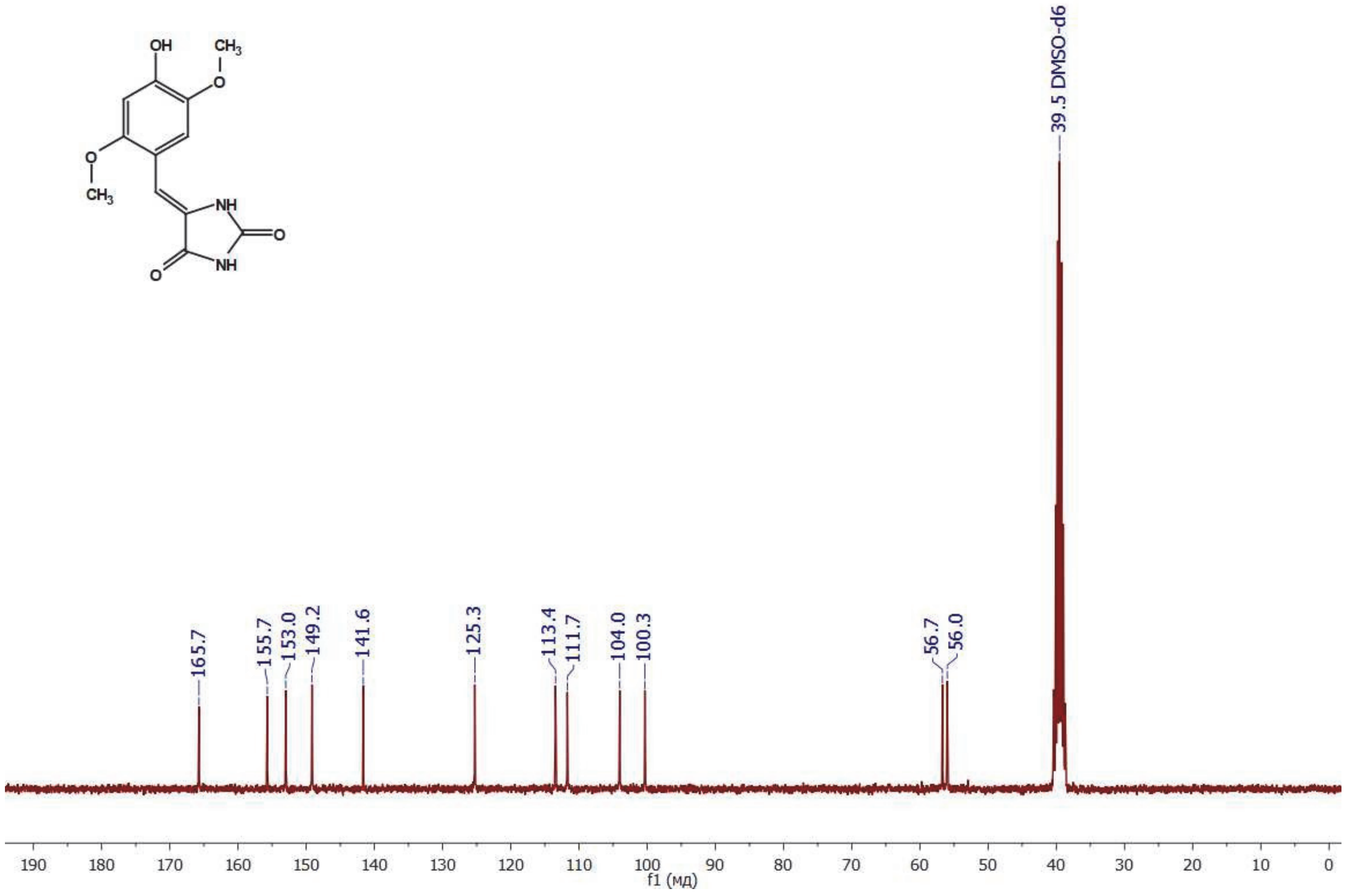

OH
CH3
O
O
CH3
NH
O
NH
O
165.7
155.7
153.0
149.2
141.6
125.3
113.4
111.7
104.0
100.3
56.7
56.0
39.5 DMSO-d6
190
180
170
160
150
140
130
120
110
100
90
80
70
60
50
40
30
20
10
0
f1 (мд)

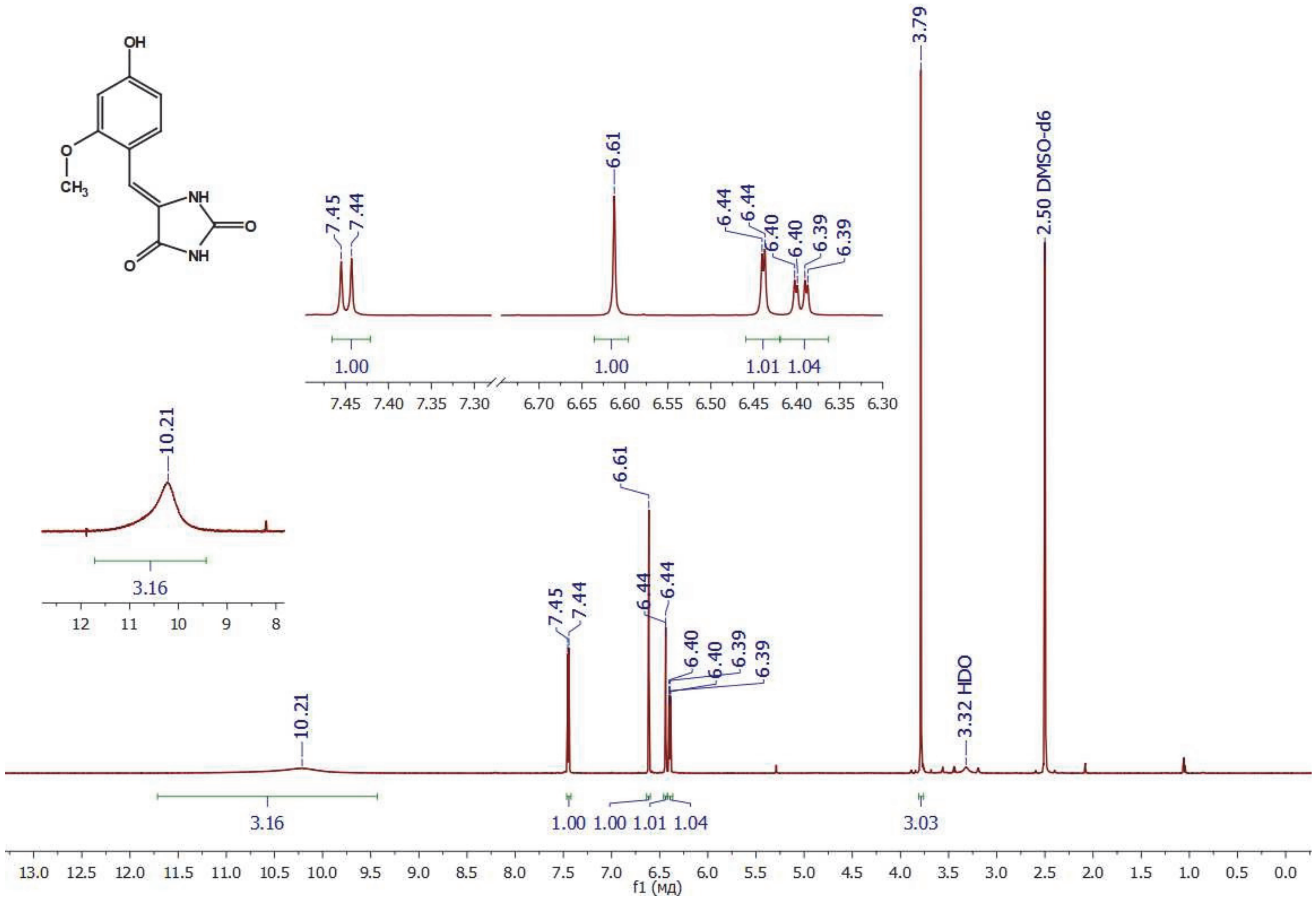
OH
O
CH3
NH
O
NH
O
7.45
7.44
6.61
6.44
6.44
6.40
6.40
6.39
6.39
1.00
1.00
1.01 1.04
7.45 7.40 7.35 7.30
6.70 6.65 6.60 6.55 6.50 6.45 6.40 6.35 6.30
10.21
3.16
12 11 10 9 8
3.79
2.50 DMSO-d6
3.32 HDO
1.00 1.00 1.01 1.04
3.03
13.0 12.5 12.0 11.5 11.0 10.5 10.0 9.5 9.0 8.5 8.0 7.5 7.0 6.5 6.0 5.5 5.0 4.5 4.0 3.5 3.0 2.5 2.0 1.5 1.0 0.5 0.0
f1 (мд)

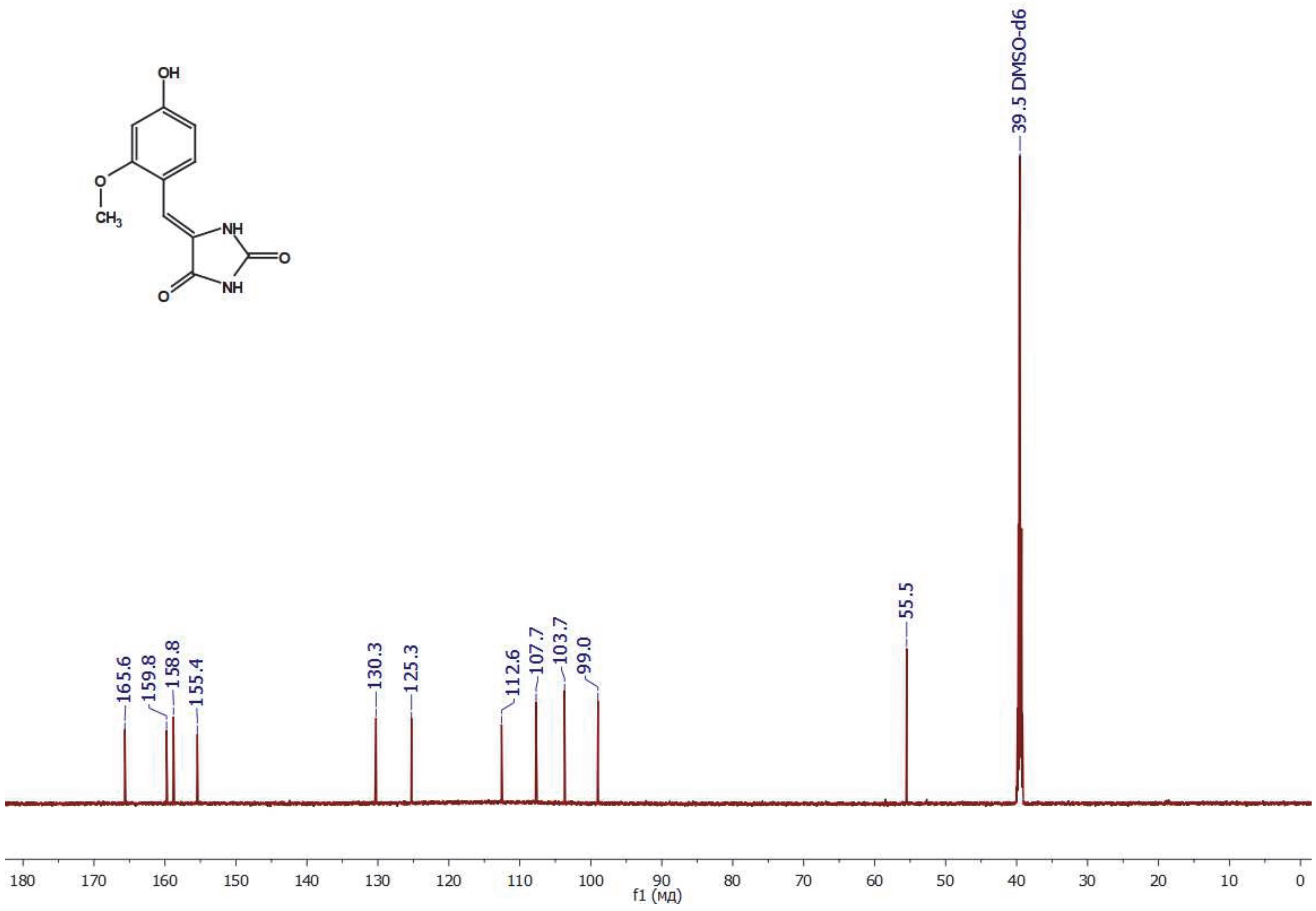
OH
O
CH3
NH
NH
O
O
165.6
159.8
158.8
155.4
130.3
125.3
112.6
107.7
103.7
99.0
55.5
39.5 DMSO-d6
180 170 160 150 140 130 120 110 100 90 80 70 60 50 40 30 20 10 0
f1 (мд)

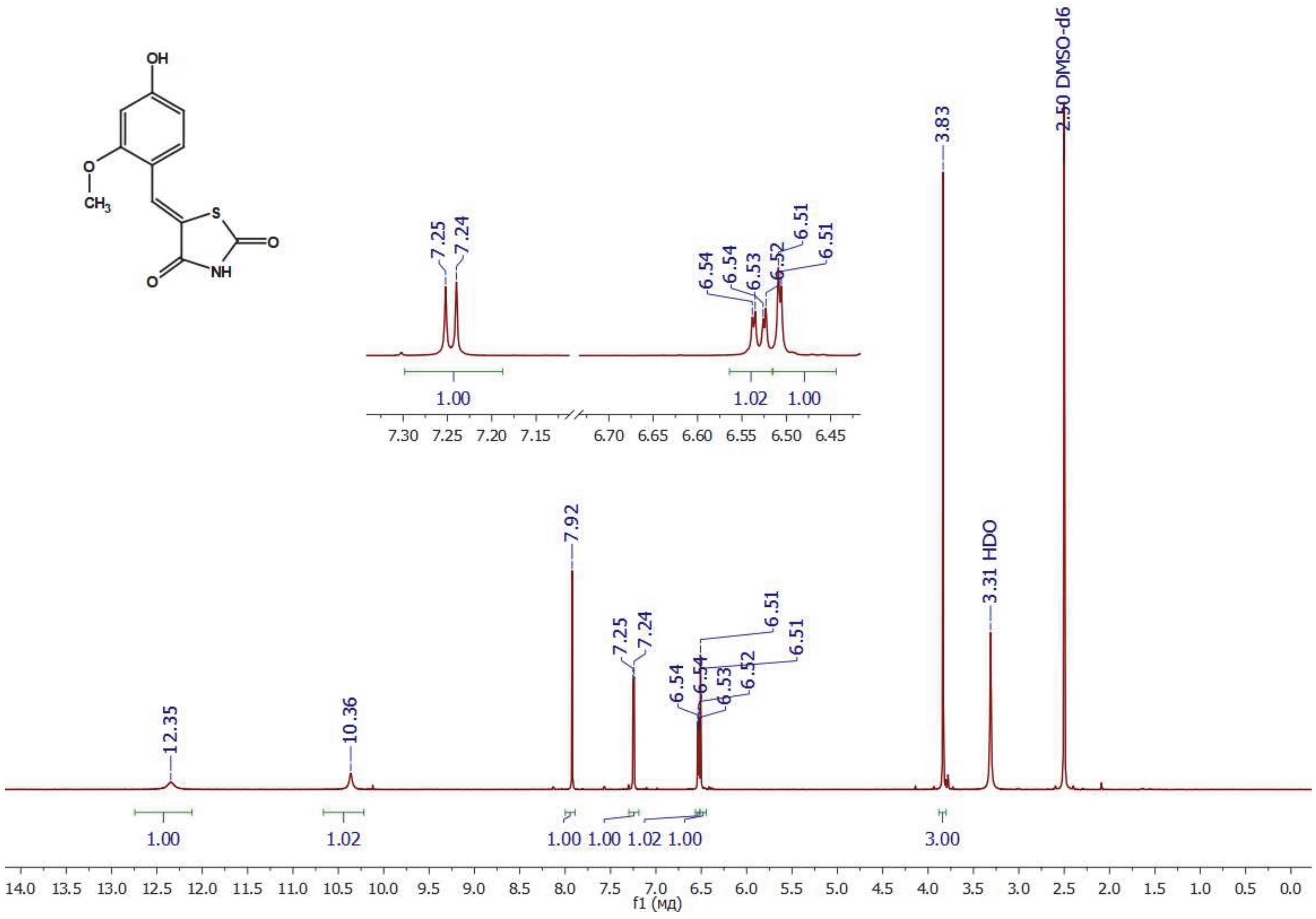
OH
O
CH3
S
O
NH
O
7.25
7.24
6.54
6.54
6.53
6.52
6.51
6.51
1.00
1.02
1.00
7.30 7.25 7.20 7.15
6.70 6.65 6.60 6.55 6.50 6.45
12.35
10.36
7.92
7.25
7.24
6.54
6.54
6.53
6.52
6.51
6.51
3.83
3.31 HDO
2.50 DMSO-d6
1.00
1.02
1.00 1.00 1.02 1.00
3.00
14.0 13.5 13.0 12.5 12.0 11.5 11.0 10.5 10.0 9.5 9.0 8.5 8.0 7.5 7.0 6.5 6.0 5.5 5.0 4.5 4.0 3.5 3.0 2.5 2.0 1.5 1.0 0.5 0.0
f1 (мд)

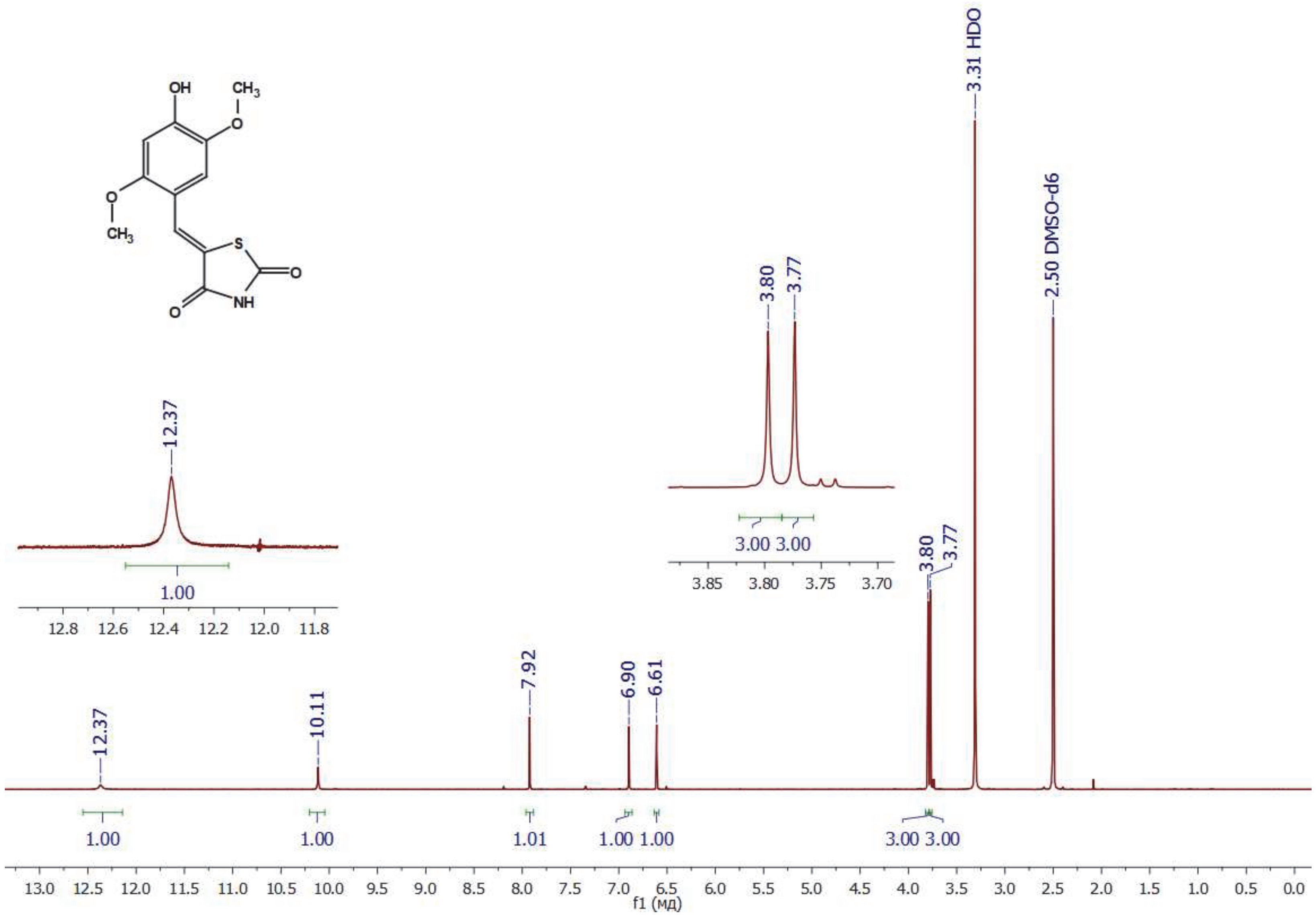

OH
CH3
O
O
CH3
S
O
NH
O
12.37
1.00
12.8 12.6 12.4 12.2 12.0 11.8
3.80
3.77
3.00 3.00
3.85 3.80 3.75 3.70
3.31 HDO
2.50 DMSO-d6
12.37
10.11
7.92
6.90
6.61
1.00
1.00
1.01
1.00 1.00
3.00 3.00
13.0 12.5 12.0 11.5 11.0 10.5 10.0 9.5 9.0 8.5 8.0 7.5 7.0 6.5 6.0 5.5 5.0 4.5 4.0 3.5 3.0 2.5 2.0 1.5 1.0 0.5 0.0
f1 (мд)

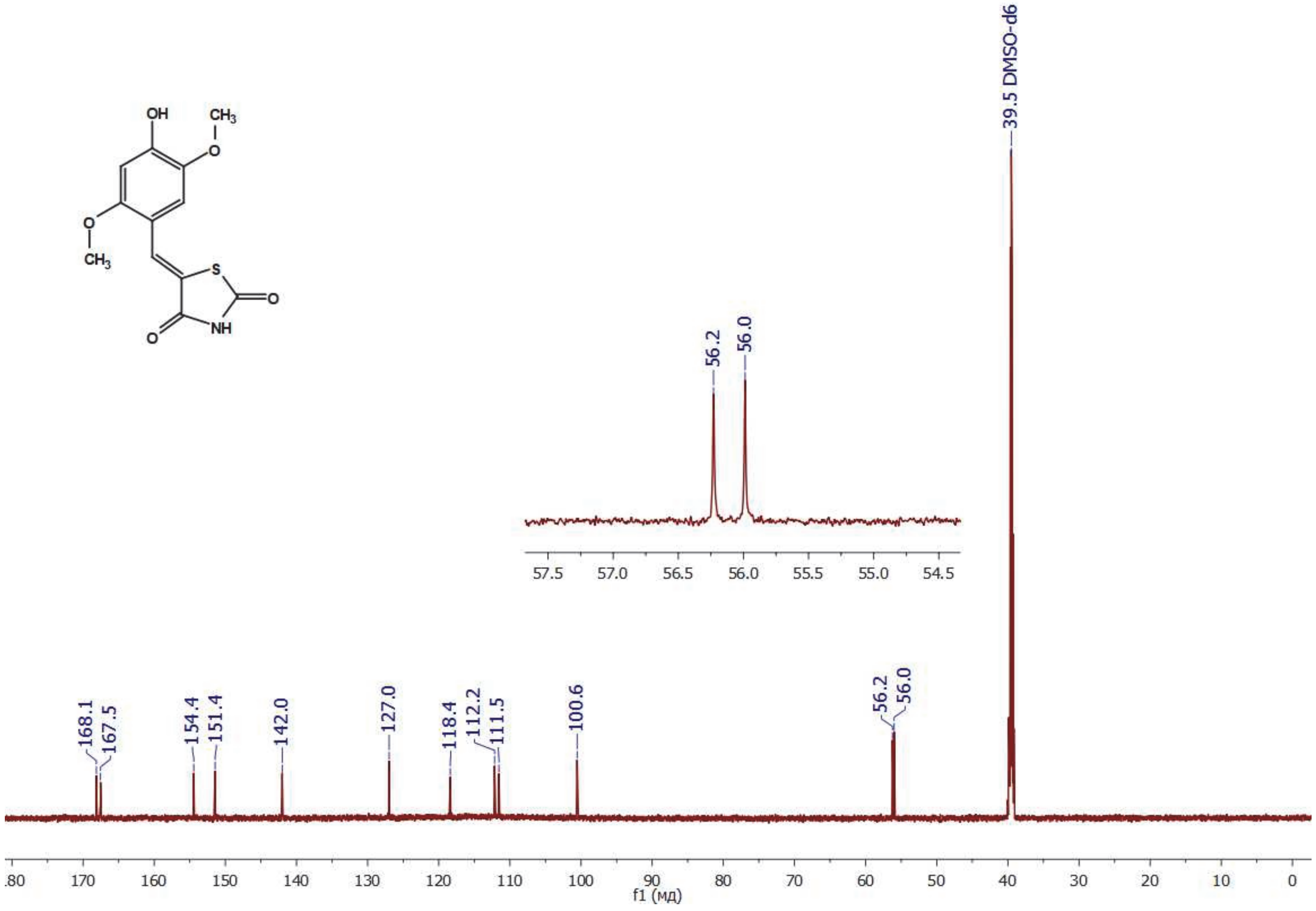

OH
CH3
O
O
CH3
S
O
NH
O
39.5 DMSO-d6
56.2
56.0
57.5
57.0
56.5
56.0
55.5
55.0
54.5
168.1
167.5
154.4
151.4
142.0
127.0
118.4
112.2
111.5
100.6
56.2
56.0
80
170
160
150
140
130
120
110
100
90
80
70
60
50
40
30
20
10
0
f1 (мд)

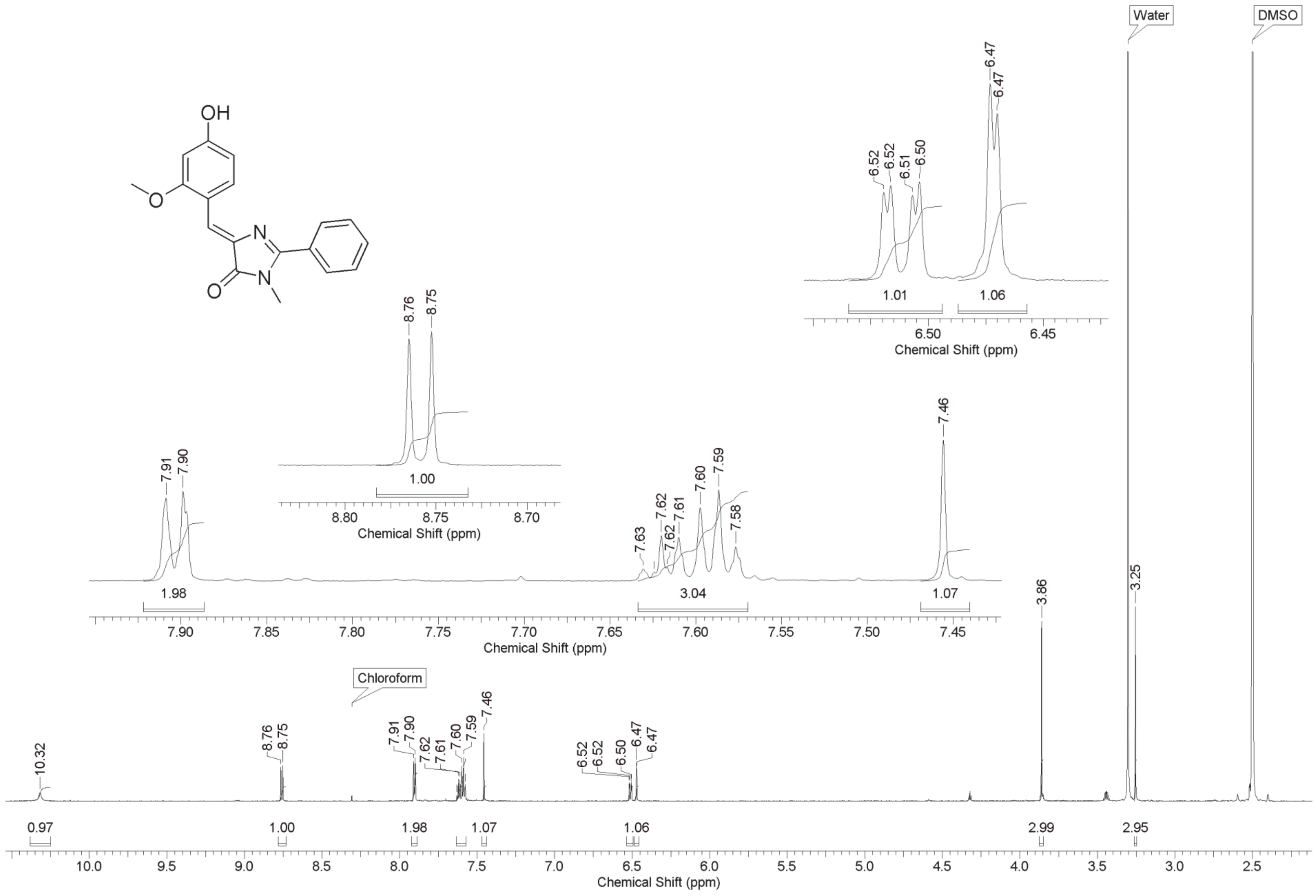

OH
O
N
N
O
Water
DMSO
Chloroform
Chemical Shift (ppm)

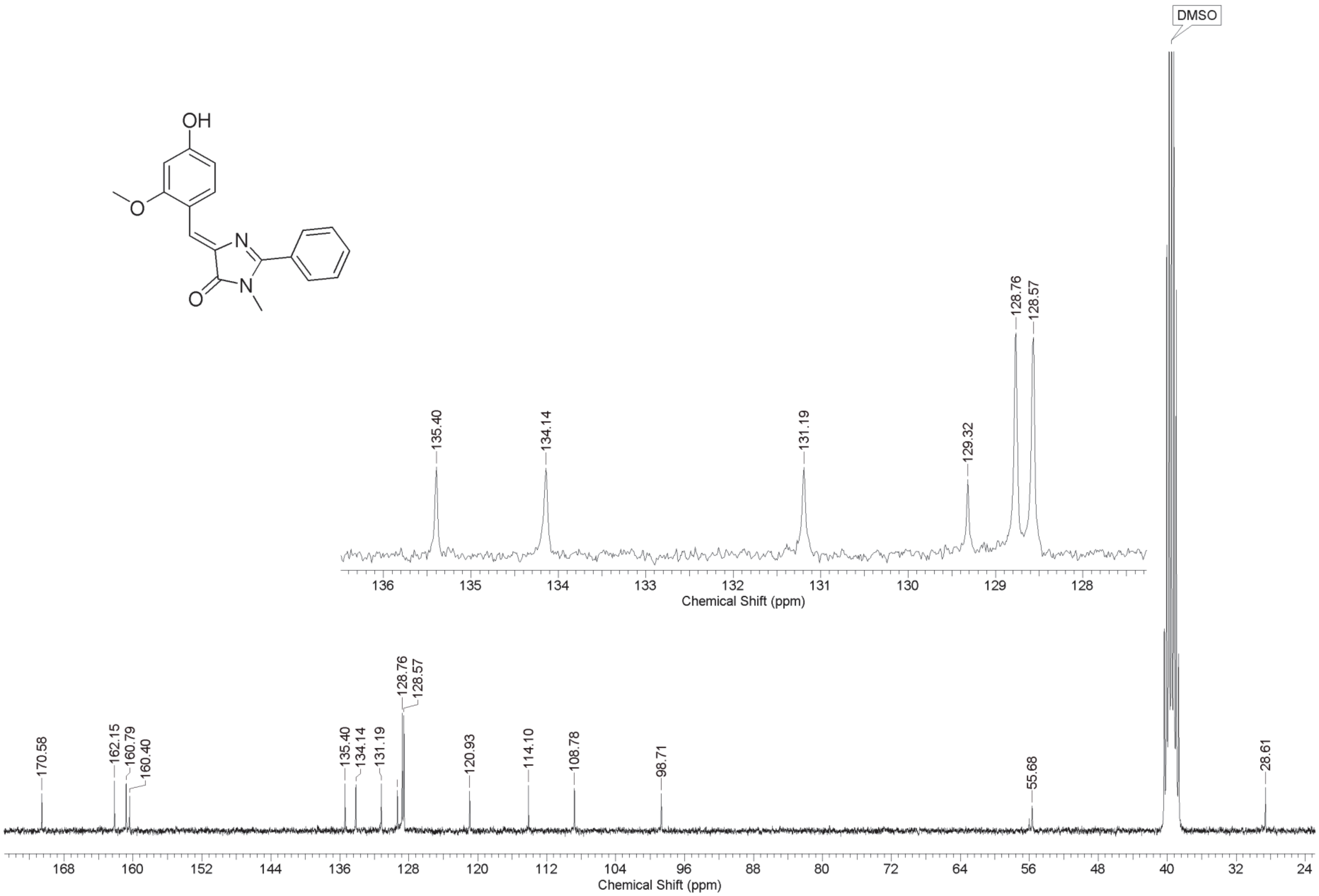
OH
O
N
N
O
DMSO
135.40
134.14
131.19
129.32
128.76
128.57
136
135
134
133
132
131
130
129
128
Chemical Shift (ppm)
170.58
162.15
160.79
160.40
135.40
134.14
131.19
128.76
128.57
120.93
114.10
108.78
98.71
55.68
28.61
168
160
152
144
136
128
120
112
104
96
88
80
72
64
56
48
40
32
24
Chemical Shift (ppm)

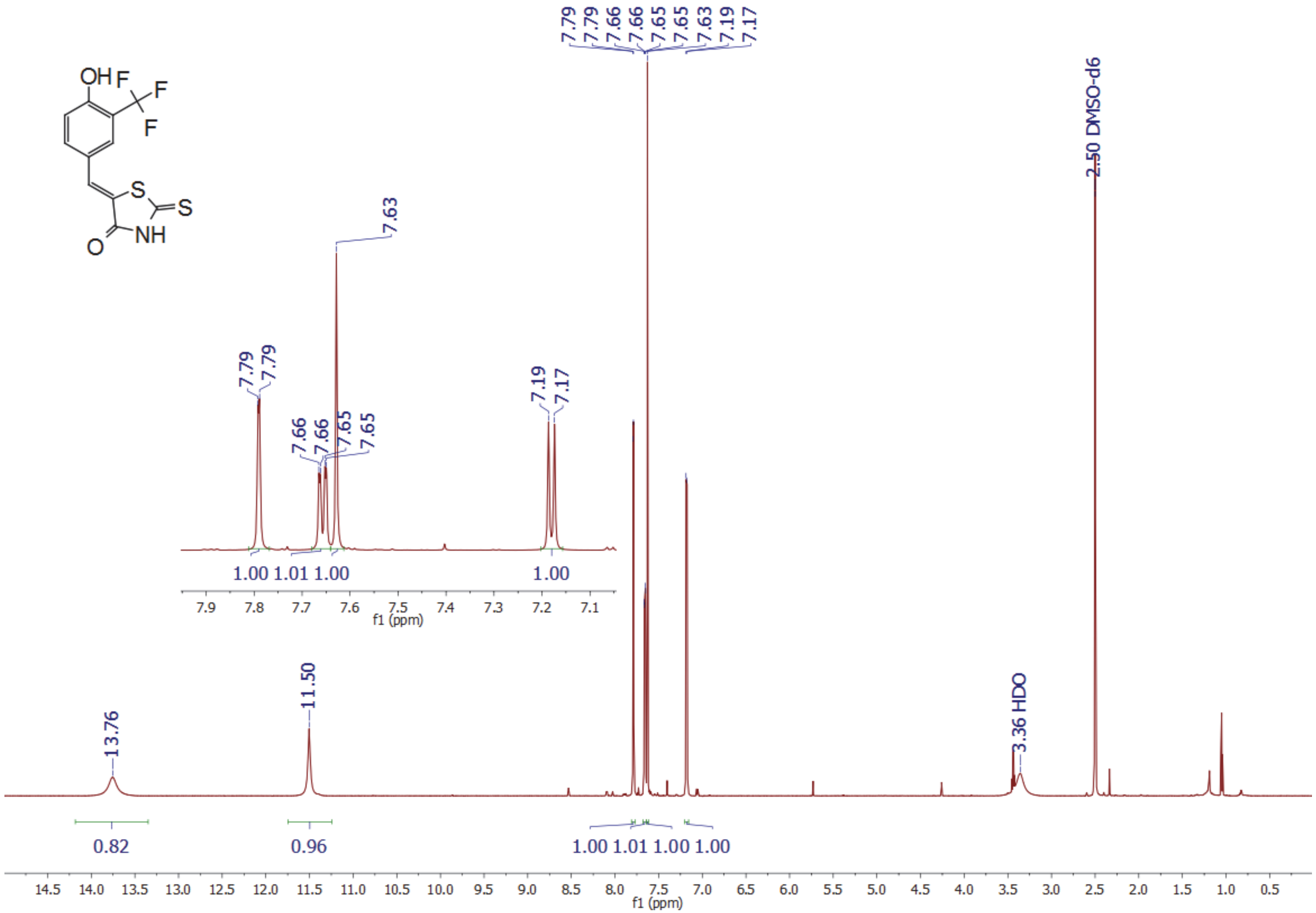
7.79
7.79
7.66
7.66
7.65
7.65
7.63
7.19
7.17
2.50 DMSO-d6
7.63
7.79
7.79
7.66
7.66
7.65
7.65
7.19
7.17
1.00 1.01 1.00
1.00
f1 (ppm)
13.76
11.50
3.36 HDO
0.82
0.96
1.00 1.01 1.00 1.00
f1 (ppm)

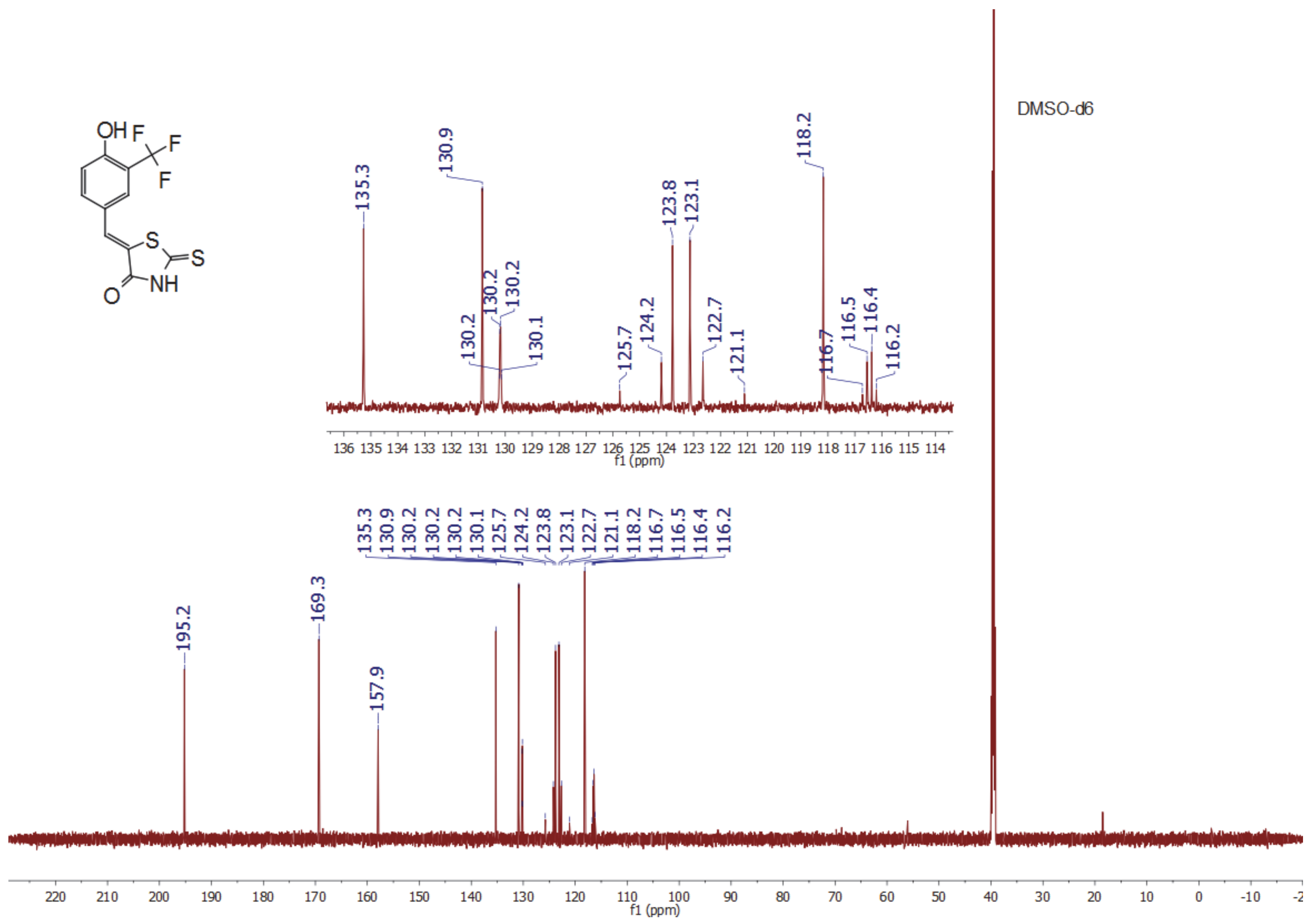
OH
F
F
F
S
S
NH
O
DMSO-d6
135.3
130.9
130.2
130.2
130.2
130.1
125.7
124.2
123.8
123.1
122.7
121.1
118.2
116.7
116.5
116.4
116.2
195.2
169.3
157.9
f1 (ppm)

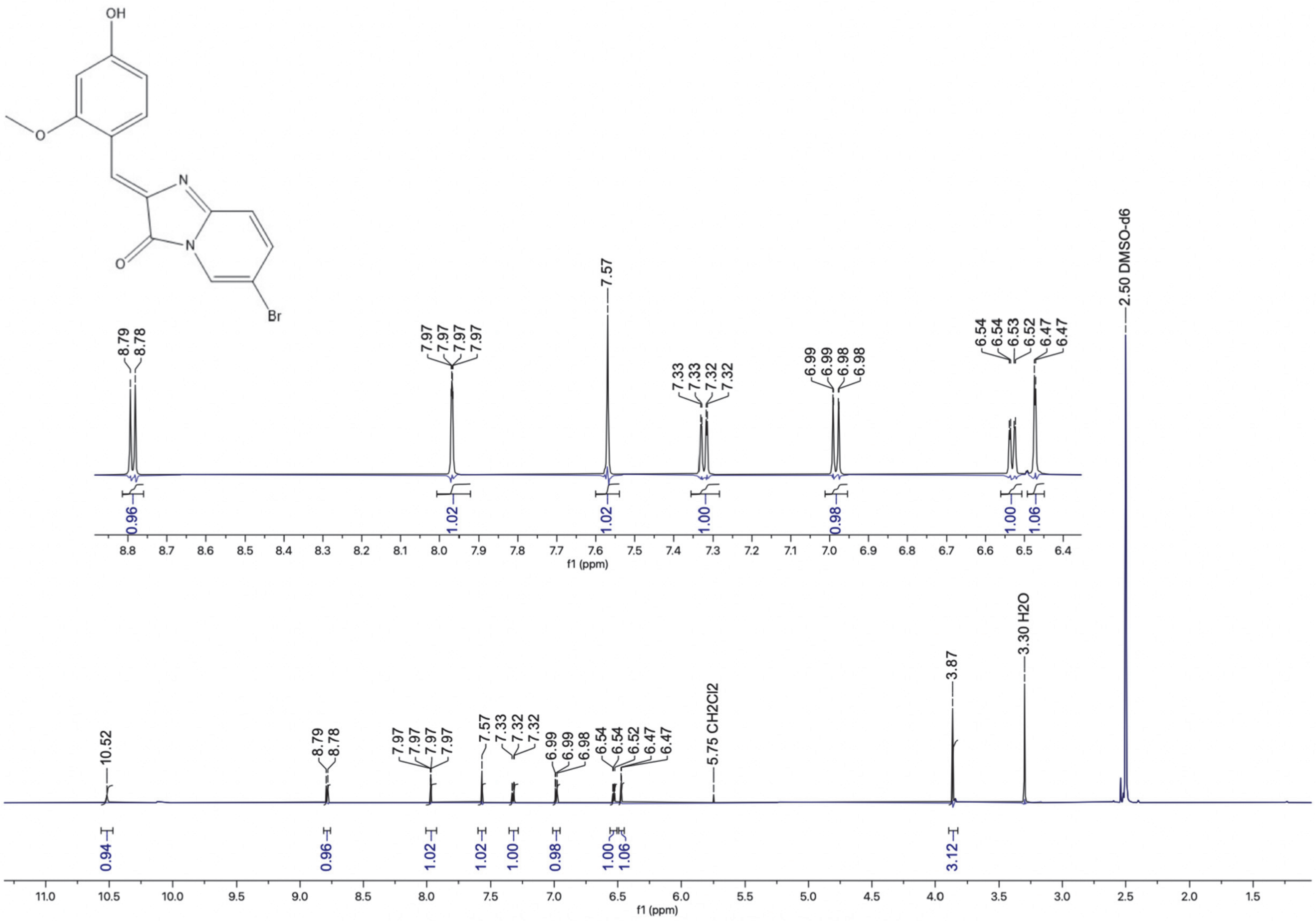
OH
O
N
N
O
Br
8.79
8.78
7.97
7.97
7.97
7.97
7.57
7.33
7.33
7.32
7.32
6.99
6.99
6.98
6.98
6.54
6.54
6.53
6.52
6.47
6.47
2.50 DMSO-d6
0.96
1.02
1.02
1.00
0.98
1.00
1.06
f1 (ppm)
10.52
5.75 CH2Cl2
3.87
3.30 H2O
0.94
3.12
f1 (ppm)

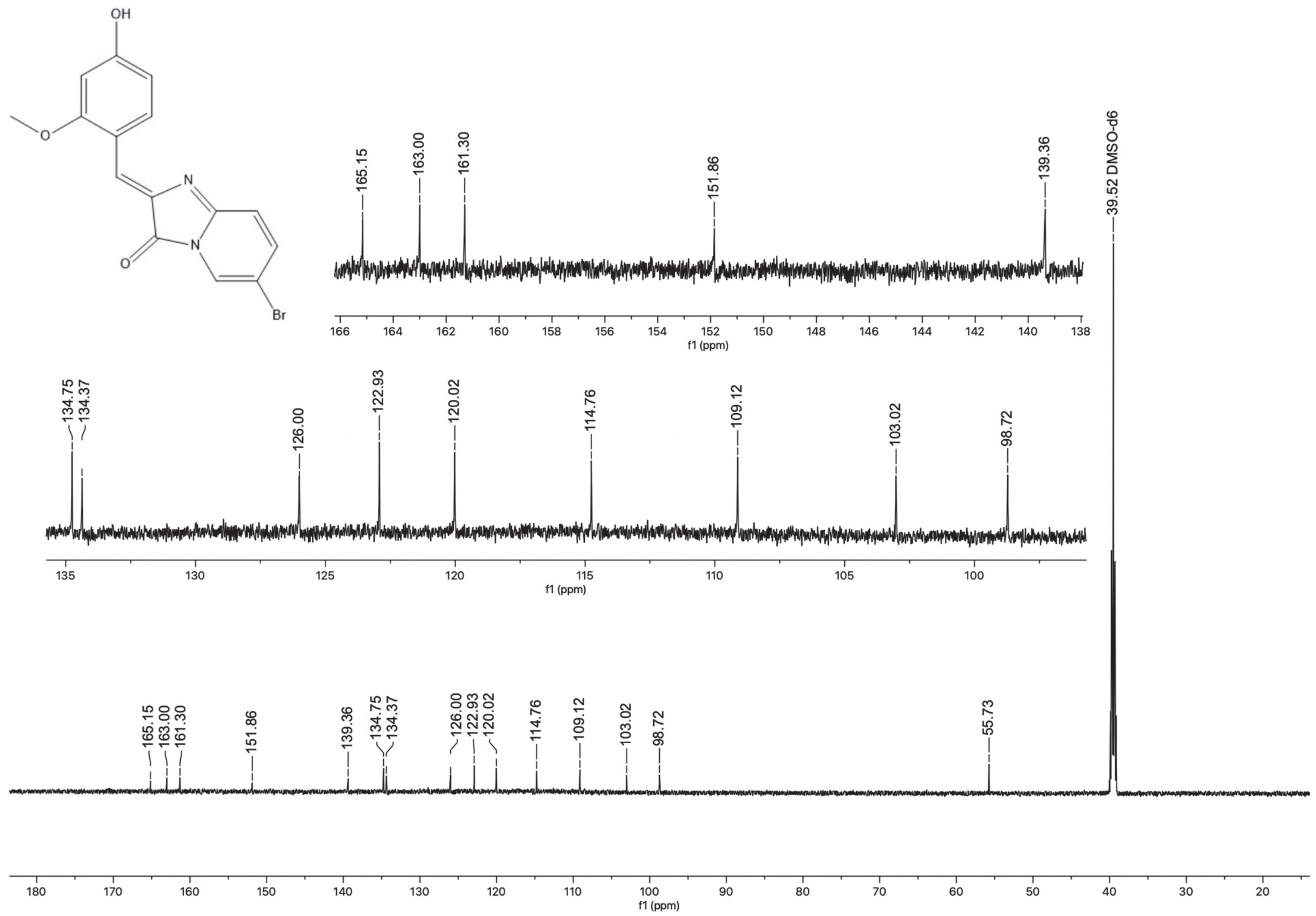

OH
O
N
Br
165.15
163.00
161.30
151.86
139.36
39.52 DMSO-d6
134.75
134.37
126.00
122.93
120.02
114.76
109.12
103.02
98.72
55.73
f1 (ppm)
